# Context-dependent miR-204 and miR-211 affect the biological properties of amelanotic and melanotic melanoma cells

**DOI:** 10.18632/oncotarget.15915

**Published:** 2017-03-06

**Authors:** Marianna Vitiello, Andrea Tuccoli, Romina D’Aurizio, Samanta Sarti, Laura Giannecchini, Simone Lubrano, Andrea Marranci, Monica Evangelista, Silvia Peppicelli, Chiara Ippolito, Ivana Barravecchia, Elena Guzzolino, Valentina Montagnani, Michael Gowen, Elisa Mercoledi, Alberto Mercatanti, Laura Comelli, Salvatore Gurrieri, Lawrence W. Wu, Omotayo Ope, Keith Flaherty, Genevieve M. Boland, Marc R. Hammond, Lawrence Kwong, Mario Chiariello, Barbara Stecca, Gao Zhang, Alessandra Salvetti, Debora Angeloni, Letizia Pitto, Lido Calorini, Giovanna Chiorino, Marco Pellegrini, Meenhard Herlyn, Iman Osman, Laura Poliseno

**Affiliations:** ^1^ Oncogenomics Unit, Core Research Laboratory, Istituto Toscano Tumori (ITT), AOUP, Pisa, Italy; ^2^ Institute of Clinical Physiology (IFC), CNR, Pisa, Italy; ^3^ Laboratory of Integrative Systems Medicine (LISM), Institute of Informatics and Telematics (IIT), CNR, Pisa, Italy; ^4^ University of Siena, Italy; ^5^ Section of Experimental Pathology and Oncology, Department of Experimental and Clinical Biomedical Sciences, University of Firenze, Italy; ^6^ Unit of Histology, Department of Clinical and Experimental Medicine, University of Pisa, Italy; ^7^ Scuola Superiore Sant’Anna, Pisa, Italy; ^8^ Tumor Cell Biology Unit, Core Research Laboratory, Istituto Toscano Tumori (ITT), AOUC, Firenze, Italy; ^9^ New York University, New York, NY, USA; ^10^ The Wistar Institute, Philadelphia, PA, USA; ^11^ Massachusetts General Hospital, Boston, MA, USA; ^12^ MD Anderson Cancer Center, Houston, TX, USA; ^13^ Signal Transduction Unit, Core Research Laboratory, Istituto Toscano Tumori (ITT), AOUS, Siena, Italy; ^14^ Unit of Experimental Biology and Genetics, Department of Clinical and Experimental Medicine, University of Pisa, Italy; ^15^ Fondazione Edo and Elvo Tempia, Biella, Italy

**Keywords:** melanoma, BRAFV600E, ERK pathway, miR-204 family, context-dependency

## Abstract

Despite increasing amounts of experimental evidence depicting the involvement of non-coding RNAs in cancer, the study of BRAFV600E-regulated genes has thus far focused mainly on protein-coding ones. Here, we identify and study the microRNAs that BRAFV600E regulates through the ERK pathway.

By performing small RNA sequencing on A375 melanoma cells and a vemurafenib-resistant clone that was taken as negative control, we discover miR-204 and miR-211 as the miRNAs most induced by vemurafenib. We also demonstrate that, although belonging to the same family, these two miRNAs have distinctive features. miR-204 is under the control of STAT3 and its expression is induced in amelanotic melanoma cells, where it acts as an effector of vemurafenib's anti-motility activity by targeting AP1S2. Conversely, miR-211, a known transcriptional target of MITF, is induced in melanotic melanoma cells, where it targets EDEM1 and consequently impairs the degradation of TYROSINASE (TYR) through the ER-associated degradation (ERAD) pathway. In doing so, miR-211 serves as an effector of vemurafenib's pro-pigmentation activity. We also show that such an increase in pigmentation in turn represents an adaptive response that needs to be overcome using appropriate inhibitors in order to increase the efficacy of vemurafenib.

In summary, we unveil the distinct and context-dependent activities exerted by miR-204 family members in melanoma cells. Our work challenges the widely accepted “same miRNA family = same function” rule and provides a rationale for a novel treatment strategy for melanotic melanomas that is based on the combination of ERK pathway inhibitors with pigmentation inhibitors.

## INTRODUCTION

BRAF is a Ser/Thr protein kinase belonging to the highly oncogenic RAS/RAF/MEK/ERK signaling pathway. Roughly half of melanomas harbor somatic mutations in BRAF. The most frequent mutation is the V600E substitution, which renders the kinase constitutively active as a monomer and independent of RAS-induced dimerization [1]. Identification of this driver mutation has paved the way for development of selective molecularly-targeted inhibitors. BRAFi such as vemurafenib have been shown to increase overall and progression-free survival in metastatic melanoma patients who carry the V600E mutation. However, these inhibitors have significant limitations, including: suboptimal response rates, which occur in setting of adaptive cellular responses [2–6]; a heterogeneous response rate among patient populations; severe side effects that often require treatment termination; invariable development of acquired resistance within 6 months of treatment initiation [7, 8]. Therefore, novel approaches to enhance BRAFi efficacy are still needed [9, 10].

In order to improve BRAFV600E targeting, it is vital to gain a deeper understanding of the genes that are positively and negatively regulated by this kinase. In this work we focus on the identification and characterization of BRAFV600E-regulated microRNAs (miRNAs). While protein-coding genes have been extensively studied, the reported cases of non-coding RNAs that BRAFV600E regulates through the ERK pathway are still very few (the long non-coding RNA *BANCR* (BRAF-activated lncRNA), miR-146a and miR-768-3p are among the few examples [11–13]). In contrast, all classes of long and short non-coding RNAs have recently come to the forefront as crucial regulators of gene expression that play a pivotal role in human cancer [14, 15]. There are also examples of miRNAs being used as drugs or drug targets [16]. Therefore, the thorough study of BRAFV600E-regulated miRNAs is relevant not only in regards to basic RNA biology, but also for its potential translational implications.

Through use of high-throughput techniques such as the sequencing of small RNAs, we were able to identify the full spectrum of miRNAs that in melanoma are regulated by BRAFV600E through the ERK pathway. We then focused on the miRNA family composed by miR-204 and miR-211 and investigated their transcriptional regulation, respective functions and how they interact with vemurafenib. Ultimately, the analysis allowed us to demonstrate that miRNAs belonging to the same family can exert distinct biological roles. Furthermore, we discovered a novel adaptive mechanism in melanotic melanomas, which is elicited by BRAFi/MEKi and needs to be overcome in order to fully unleash their activity.

## RESULTS

### miR-204 is induced by vemurafenib in A375 melanoma cells

**Figure 1:**
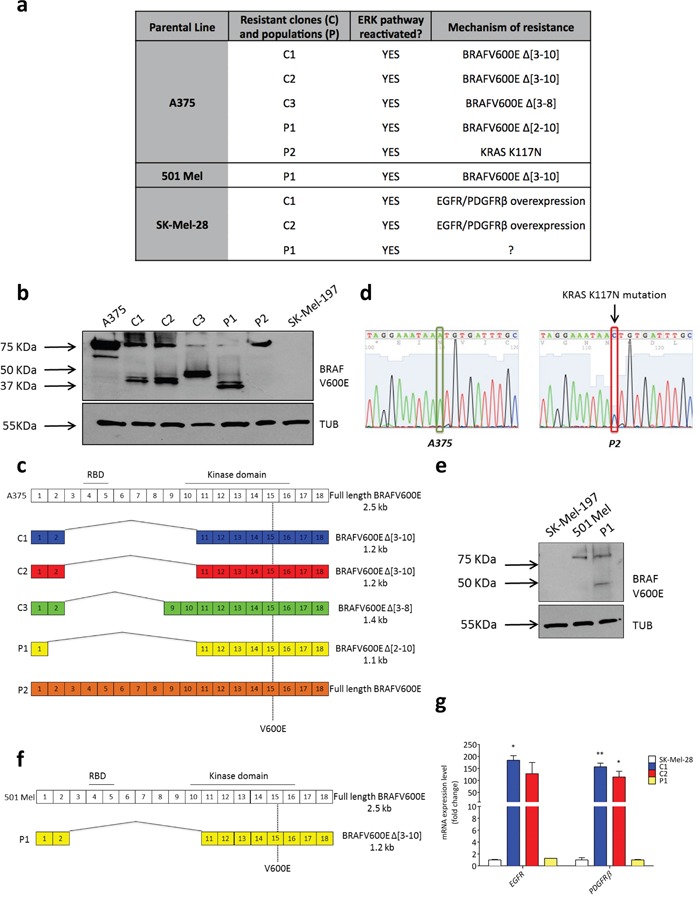
Mechanisms of acquired resistance displayed by vemurafenib-resistant clones and populations obtained from A375, 501 Mel and SK-Mel-28 cells **(a)** Table that lists vemurafenib-resistant clones (C) and populations (P) and the corresponding resistance mechanism. The full list of known alterations that were searched for is reported in Supplementary Table 2. The resistance mechanism of SK-Mel-28 P1 population remains to be discovered. **(b)** Western blot for BRAFV600E protein in A375 parental cell line and vemurafenib-resistant clones (C) and populations (P). SK-Mel-197 cells, which are wt for BRAF, are included as a negative control. Immunoblotting for α-TUBULIN (TUB) is used as loading control. **(c)** Cartoon depicting the BRAFV600E splicing variants identified in A375 vemurafenib-resistant clones and populations. RBD: RAS-binding domain. **(d)** Electropherograms showing a single-nucleotide mutation (A→C) in the *KRAS* gene of A375 P2 resistant population (*right*) compared to A375 parental cell line (*left*), resulting in the K117N amino acid substitution. **(e)** Western blot for BRAFV600E protein in 501 Mel parental cell line and vemurafenib-resistant population (P1). SK-Mel-197 cells, which are wt for BRAF, are included as negative control. Immunoblotting for α-TUBULIN (TUB) is used as loading control. **(f)** Cartoon depicting the BRAFV600E splicing variant identified in 501 Mel P1. **(g)** In SK-Mel-28 C1 and C2 vemurafenib-resistant clones, *EGFR* and *PDGFR*β are over-expressed, as detected by real-time PCR. The graphs represent the mean±SEM of 3 independent experiments. *p<0.05, **p<0.01.

In order to identify the miRNAs that are positively and negatively regulated by BRAFV600E through the ERK pathway, we took three sensitive cell lines that carry the V600E mutation (A375, 501 Mel and SK-Mel-28) and used them to generate individual clones and populations that are resistant to the selective BRAF inhibitor vemurafenib (PLX4720, Supplementary Figure 1-4 and Supplementary Table 1, 2). These resistant lines are characterized by mechanisms of acquired resistance (AR) that, although different, all lead to the reactivation of the ERK pathway (Figure 1a). Specifically, A375 C1, C2, C3 resistant clones and A375 P1 resistant population carry a BRAF splicing variant (Figure 1b–1c); A375 P2 resistant population carries a K117N mutation on KRAS gene (Figure 1d); 501 Mel P1 resistant population carries a BRAF splicing variant (Figure 1e, 1f); Sk-Mel-28 C1 and C2 resistant clones show the over-expression of *EGFR* and *PDGFRbeta* (Figure 1g)

**Figure 2:**
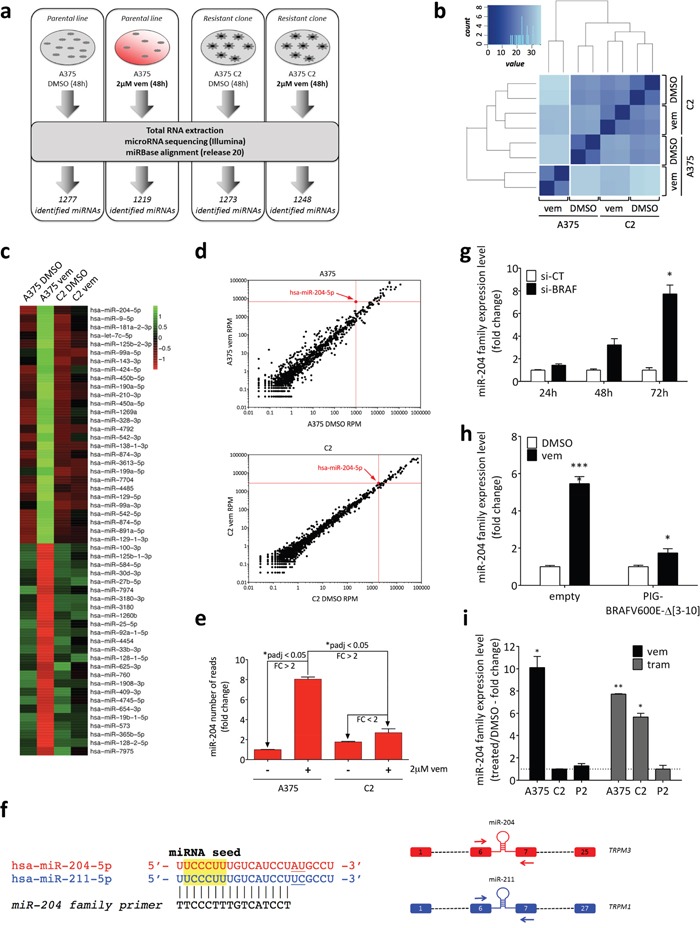
Identification of miR-204 as a microRNA regulated by BRAFV600E through the ERK pathway **(a)** Experimental design of miRNA-seq. A375 parental cell line and A375 C2 vemurafenib-resistant clone were treated with vehicle (DMSO) or 2uM vemurafenib for 48h. RNA was extracted and used to perform the miRNA-seq. **(b)** Sample clustering based on the distance matrix of miRNA profiles. Euclidean metric was used to measure the distance between samples. Darker blue represents higher similarity. **(c)** Heatmap of differentially expressed miRNAs. Variance-stabilized transformed count data is scaled and centered. **(d)** Dotplot of the miRNAs differentially expressed in A375 vemurafenib vs A375 DMSO (*upper*) and in C2 vemurafenib vs C2 DMSO (*lower*). **(e)** miR-204 reads obtained from miRNA-seq, relative to control (A375 treated with DMSO). In both **(d**) and **(e)**, the graphs show that miR-204 levels are induced in A375 vemurafenib, but not in C2 vemurafenib. **(f)** (*left*) Sequence of miR-204 family members, miR-204 and miR-211. The sequence of the “miR-204 family” real-time PCR primer is also reported. (*right*) Schematic representation of miR-204 and miR-211 host genes (*TRPM3* and *TRPM1*, respectively). Red and blue rectangles: exons; dashed lines: other exons/introns; arrows: host gene-specific real-time PCR primers. **(g)** Time course of miR-204 expression levels after the gene-expression inhibition of *BRAF* by siRNA. **(h)** miR-204 levels after 48h of treatment with 2uM vemurafenib in cells stably expressing the BRAFV600E Δ [3–10] splicing variant compared to the empty vector. **(i)** miR-204 expression levels in A375 parental cell line and C2 and P2 vemurafenib-resistant derivatives after treatment with 2uM vemurafenib or 1nM trametinib for 48h. Vemurafenib and trametinib treatments are normalized on the control samples (DMSO, dotted line). The graphs represent the mean±SEM of 3 independent experiments. *p<0.05, **p<0.01, ***p<0.001.

We then performed RNA-sequencing of small RNAs (miRNA-seq) comparing four conditions: parental A375 sensitive cells treated with DMSO or 2uM vemurafenib and A375 C2 resistant clone treated with DMSO or 2uM vemurafenib (Figure 2a, Supplementary Figure 5 and Supplementary Table 3-5). Clustering analysis indicated that the only condition that is different from the others is the one in which vemurafenib is able to inhibit BRAFV600E and block the ERK pathway in A375 parental cells (Figure 2b).

Based on these results, we selected as BRAFV600E-regulated miRNAs those that showed increased or decreased expression levels in A375 cells treated with vemurafenib and comparable levels in the other three conditions. By following the sequential steps summarized in Supplementary Figure 5b, we identified 53 miRNAs, of which 28 are induced and 25 are repressed by vemurafenib (Figure 2c). Among the 28 induced miRNAs, we selected miR-204-5p (miR-204), as it is the most induced one among those highly expressed (Figure 2d, 2e). According to our miRNA-seq results, this miRNA comes in two isomiRs, one that corresponds to the canonical 22nt sequence and the other that is 1nt longer at the 3′end (Supplementary Figure 6a). Furthermore, it is in the same family as miR-211-5p (miR-211, Figure 2f, left), which is not expressed in A375 cells, according to our miRNA-seq experiment and confirmed by previous studies [17]. Both miRNAs are intronic (they are hosted in intron 6 of *TRPM3* (miR-204) and *TRPM1* (miR-211) (Figure 2f, right)) and are co-expressed with their host genes [18, 19].

Since miR-204 and miR-211 have very similar sequences, the measurement of their individual levels by real-time PCR can be technically difficult. Therefore, we developed 2 alternative strategies: firstly, we designed a primer that measures their cumulative levels (miR-204 family primer, Figure 2f, left); secondly, in order to assess individual contributions, we opted for measuring the levels of the host genes using *TRPM3-* and *TRPM1*-specific primers (Figure 2f, right).

The ability of vemurafenib to induce miR-204 and *TRPM3* in A375 cells was confirmed by performing real-time PCR on the same samples used for the miRNA-seq (Supplementary Figure 6b, 6c), as well as using the array data reported in ref [20] (Supplementary Figure 6d). Furthermore, a time-course experiment allowed us to establish that the induction of miR-204 starts 24h after vemurafenib treatment initiation and reaches its plateau at 48h (Supplementary Figure 6e). Finally, a dose response curve indicated that vemurafenib administered at as low as 0.02uM is sufficient to induce miR-204 (Supplementary Figure 6f).

### miR-204 is negatively regulated by BRAFV600E through the ERK pathway

In order to confirm that the induction of miR-204 is a consequence of the selective inhibition of BRAFV600E caused by vemurafenib and not an off-target effect, we performed several experiments. First, we observed that, similarly to the chemical inhibition through vemurafenib, the knock-down of *BRAF* through RNA interference causes an induction of miR-204 levels in A375 cells (Figure 2g and Supplementary Figure 7a, 7b). Second, we infected A375 cells with the BRAFV600EΔ [3–10] splicing variant, which is found in the A375 C2 clone (Figure 1a-1c). In agreement with the data reported by Basile and colleagues [21], the expression of this variant was sufficient to render A375 cells insensitive to vemurafenib (Supplementary Figure 7c-7e), and moreover it prevented the induction of TRPM3 miR-204 upon vemurafenib treatment (Figure 2h and Supplementary Figure 7f). Third, in MeWo and SK-Mel-197 cells, which are BRAF wild-type and hence insensitive to BRAFi, we did not observe any induction of the miR-204 family upon vemurafenib treatment (Supplementary Figure 8a). Comparably, we found that miR-204 is not induced upon treatment of A375 cells with dacarbazine, an alkylating agent with a distinct mechanism of action compared to vemurafenib (Supplementary Figure 8b, 8c).

Next, we assessed whether the induction of miR-204 caused by vemurafenib can be attributable to blocking of the ERK signaling pathway. To this end, we examined the A375 C2 clone and the A375 P2 population. Both these resistant lines are insensitive to vemurafenib due to the reactivation of the ERK pathway, but the alterations that they carry are different (Figure 1a-1d). Consistent with the hypothesis that inhibition of the ERK pathway is crucial in the induction of miR-204, both the C2 and the P2 cells were unable to induce the miRNA upon vemurafenib treatment (Figure 2i and Supplementary Figure 9a). We then inhibited the pathway one step downstream of BRAF using the MEK inhibitor trametinib and found that miR-204 is induced in A375 cells that are sensitive to this drug and in the A375 C2 clone, which, although being resistant to vemurafenib, retains sensitivity to MEKi. Conversely, the P2 population, which is cross-resistant to MEKi, did not show any induction of miR-204 not only when treated with vemurafenib, but also when treated with trametinib (Figure 2i, Supplementary Figure 9b, 9c and refs [22, 23]).

Altogether, the data presented thus far indicate that miR-204 is negatively regulated by BRAFV600E through the ERK pathway and is induced by BRAFi/MEKi.

### BRAFi/MEKi induce miR-204 and miR-211 in a mutually exclusive fashion

**Figure 3:**
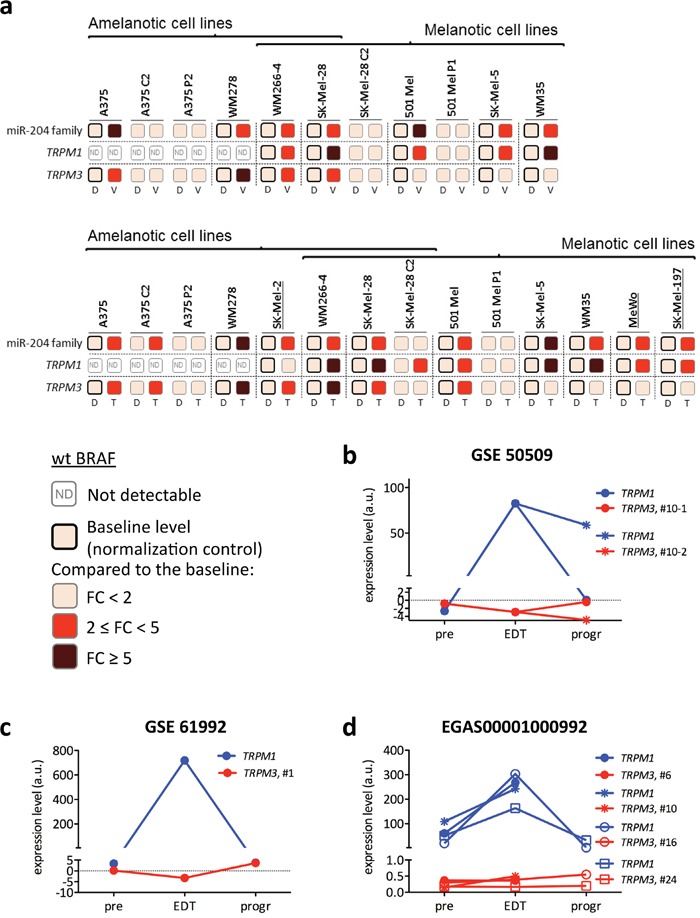
Induction of *TRPM3*/miR-204 and *TRPM1*/miR-211 upon treatment of melanoma cell lines and metastatic melanoma patients with BRAFi and/or MEKi **(a)** Fold induction of miR-204 family, *TRPM1* and *TRPM3* upon 48h of 2uM vemurafenib (V, upper) or 1nM trametinib treatment (T, lower) of the indicated BRAFV600E and wt BRAF (underlined) melanoma cell lines. Vemurafenib-resistant clones and populations are used as negative controls (when the drugs cannot function, there is no miRNA induction). D: DMSO. The expression levels of miR-204 family, *TRPM1* and *TRPM3* in each parental line treated with DMSO is taken as baseline and used as normalization control. Fold changes are therefore represented as increases over the baseline (a darker color means a higher fold change). **(b-d)** Expression levels of *TRPM1* (blue) and *TRPM3* (red) in bioptic samples collected from metastatic melanoma patients at 3 time points: before the beginning of the treatment (pre), early on during treatment (EDT) and at progression (progr). **(b)** In the GSE 50509 dataset, patient #10 was treated with the BRAFi dabrafenib and 2 different tumor sites were analyzed at progression. **(c)** In the GSE 61992 dataset, patient #1 was treated with the BRAFi dabrafenib and the MEKi trametinib. **(d)** In the EGAS00001000992 dataset, patients #6, #10 and #16 were treated with the BRAFi dabrafenib and the MEKi trametinib, while patient #24 was treated with the BRAFi vemurafenib.

Since miR-204 and miR-211 belong to the same family, we tested whether the inhibition of the ERK pathway causes miR-211 induction as well. Indeed, we found that in melanoma cell lines carrying the BRAFV600E mutation (WM266-4, SK-Mel-28, 501 Mel, SK-Mel-5 and WM35 in Figure 3a) treatment with both vemurafenib and trametinib does cause *TRPM1* induction. In melanoma cell lines that are wild type for BRAF (MeWo and SK-Mel-197 in Figure 3a) trametinib treatment also causes *TRPM1* induction. Furthermore, we found that, in vemurafenib-resistant cells such as 501 Mel P1 and SK-Mel-28 C2, *TRPM1* basal levels are comparable to those of parental cells and fail to be induced upon vemurafenib/trametinib treatment (Figure 3a; see also ref [24]). These data suggest that *TRPM1* and *TRPM3* induction share common features.

miR-211 induction was confirmed *in vivo* by analyzing *TRPM1* levels in tumor biopsies obtained from patients with metastatic melanoma who eventually progressed on treatment with BRAFi and/or MEKi. Biopsies were collected at three time points: before the beginning of the treatment (pre), early on during treatment (14-16 days after the beginning of the treatment, EDT) and at resistance (when the disease starts to progress on treatment, progr). Out of a total of 14 cases belonging to three different datasets (GSE 50509 (n=2), GSE 61992 (n=1), EGAS00001000992 (n = 11, [4])), six were found to display the expected trend in *TRPM1* expression: induction early on during treatment (EDT), decrease to basal levels at time of resistance (progr) (Figure 3b, blue lines).

After establishing that *TRPM3*/miR-204 and *TRPM1*/miR-211 are both induced by BRAFi and MEKi, we tested whether they are co-induced or not. We looked for a positive correlation in the expression levels of *TRPM1* and *TRPM3* in the melanoma cases profiled by microarray in ref [25] (n=45), ref [26] (n=56), ref [27] (n=28), ref [28] (n=214) and at www.cbioportal.org, however no correlation was discovered (Supplementary Figure 10a-10e, left panels and Supplementary Table 6). Furthermore, using melanoma cell lines and patients data, we found that the induction of the two miRNAs is in fact mutually exclusive. Namely, when both *TRPM3*/miR-204 and *TRPM1*/miR-211 are expressed, *TRPM1*/miR-211 is the only one that gets induced (refer to 501 Mel, SK-Mel-5, WM35, MeWo and SK-Mel-197 in Figure 3a and to Figure 3b-3d). Conversely, *TRPM3*/miR-204 is induced when it is the only one expressed (refer to A375 and WM278 in Figure 3a). These results suggest that, downstream of the ERK pathway, the transcriptional regulation of the two miRNAs is different.

### miR-211 is under the transcriptional control of MITF and miR-204 is under the transcriptional control of STAT3

**Figure 4:**
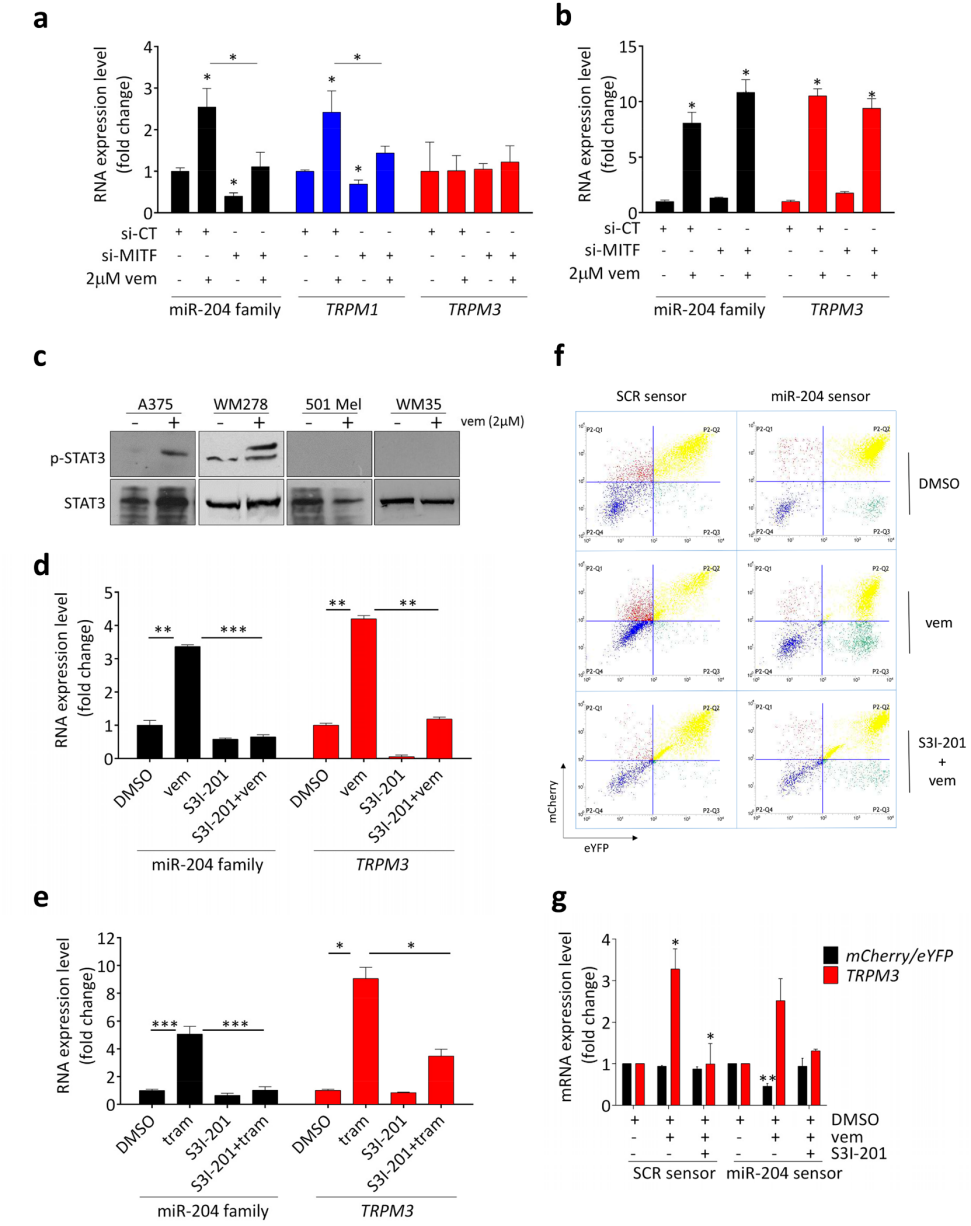
Upon treatment with vemurafenib or trametinib, *TRPM1*/miR-211 induction is MITF-dependent, while *TRPM3*/miR-204 induction is STAT3-dependent **(a)** si-MITF prevents *TRPM1*/miR-211 induction upon vemurafenib treatment in 501 Mel cells. The cells were transfected with si-CT or si-MITF and 24h later they were exposed to vehicle (DMSO) or 2uM vemurafenib for additional 48h. **(b)** si-MITF does not prevent *TRPM3*/miR-204 induction upon vemurafenib treatment in A375 cells. The cells were transfected with si-CT or si-MITF and 24h later they were exposed to vehicle (DMSO) or 2uM vemurafenib for additional 48h. **(c)** Upon 48h of treatment with 2uM vemurafenib, STAT3 phosphorylation is induced in A375 and WM278 cells *(left)*, but not in 501 Mel and WM35 cells *(right)*. **(d-e)** The induction of *TRPM3*/miR-204 caused by 48h treatment with 2uM vemurafenib **(d)** or 1nM trametinib **(e)** is impaired by the concomitant treatment with 4uM of the STAT3 inhibitor S3I-201. **(f-g)** A375 cells that stably express the inducible pTRE-TIGHT-BI-RY miR-204 sensor were treated with 2uM vemurafenib ± 4uM S3I-201 for 48h and then with 2 ug/ml doxycycline for additional 48h. Upon vemurafenib treatment, the increase in endogenous *TRPM3*/miR-204 levels causes a decrease in mCherry fluorescence **(f)**, as well as in the *mCherry*/*eYFP* ratio **(g)**. Upon the combined vemurafenib plus S3I-201 treatment, *TRPM3*/miR-204 induction is blunted and mCherry protein and mRNA return to basal levels. The graphs represent the mean±SEM of 3 independent experiments. *p<0.05, **p<0.01, ***<0.001.

The transcriptional regulation of *TRPM1*/miR-211 has been extensively studied. As a melanocytic lineage-specific miRNA (Supplementary Figure 11a) miR-211 is a well-established transcriptional target of MITF, the master regulator of the melanocytic lineage (see Supplementary Figure 10a-10e, middle panels, where the positive correlation between *TRPM1* and *MITF* levels is shown; see also, for example, ref [29]). In turn, MITF is inhibited by the ERK pathway and hence activated by vemurafenib [3, 30]. Consistent with these findings, we observed that upon treatment with vemurafenib, *TRPM1* is induced with other genes that belong to the “MITF signature” (Supplementary Figure 12a). Furthermore, we were able to demonstrate that the vemurafenib-mediated induction of *TRPM1* is in fact MITF-dependent, by showing that in 501 Mel, SK-Mel-5 and WM35 cells it is prevented by the knock-down of *MITF* mediated by siRNA transfection (Figure 4a and Supplementary Figure 12b, 12c).

In light of the mutually exclusive induction of *TRPM1*/miR-211 and *TRPM3*/miR-204 upon treatment with vemurafenib/trametinib, our first step in the analysis of *TRPM3*/miR-204 transcriptional regulation was to examine the involvement of MITF. We discovered that contrary to the melanocytic lineage restricted expression of miR-211, miR-204 expression is much more widespread (Supplementary Figure 11b). Furthermore, we could not find a correlation between *MITF* and *TRPM3* levels in any of the datasets analyzed (Supplementary Figure 10a-10e, right panels). It was also noted that MITF levels are much lower in the cell lines where *TRPM3*/miR-204 is induced compared to those where *TRPM1*/miR-211 is induced (Supplementary Figure 13). Finally, we demonstrated that the induction of *TRPM3*/miR-204 upon vemurafenib treatment is not MITF-mediated by showing that it is not impaired by si-MITF (Figure 4b; see also ref [31]).

Once MITF was ruled out, we looked for other candidates that could serve as possible links between *TRPM3*/miR-204 and the ERK pathway. It has been recently shown that in melanoma cells the inhibition of the ERK pathway can cause the activation of STAT3 [32–35]. Furthermore, STAT3 binding sites have been identified within the *TRPM3* genomic locus [36]. Therefore, we assessed whether *TRPM3*/miR-204 induction occurs through STAT3. We observed that upon vemurafenib and trametinib treatment, STAT3 is phosphorylated in the cells where *TRPM3* and miR-204 are induced (i.e. A375 and WM278) but not in those where they are not induced (i.e. 501 Mel and WM35) (Figure 4c and Supplementary Figure 12d, 12e). Moreoever, we established that the co-treatment of A375 cells with the selective STAT3 inhibitor S3I-201 abolishes the upregulation of *TRPM3*/miR-204 induced by vemurafenib and trametinib (Figure 4d, 4e and Supplementary Figure 14a).

Additionally, this effect was detected by means of a miR-204 sensor in which the expression of the mCherry reporter is inversely proportional to miR-204 levels, while the levels of the eYFP reporter are used as a normalizer (Supplementary Figure 15a-15d). We found that the vemurafenib-induced increase in endogenous *TRPM3*/miR-204 levels in turn causes a decrease in mCherry fuorescence (Figure 4f) and mCherry/eYFP mRNA ratio (Figure 4g). However, when vemurafenib is combined with S3I-201, the induction is blunted and mCherry levels are restored (see also Supplementary Figure 15e).

Finally, we aimed to identify the upstream regulator of STAT3. We tested whether the induction of *TRPM3*/miR-204 caused by vemurafenib is reversed by the concomitant treatment with inhibitors of JAK2, SRC or PI3K respectively, which are all well-known STAT3 activators [32, 34, 35, 37]. As shown in Supplementary Figure 14b-14d, we found that the JAK2/STAT3 double inhibitor WP1066 is able to inhibit STAT3 phosphorylation and reverse miR-204 induction upon vemurafenib treatment, while the JAK2 single inhibitor AZD1480 (Supplementary Figure 14e-14g), the SRC inhibitor PP1 and the PI3K inhibitor wortmannin are not (Supplementary Figure 14h). Taken together, these results indicate that, downstream of the ERK pathway, the transcriptional regulation of miR-204 and miR-211 is different, with the latter being under the control of MITF, while the former is under the control of STAT3. The data also suggest-that JAK2, SRC and PI3K are all dispensable for the ERK pathway-dependent STAT3 activation [33].

### miR-204 mediates the anti-motility activity of vemurafenib in amelanotic melanoma cells

In order to study the biological effects of miR-204 and miR-211 induction upon vemurafenib/trametinib treatment, we used the biological context in which this induction actually happens. Specifically, we discovered that the cell lines where *TRPM3*/miR-204 is induced are amelanotic (A375, WM278 and SK-Mel-2 in Figure 3a), while the cell lines where *TRPM1*/miR-211 is induced are melanotic (501 Mel, SK-Mel-5, WM35, MeWo and SK-Mel-197 in Figure 3a). Interestingly, WM266-4 and SK-Mel-28, the two cell lines where *TRPM3*/miR-204 and *TRPM1*/miR-211 are co-induced, are in fact “hybrid” cell lines, because they are not pigmented and yet contain melanosomes [38, 39]. Therefore, we focused on amelanotic and melanotic cells for the study of miR-204 and miR-211, respectively.

**Figure 5:**
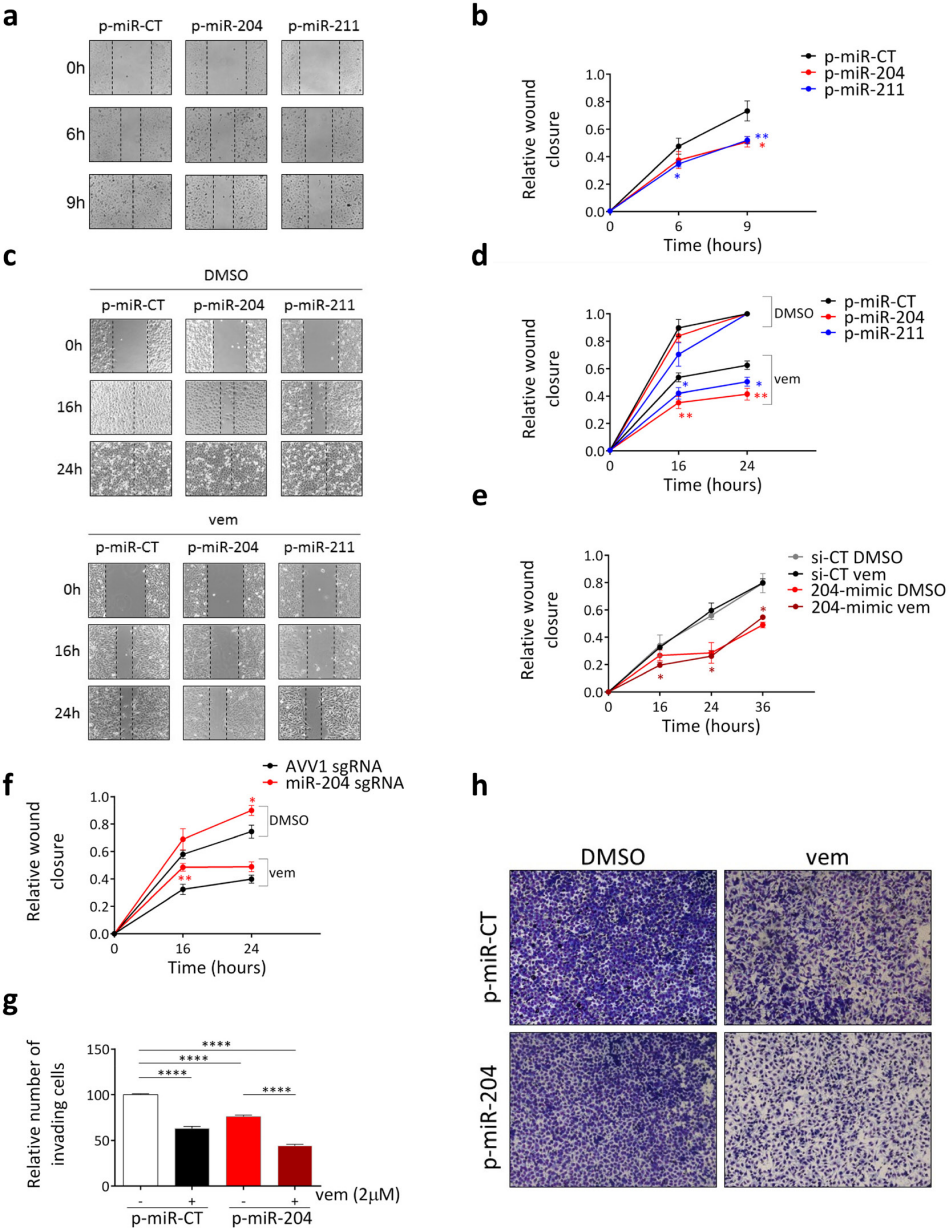
miR-204 mediates the anti-motility activity of vemurafenib in amelanotic melanoma cells **(a-b)** Wound closure of A375 cells that stably over-express a control miRNA (p-miR-CT, black), miR-204 (p-miR-204, red) or miR-211 (p-miR-211, blue). **(c-d)** Wound closure of A375 cells that stably over-express a control miRNA (p-miR-CT, black), miR-204 (p-miR-204, red) or miR-211 (p-miR-211, blue) and were treated for the indicated time points with vehicle (DMSO) or 2uM vemurafenib. Before being subjected to the assay, the cells were pretreated with vemurafenib for additional 24h. **(e)** Wound closure of A375 C2 vemurafenib-resistant cells that were transiently transfected with si-CT (black and grey) or 204-mimic (red and dark red), treated with vehicle (DMSO) or 2uM vemurafenib for 24h and then subjected to the wound healing assay for the indicated time points. **(f)** Wound closure of A375 cells that stably express a control AVV1 sgRNA (black) or a miR-204 sgRNA (red) and that were treated for the indicated time points with vehicle (DMSO) or 2uM vemurafenib. The cells were pretreated for 72h with doxycycline in order to induce the expression of Cas9 and hence the disruption of miR-204 gene, with the consequent dowregulation of the endogenous mature miRNA levels. **(g-h)** Matrigel invasion assay performed on A375 cells that stably over-express a control miRNA (p-miR-CT, white and black) or miR-204 (p-miR-204, red and dark red) and that were treated with 2uM vemurafenib for 6h. The graphs represent the mean±SEM of 3 independent experiments. *p<0.05, **p<0.01, ****<0.0001.

miR-204 has been shown to inhibit cell motility in many tumor types and the ability of miR-211 to inhibit cell motility in melanoma is well established (please, refer to Supplementary Table 7 for references). Furthermore, vemurafenib itself is known to negatively affect the motility of melanoma cells [40]. Therefore, we evaluated whether miR-204 acts as an effector of the anti-motility activity exerted by BRAFi and MEKi on melanoma cells. Using a wound healing assay, we found that, similarly to the over-expression of miR-211 which was taken as positive control, the over-expression of miR-204 does cause an impairment in the ability of A375 melanoma cells to migrate (Figure 5a, 5b and Supplementary Figure 16a, 16b).

We also discovered that miR-204 over-expression potentiates the activity of vemurafenib in A375 cells (Figure 5c, 5d), as well as trametinib in SK-Mel-2 cells (Supplementary Figure 17a). Remarkably, miR-204 over-expression causes a decrease in cell motility even in vemurafenib-resistant A375 C2 cells (Figure 5e). However, in this setting the potentiation in presence of vemurafenib is not observed, which confirms that it depends on the induction of endogenous miR-204 levels by the drug.

The evidence that vemurafenib relies on miR-204 in order to exert its anti-motility activity was obtained in a miR-204 knock-down experiment performed by taking advantage of the CRISPR-Cas9 technology. An sgRNA that targets the Cas9 enzyme against the loop of pre-miR-204 was used to disrupt the miR-204 gene and subsequently cause dowregulation of the endogenous mature miRNA levels (Supplementary Figure 18). In this genetic context, we found that the ability of A375 cells to migrate is increased (in Figure 5f, compare DMSO-treated A375-miR-204 sgRNA cells with DMSO-treated A375-AVV1 sgRNA control cells) and that the anti-motility effect of vemurafenib is blunted (in Figure 5f compare vemurafenib-treated A375-miR-204 sgRNA cells with vemurafenib-treated A375-AVV1 sgRNA control cells).

The negative effects of miR-204 on melanoma cell motility were further confirmed using a different assay. As shown in Figure 5g, 5h for A375 cells, and in Supplementary Figure 17b, 17c for WM278 and SK-Mel-2 cells, we found that the over-expression of miR-204 decreases the ability of melanoma cells to invade across matrigel-coated filters in a transwell assay and potentiates vemurafenib/trametinib effects. Contrary to migration/invasion, the over-expression of miR-204 has no effect on the short- and long-term growth of A375 cells nor their ability to form colonies. Furthermore, it does not show any cooperation with vemurafenib (Supplementary Figure 19).

### miR-204 targets AP1S2

**Figure 6:**
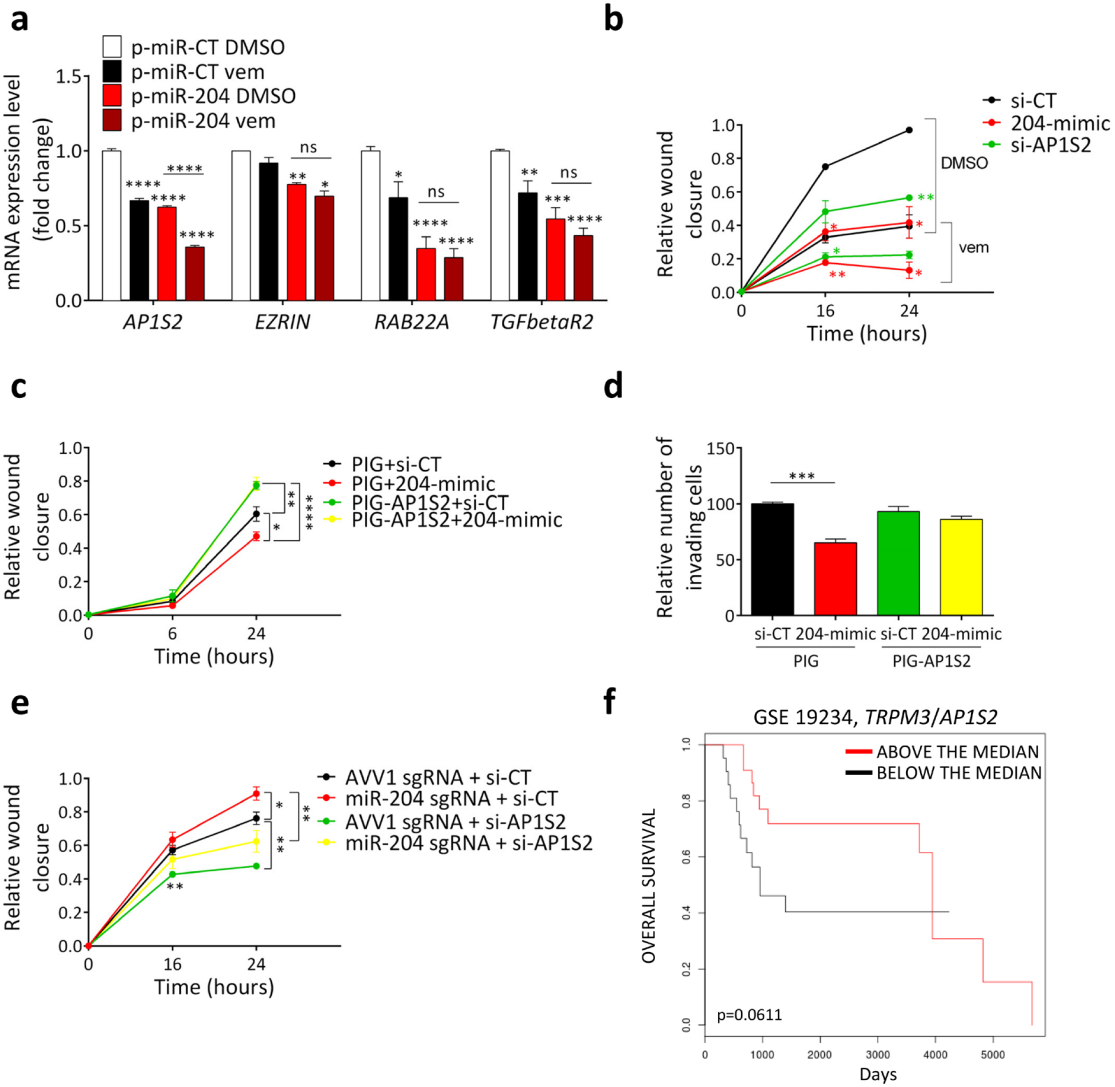
miR-204 inhibits motility by targeting AP1S2 **(a)** *AP1S2*, *EZRIN*, *RAB22A* and *TGFbetaR2* levels in A375 cells that stably over-express a control miRNA (p-miR-CT) or miR-204 (p-miR-204) and were exposed to vehicle (DMSO) or 2uM vemurafenib for 24h. **(b)** Wound closure of A375 cells transfected with si-CT (black), 204-mimic (red) or si-AP1S2 (green) and then treated for the indicated time points with vehicle (DMSO) or 2uM vemurafenib, after 24h of pretreatment with the same drug. **(c)** Wound closure of A375 cells that were stably infected with PIG empty vector (PIG) or PIG-AP1S2 and transfected with si-CT or 204-mimic. **(d)** Matrigel invasion assay performed on A375 cells that were stably infected with PIG empty vector (PIG) or PIG-AP1S2, then transfected with si-CT or 204-mimic and finally allowed to invade for 6h. **(e)** Wound closure of A375 cells that stably express a control AVV1 sgRNA or a miR-204 sgRNA and transfected with si-CT or si-AP1S2. The cells were pretreated for 72h with doxycycline in order to induce the expression of Cas9 and hence the disruption of miR-204 gene, with the consequent down-regulation of the endogenous mature miRNA levels. **(f)** Above the median (red) and below the median (black) *TRPM3*/*AP1S2* ratios allow to stratify metastatic melanoma patients according their overall survival (GSE 19234). The graphs represent the mean±SEM of 3 independent experiments. *p<0.05, **p<0.01, ***p<0.001, ****p<0.0001.

Next, we sought to determine the molecular target through which miR-204 negatively affects melanoma cell motility. We detected several validated miR-204/miR-211 pro-motility targets (Supplementary Table 7) and we found a subgroup, namely *AP1S2*, *EZRIN*, *RAB22A* and *TGFbetaR2* that exhibit a decrease in expression upon transfection of A375 cells with si-miR-204 (204-mimic) and si-miR-211 (211-mimic) (Supplementary Figure 16c and 20). We then uncovered that, when the over-expression of miR-204 is combined with vemurafenib treatment, the RNA levels of the aforementioned target genes - and especially of *AP1S2* - show a further decrease (Figure 6a). Therefore, they represent likely candidates to explain the cooperation between miR-204 and vemurafenib.

Then, we focused on AP1S2 (Adaptor Related Protein Complex 1 Sigma 2 Subunit) and we found that its knock-down by means of an siRNA (si-AP1S2) is able to phenocopy the effect of 204-mimic on migration and its cooperation with vemurafenib (Figure 6b).

Finally, we demonstrated that AP1S2 is the target through which miR-204 exerts its anti-motility activity. We established that the decrease in migration (Figure 6c) and invasion (Figure 6d) caused by miR-204 over-expression is blunted by the concomitant over-expression of the miRNA-insensitive AP1S2 open reading frame (ORF). Conversely, we showed that the increase in migration caused by miR-204 down-regulation is blunted by the concomitant knock-down of AP1S2 (Figure 6e).

Clinically, the oncosuppressive role played by miR-204 in melanoma cells through its ability to impair cell motility is further supported by the GSE 19234 dataset [41], where metastatic melanoma patients with high *TRPM3*/*AP1S2* ratio show a trend toward higher overall survival compared to those with low *TRPM3*/*AP1S2* ratio (Figure 6f and Supplementary Figure 21).

Altogether these results indicate that, in amelanotic melanoma cells, miR-204 is induced by BRAFi/MEKi and favors its anti-migratory activity by targeting *AP1S2*.

### miR-211 mediates the pro-pigmentation activity of vemurafenib in melanotic melanoma cells

We mentioned above that the cell lines where miR-211 is induced by vemurafenib (501 Mel, Sk-Mel-5 and WM-35) and trametinib (MeWo and SK-Mel-197) (Figure 3a) are melanotic. Furthermore, miR-211 has been recently reported to promote the pigmentation of mouse melanoma cells [42]. As it is already known that vemurafenib itself causes an increase in the pigmentation of melanoma cells [3], we sought to determine if miR-211 is an effector of the pro-pigmentation activity exerted by BRAFi and MEKi.

**Figure 7:**
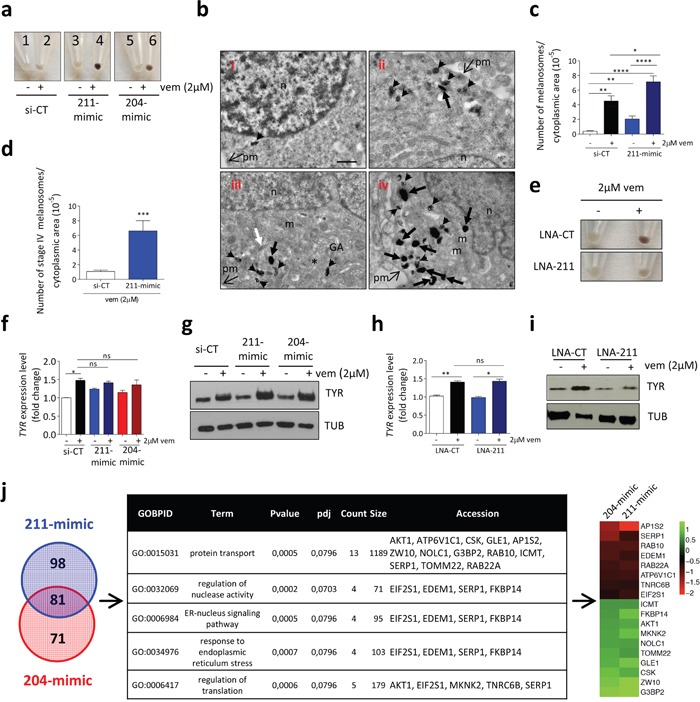
miR-211 mediates the pro-pigmentation activity of vemurafenib in melanotic melanoma cells **(a)** Melanin content of 501 Mel cells 96h after the transient transfection of si-CT, 211-mimic or 204-mimic and 72h after the treatment with vehicle (DMSO) or 2uM vemurafenib. **(b-d)** Pictures of 501 Mel cells taken by transmission electron microscopy. **(b)** i) A representative cell transfected with si-CT and treated with DMSO for 72h. ii) A representative cell transfected with si-CT and treated with 2uM vemurafenib for 72h, showing mature melanosomes. iii) A representative cell transfected with 211-mimic and treated with DMSO for 72h, showing mature melanosomes. iv) A representative cell transfected with 211-mimic and treated with 2uM vemurafenib for 72h, showing abundant mature melanosomes. Unpigmented, immature melanosomes are indicated by asterisks (stage I) and white arrows (stage II). Mature melanosomes are indicated by arrowheads (stage III) and black arrows (stage IV). n: nucleus; pm: plasma membrane; m: mitochondrion; GA: Golgi Apparatus. In all 4 panels, scale bar represents 800 nm. **(c-d)** Quantification of the total number of melanosomes **(c)** and of the number of stage IV melanosomes **(d)** upon 72h treatment with 2uM vemurafenib of 501 Mel cells transfected with si-CT or 211-mimic. **(e)** Melanin content in 501 Mel cells 96h after the transient transfection of LNA-CT or LNA-211 and 72h after the treatment with vehicle (DMSO) or 2uM vemurafenib. **(f-g)** TYR RNA **(f)** and protein levels **(g)** in 501 Mel cells 96h after the transient transfection of si-CT, 211-mimic or 204-mimic and 72h after the treatment with vehicle (DMSO) or 2uM vemurafenib. **(h-i)** TYR RNA **(h)** and protein levels **(i)** in 501 Mel cells 96h after the transient transfection of LNA-CT or LNA-211 and 72h after the treatment with vehicle (DMSO) or 2uM vemurafenib. **(j)** Schematic representation of the analysis performed on the mRNA array data. The 81 genes showing an overlap between those differentially expressed upon the transient transfection of 211-mimic vs si-CT and those differentially expressed upon the transient transfection of 204-mimic vs si-CT (*left*) were subjected to GO enrichment analysis (biological processes), which highlighted the indicated categories among the most enriched (*p<0.001, middle*). The RNAs belonging to these categories and down-regulated (red, top) or up-regulated (green, bottom) upon 204-mimic and 211-mimic transfection are listed in the heatmap (logFC, *right*). The graphs represent the mean±SEM of 3 independent experiments. *p<0.05, **p<0.01, ***<0.001, ****<0.0001.

The transient transfection of its mimic in 501 Mel cells allowed us to show that miR-211 over-expression causes an increase in pigmentation (Figure 7a, 1 vs 3 and Supplementary Figure 22a, 22b), which, according to electron microscopy, is due to an increase in the total number of melanosomes (Figure 7b, i vs iii and Figure 7c). We also observed that miR-211 over-expression strongly potentiates the pigmentation that is induced by vemurafenib (Figure 7a, 2 vs 4, Figure 7b, ii vs iv and Figure 7c). Specifically, it causes an increase in the number of heavily pigmented stage IV melanosomes (Figure 7d). This effect is not due to off-targeting, as it is reverted by the co-transfection of 211-mimic with the corresponding LNA (Supplementary Figure 22c). Furthermore, it can be observed in other melanotic cell lines treated with vemurafenib (such as SK-Mel-5, Supplementary Figure 22e), as well as in 501 Mel and SK-Mel-5 cells treated with trametinib (Supplementary Figure 22f, 22g). Conversely, when endogenous miR-211 activity is inhibited by LNA, then both the basal and the vemurafenib-induced increase in pigmentation is heavily affected (Figure 7e). These results indicate that vemurafenib does rely on miR-211 in order to exert its pro-pigmentation activity and prompted us to identify the molecular mediators.

### miR-211 targets EDEM1

In mouse melanoma cells miR-211 acts by increasing the mRNA and subsequently protein levels of Tyrosinase (Tyr) and Tyrosinase-Related Protein 1 (Tyrp1), two enzymes involved the biosynthesis of melanin pigment [42]. Therefore, we questioned whether it has a similar mode of action in human cells. Upon the treatment of 501 Mel cells with vemurafenib, we found that TYR is induced at the transcriptional level. This result is expected, given that this gene is under the transcriptional control of MITF, which is in turn activated by vemurafenib [3, 30, 43]. However, upon 211-mimic transfection there is a further increase in TYR expression that occurs only at the protein level, a result that is not in agreement with the data obtained in the mouse model (Figure 7f, 7g and Supplementary Figure 22h). Consistently, the inhibition of endogenous miR-211 by means of LNA-211 results in a decrease in TYR protein levels, while the mRNA levels remain unaltered (Figure 7h, 7i).

In order to identify the target used by miR-211 to increase TYR expression at the post-transcriptional level, we performed an mRNA array. We transfected 501 Mel cells with the control siRNA (si-CT), 211-mimic, or 204-mimic for 24h and then tested them with the Illumina ht12_V4 array. Although in melanotic cells miR-204 is not induced by vemurafenib/trametinib (see Figure 3a), its over-expression affects pigmentation in a very similar manner to that of miR-211, both at the cellular (Figure 7a, Supplementary Figure 22a, 22b, 22d) and at the molecular level (Figure 7f, 7g and Supplementary Figure 22h). Therefore, in this experiment we used it to gain stringency during the identification of the direct targets of miR-211 that can account for its pro-pigmentation activity. After confirming that the signature of differentially expressed mRNAs is consistent with miR-211 and miR-204 activity (Supplementary Figure 23), we focused on the 81 mRNAs that were differentially expressed upon the transfection of both mimics (Figure 7j, left, Supplementary Table 8, 9). In accordance with the biological effect described above, GO-enrichment analysis highlighted “protein transport/response to endoplasmic reticulum stress/regulation of translation” among the most enriched categories (Figure 7j, middle). Interestingly, among the eight down-regulated mRNAs belonging to this category, miRWalk 2.0 lists seven as predicted (*AP1S2*, *SERP1*, *RAB10*, *EDEM1*, *RAB22A*, *ATP6V1C1* and *TNRC6B*) and four as validated miR-204/211 targets (*AP1S2*, *SERP1*, *EDEM1*, *RAB22A*) (Figure 7j, right, Supplementary Table 10). These eight genes were further analyzed as described below.

**Figure 8:**
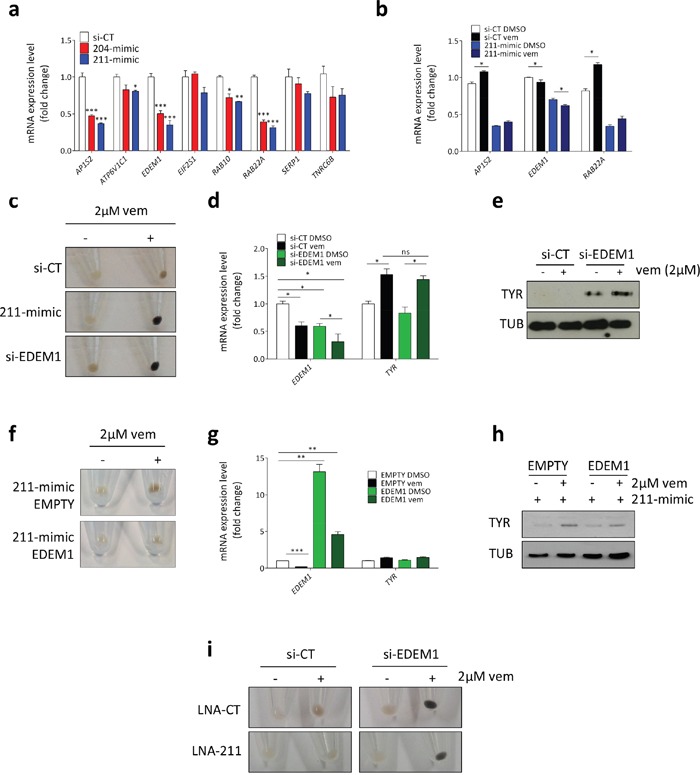
miR-211 promotes pigmentation by targeting *EDEM1* **(a)** Validation of the array results. Expression levels of the mRNAs selected through GO enrichment analysis and measured 24h after the transient transfection of si-CT (white), 204-mimic (red) or 211-mimic (blue). **(b)** Effects of the transfection with 211-mimic and the treatment with 2uM vemurafenib on the levels of the indicated mRNAs. Vemurafenib was added 24h after the transfection and cell pellets were collected after additional 48h. **(c-e)** Melanin content **(c)**, *EDEM1* and *TYR* mRNA levels **(d)** and TYR protein levels **(e)** in 501 Mel cells transfected with si-CT or si-EDEM1 and then treated with 2uM vemurafenib for 72h. **(f-h)** Melanin content **(f)**, *EDEM1* and *TYR* mRNA levels **(g)** and TYR protein levels **(h)** in 501 Mel cells stably infected with an empty lentiviral vector or a lentiviral vector expressing the miRNA-insensitive EDEM1 ORF, then transfected with si-CT or 211-mimic and finally treated with 0.5uM vemurafenib for 48h. **(i)** Melanin content in 501 Mel transiently transfected with si-CT and LNA-CT or LNA-211, or with si-EDEM1 and LNA-CT or LNA-211. 24h after the transfection the cells were treated with DMSO or 2uM vemurafenib for 72h. The graphs represent the mean±SEM of 3 independent experiments. *p<0.05, **p<0.01, ***<0.001.

Real-time PCR analysis allowed us to confirm that in 501 Mel cells 211-mimic and 204-mimic cause the down-regulation of *AP1S2*, *RAB10*, *RAB22A* and *EDEM1* (Figure 8a). Interestingly, *AP1S2*, *RAB22A* and *EDEM1* have all an established link with TYR. The link with AP1S2 and RAB22A (Ras-Related Protein 22A) is positive. Specifically, they are involved in the trafficking of ATP7A, which in turn is the transporter that provides TYR with copper (an essential cofactor for its enzymatic activity) [44]. Conversely, the link between EDEM1 (Endoplasmic Reticulum Degradation Enhancer, Mannosidase Alpha-Like 1) and TYR is negative, because EDEM1 promotes the retrotranslocation of TYR from the ER into the cytoplasm and its subsequent degradation through the ER-associated degradation (ERAD) pathway [45, 46]. Therefore, we reasoned that EDEM1 represents a good fit to explain the post-transcriptional up-regulation of TYR caused by miR-211 (miR-211 causes the down-regulation of EDEM1, hence the accumulation of TYR protein). The first indication that EDEM1 is indeed the mediator of miR-211 pro-pigmentation activity came from the observation that there is a further decrease in its expression levels when the over-expression of miR-211 is combined with the treatment with vemurafenib (Figure 8b). Furthermore, we found that si-EDEM1 is able to recapitulate the increase in melanin content (Figure 8c) and the post-transcriptional increase in TYR levels (Figure 8d, 8e) observed after the transfection of 211-mimic. Analogous results were obtained by using the chemical inhibition of EDEM1 instead of its knock-down. Kifunensine (kif), a mannosidase inhibitor known to block EDEM1 activity and favor the accumulation of TYR [47], is able to phenocopy si-EDEM1 (Supplementary Figure 24). More importantly, we performed two rescue experiments that allowed us to demonstrate that miR-211 exerts its pro-pigmentation activity by acting through EDEM1. In Figure 8f-8h we show that the concomitant over-expression of the miRNA-insensitive ORF of EDEM1 impairs the increase in pigmentation caused by 211-mimic. Conversely, in Figure 8i we show that the impairment in pigmentation that occurs when endogenous miR-211 is inhibited by the LNA is strongly reduced by the concomitant knock-down of EDEM1.

All together, these results indicate that, in melanotic melanoma cells, miR-211 is induced by BRAFi/MEKi and favors their pro-pigmentation activity by targeting EDEM1, hence promoting TYR expression and melanin accumulation. Next, we investigated whether pigmentation affects the sensitivity of melanotic melanoma cells to BRAFi/MEKi.

### Pigmentation limits the efficacy of BRAFi/MEKi

It has been recently shown that, by blocking the ERK pathway, vemurafenib disrupts the cytoplasmic production of ATP through anaerobic glycolysis. At the same time, the loss of MITF repression with the consequent induction of PGC1alpha and mitochondrial biogenesis force melanoma cells to switch back to oxidative phosphorylation. This in turn is an adaptive response that *de facto* limits vemurafenib activity by providing the cells with an alternative bioenergetic way to survive [3, 4]. On the other hand, it is known that melanosomes can sequester drugs and release them outside the cells, therefore contributing to melanoma multidrug resistance (MDR) [39, 48]. On the basis of this evidence, we hypothesized that in melanotic melanoma cells the activity of BRAFi/MEKi is limited not only by oxidative phosphorylation, but also by pigmentation.

**Figure 9:**
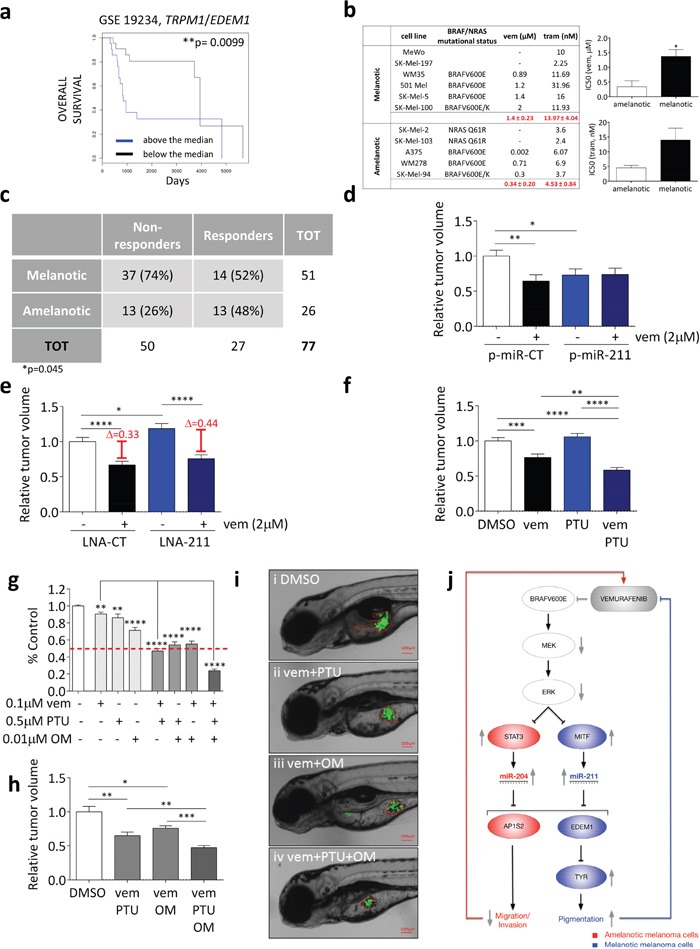
Pigmentation impairs the activity of BRAFi and MEKi **(a)** Above the median (blue) and below the median (black) *TRPM1*/*EDEM1* ratios allow to stratify metastatic melanoma patients according to their overall survival (GSE 19234). **(b)** IC50 of melanotic and amelanotic melanoma cell lines treated with vemurafenib (BRAFV600 E or K) and trametinib (BRAFV600 E or K, wt BRAF). **(c)** Response of 51 metastatic melanoma patients with melanotic tumors and 26 metastatic melanoma patients with amelanotic tumors to treatment with BRAFi or combined BRAFi/MEKi. Patients showing disease progression upon treatment were classified as non-responders, while patients showing stable disease, partial response or complete response upon treatment were classified as responders. The Fisher Exact Probability Test indicate that patients with melanotic metastases are significantly more likely to be non-responders to targeted therapy compared to patients with amelanotic metastases (p=0.045) **(d)** 501 Mel cells that stably over-express a control miRNA (p-miR-CT, white and black) or miR-211 (p-miR-211, blue and dark blue) were treated with DMSO or 2uM vemurafenib for 48h. They were then injected into the yolk sac of 48hfp zebrafish embryos. The masses of the xenografted tumors were measured 48h later. **(e)** 501 Mel cells transiently transfected with LNA-CT (white and black), or LNA-211 (blue and dark blue) were treated with DMSO or 2uM vemurafenib for 48h. They were then injected into the yolk sac of 48hpf zebrafish embryos. The masses of the xenografted tumors were measured 48h later. **(f)** 501 Mel cells were treated with DMSO (white), 0.2uM vemurafenib (black), 0.1uM PTU (blue) or 0.2uM vemurafenib plus 0.1uM PTU (dark blue) for 48h. They were then injected into the yolk sac of 48hpf stage zebrafish embryos. The masses of the xenografted tumors were measured 48h later. **(g)** Cell number upon the treatment of 501 Mel cells with 0.1uM vemurafenib, 0.5uM PTU and 0.01uM oligomycin (OM) or their combinations for one week. **(h-i)** 501 Mel cells were treated with DMSO (white), 0.1uM vemurafenib plus 0.5uM PTU, 0.1uM vemurafenib plus 0.01uM oligomycine or 0.1uM vemurafenib plus 0.5uM PTU plus 0.01uM oligomycine (dark grey) for 48h. They were then injected into the yolk sac of 48hpf zebrafish embryos. **(h)** The masses of the xenografted tumors were measured 48h later. **(i)** Representative pictures of the tumor masses. **(j)** Cartoon that summarizes the main findings of this article. miR-204 and miR-211 are negatively regulated by BRAFV600E through the ERK pathway and are under the transcriptional control of STAT3 and MITF, respectively. By targeting AP1S2, miR-204 is the mediator of the anti-motility activity exerted by vemurafenib on amelanotic cells. Conversely, by targeting EDEM1 and hence preventing TYR degradation through the ERAD pathway, miR-211 is the mediator of the pro-pigmentation activity exerted by vemurafenib on melanotic cells. Such an activity in turn limits the efficacy of vemurafenib itself. The graphs represent the mean±SEM of 3 independent experiments. *p<0.05, **p<0.01, ***<0.001, ***<0.001.

A first indication in support of our hypothesis came from the analysis of the GSE 19234 dataset of metastatic melanoma patients: similarly to what has been reported for PGC1alpha levels [3], we found that metastatic melanoma patients with high *TRPM1*/*EDEM1* ratio are characterized by lower overall survival compared to those with low *TRPM1*/*EDEM1* ratio (Figure 9a and Supplementary Figure 25-27; see also ref [49]). In ref [28], Cirenajwis and colleagues report similar results: metastatic patients with high “MITF signature” (i.e. high expression of melanocyte-associated genes) display worse prognosis.

We also found that on average melanotic cell lines display higher IC50 to vemurafenib and trametinib compared to amelanotic cell lines (Figure 9b).

Finally, although indications already exist that individual metastatic melanoma patients with melanotic tumors respond poorly to BRAFi/MEKi treatment [28], we tested a cohort composed of 77 metastatic melanoma patients [26 with amelanotic tumors and 51 with melanotic tumors], all treated with BRAFi or combined BRAFi/MEKi therapy at New York University (NYU) Langone Medical Center from 2002 to 2015 (Supplementary Table 11). Patients were divided into two groups based on their responsiveness to targeted therapy: non-responders (Progression of Disease) and responders (Stable Disease, Partial Response, and Complete Response). Analysis showed that patients with melanotic metastases were significantly more likely to be non-responders to targeted therapy compared to patients with amelanotic metastases (Figure 9c).

Next, we aimed at providing experimental evidence that pigmentation negatively affects the activity of BRAFi and MEKi. We reasoned that, if our hypothesis was correct, then the over-expression/inhibition of the pro-pigmentation miR-211 should confer melanotic cells a decreased/increased sensitivity to vemurafenib. Since both vemurafenib and miR-211 are known to exert non cell-autonomous functions [50, 51], we decided to test our hypothesis in the *in vivo* setting. 501 Mel cells that stably over-express miR-CT or miR-211 were injected into the yolk sac of zebrafish embryos and treated with vemurafenib. When we measured the mass of the xenografted tumors, we did obtain results that are in agreement with our hypothesis: miR-211 over-expression causes *per se* a decrease in cell proliferation (compare the first and the third column in Figure 9d). However, it prevents vemurafenib from working (compare the first vs the second and the third vs the fourth column in Figure 9d). Consistently, the inhibition of endogenous miR-211 causes *per se* an increase in cell proliferation (compare the first and the third column in Figure 9e), but favors vemurafenib activity (compare the first vs the second and the third vs the fourth column in Figure 9e). The effects exerted by miR-211 on proliferation and sensitivity to vemurafenib depend on its pro-pigmentation activity, as demonstrated by the fact that they are reverted by the concomitant treatment with an inhibitor of melanin biosynthesis such as N-phenylthiourea (PTU) (Supplementary Figure 28a, 28b). We also found that *in vitro* the LNA-mediated inhibition of endogenous miR-211, although ineffective *per se* (Supplementary Figure 29), cooperates with PTU in increasing the sensitivity of 501 Mel to vemurafenib (Supplementary Figure 30a). Analogous results were obtained in another melanotic cell line (SK-Mel-5, Supplementary Figure 30b).

The enhancement in the sensitivity of melanotic melanoma cells to vemurafenib by the concomitant inhibition of pigmentation was investigated further using synthetic drugs. In Figure 9f we used the graft in zebrafish embryos to show that the sensitivity of 501 Mel to vemurafenib is increased by the concomitant treatment with PTU. Finally, we assessed whether the efficacy of vemurafenib is improved even further when the adaptive responses that it elicits are both inhibited at the same time, which is to say when it is administered in combination not only with an inhibitor of pigmentation such as PTU but also with an inhibitor of oxidative phosphorylation such as oligomycin. Interestingly, the rationale for testing the combination of vemurafenib plus PTU and/or oligomycin relies also on the fact that, by targeting PDK4, miR-211 itself promotes mitochondrial biogenesis/oxidative phosphorylation as well [52]. Indeed, when we treated 501 Mel cells (Figure 9g) or SK-Mel-5 (Supplementary Figure 31a) with IC25 concentrations of vemurafenib, PTU and oligomycin, we found that both PTU and oligomycin can potentiate the activity of vemurafenib (see above and ref [3]). However, the effects are even stronger when all three of them are administered together. These results were also confirmed *in vivo* in our xenograft model (Figure 9h, 9i). Interestingly, when we performed the same experiments in the amelanotic cell line A375, we found that PTU is not effective *per se*, nor can potentiate the effects of vemurafenib and oligomycin (Supplementary Figure 31b), which further indicates that the increase in pigmentation is an additional adaptive response that limits vemurafenib effects only in melanotic cells.

## DISCUSSION

In this work, we show that in melanoma cells BRAFV600E negatively regulates miR-204 and miR-211 through the ERK pathway. We also show that, despite belonging to the same family, these two miRNAs have distinct features: i) they are under the control of different transcription factors (STAT3 for miR-204 and MITF for miR-211); ii) they are induced in different cellular contexts (miR-204 in amelanotic and miR-211 in melanotic cells); iii) they have different mechanisms of action; iv) their relationship with vemurafenib varies. Namely, by targeting AP1S2 in amelanotic cells, miR-204 mediates and potentiates the anti-motility effects of vemurafenib. Conversely, by targeting EDEM1 in melanotic cells, miR-211 mediates and potentiates the increase in pigmentation elicited by vemurafenib, which in turn represents an adaptive response that *de facto* limits its efficacy as a drug (Figure 9j)

Our study brings miR-204 to the forefront and defines the oncosuppressive role that it plays in melanoma, in spite of the fact that it is less abundant than miR-211 [17, 25–28, 53, 54]. Until now, only miR-211 had been shown to inhibit the *in vitro* and *in vivo* motility of melanoma cells, by means of its over-expression in cell lines that do not express it [18] or express it at very low levels [55]. Here, we show that miR-204 is as effective as miR-211 at inhibiting the migration/invasion of melanoma cells and, more importantly, that it exerts such an activity in the cellular contexts in which miR-211 is absent. Notably, the *TRPM3*/miR-204 locus does not undergo genomic loss like that of many classical oncosuppressors. Nonetheless, *TRPM3*/miR-204 levels are lower in nevi compared with melanoma samples [17, 25, 27]. Furthermore, we found that a high *TRPM3*/*AP1S2* ratio correlates with higher overall survival in metastatic melanoma samples (Figure 6f), a trend that reaches statistical significance when we consider the ratio between *TRPM3*/*AP1S2* and *TRPM1*/*EDEM1*, or the *TRPM3*/*TRPM1* ratio, which equals to say when we consider the subset of patients with high *TRPM3*/miR-204 and low *TRPM1*/miR-211 levels (Supplementary Figure 21).

The melanocytic lineage-specific and MITF-regulated miR-211 is invariably down-regulated and frequently lost [56–58] or epigenetically silenced [59] in melanoma samples (a decrease in its expression levels is enough to distinguish nevi from melanomas [17, 60]). Furthermore, its ability to inhibit the proliferation and, mainly, the motility of melanoma cells has been extensively demonstrated *in vitro* [18, 55, 56, 61] and in animal models (see Figure 9d, 9e and also ref [62]). Nevertheless, our study uncovers novel and highly relevant aspects of miR-211 biology, as listed below.

First, our data grant the inclusion of miR-211 in the group of miRNAs that thus far have been shown to affect pigmentation, by either promoting (miR-203 [63]) or impairing (miR-125b [64] and miR-145 [65]) its production. The protective effects exerted on TYR by miR-211 through the down-regulation of EDEM1 conceptually similar to those exerted by miR-203 through the down-regulation of KIF5B [63]. Interestingly, although miR-203 displays a much higher p-value compared to miR-204 and miR-211, it is still among the top-scoring miRNAs that are consistent with the signature of modulated mRNAs that we obtained in our mRNA array (Supplementary Figure 23b, 23c).

Second, our data help explaining the observation that the shRNA-mediated down-regulation of *TRPM1* causes a decrease in TYR protein but not mRNA levels [66]. Since the splicing of *TRPM1* pre-mRNA and the maturation of pri-miR-211 have been shown to reinforce each other [67], we can speculate that the effect observed upon *TRPM1* down-regulation is in fact due to the decrease in mature miR-211 levels. The anti-motility activity exerted by *TRPM1*/miR-211 has been itself attributed mostly to the miRNA [18].

Third, we provide a molecular mechanism explaining the increase in pigmentation that has been associated with vemurafenib and generally attributed to an increase in MITF activity [3].

The novel findings about miR-204 and miR-211 that we report in our study are important both in terms of basic miRNA biology and at the translational level.

The differences uncovered between these two miRNAs allow us to challenge the “same miRNA family = same function” rule. The molecular data shown in Figure 6a, Figure 7f, 7g and Figure 8a and the cellular assays shown in Figure 5a-5d and Figure 7a indicate that miR-204 and miR-211 target highly overlapping pools of genes and that they exert similar activities (anti-motility and pro-pigmentation), a finding that is expected from miRNAs belonging to the same family. However, we also demonstrate that it is the cellular context in which the endogenous miRNAs are induced that ultimately defines their function. Specifically, we show that miR-204 is induced in amelanotic cells, a context in which the absence of a functional melanin biosynthetic pathway makes it impossible to exert a pro-pigmentation activity. In particular, A375 cells lack the expression of TYR, the very protein whose accumulation is at the basis of the increase in melanin content. Therefore, although miR-204 in principle is able to target EDEM1 (Supplementary Figure 32), in this cellular context it can only exert its anti-motility activity. Conversely, miR-211 is induced in melanotic cells that have high expression of *MITF*/*TRPM1*/miR-211 and, consistently, limited basal migratory ability (501 Mel do not migrate in the wound healing assay; see also ref [18]). Therefore, although miR-211 is in principle able to target AP1S2 (Figure 8a), in this cellular context it can only exert its pro-pigmentation activity. In other words, the data suggest that the inability of miR-204 to increase the pigmentation of amelanotic cells, as well as the inability of miR-211 to decrease the motility of melanotic cells, is attributable to the cellular environment and not to the fact that they cannot down-regulate their relevant targets. In turn, this suggests that the final activity of a given miRNA certainly depends on the identity of its target genes and its intrinsic ability to down-regulate them [68]. However, it also depends on other factors that lay both upstream and downstream from the mere miRNA/target interactions, such as the transcription factors that regulate its expression level and the overall status of the cellular pathways to which the targets belong.

The study of miR-211 in melanotic melanoma cells has allowed us to demonstrate that it is part of the “normalization program” triggered by the inhibition of the ERK pathway: the consequent derepression of MITF promotes not only a switch from glycolysis to oxidative phosphorylation through PGC1alpha and mitochondrial biogenesis [3, 69], but also a more differentiated phenotype through *TRPM1*/miR-211 and the melanin biosynthetic pathway. We also show that this “adaptive response” limits the effectiveness of ERK pathway inhibitors and needs to be overcome by the concomitant treatment with pigmentation inhibitors in order to unleash their full potential. These findings have important therapeutic implications, as explained below.

First, they support the double role played by MITF in the melanocytic lineage. MITF is the master regulator of the melanocyte differentiation program, therefore on one side it acts as a tumor suppressor, but on the other side it allows the retreat of melanoma cells into a “functional niche” in which they are protected from vemurafenib, hence it confers drug resistance [3, 4, 70–72].

In addition, our findings allow reconciling the apparent paradox that is associated with *TRPM1*/miR-211 behavior in melanoma: as mentioned above, in the transition from nevi to primary melanoma *TRPM1*/miR-211 levels are very often down-regulated or lost [56–58]. Furthermore, in primary melanoma patients high *TRPM1* levels correlate with longer disease free survival (DFS) [73, 74]. In contrast, in metastatic melanoma, where *TRPM1*/miR-211 levels do not show a further decrease compared to patients with primary melanoma [26, 53, 57, 75], the patients associated with longer OS are those with low *TRPM1* levels (Supplementary Figure 25-27).

Finally, our findings offer a rationale for testing new combinatorial therapeutic strategies. The data reported in Figure 9 warrant to assess if the melanotic status can be considered a predictive biomarker of response to BRAFi/MEKi, as the first example in this field [76]. Going forward, an important study would be to investigate whether metastatic patients with melanotic tumors respond better to the combination of BRAFi/MEKi with pigmentation inhibitors rather than to BRAFi/MEKi alone [76]. A limiting factor might be the choice of the right pigmentation inhibitor. Currently these drugs are used only topically for the treatment of hyper-pigmentation disorders). However, they block pigmentation at different levels [77] and there might be some that are compatible with systemic administration. In addition, our findings warrant studies examining whether the efficacy of BRAFi/MEKi on melanotic melanoma patients is further increased when not only pigmentation inhibitors, but also inhibitors of oxidative phosphorylation/mitochondrial biogenesis are added to the treatment regimen. It is important to keep in mind that all such testing should be performed on therapy-naïve patients that receive their first line of treatment. The reactivation of the ERK pathway that occurs in the vast majority of relapsing tumors brings PGC1alpha and *TRPM1*/miR-211 back to basal levels and prevents their induction upon BRAFi/MEKi treatment ([3] and Figure 3). Therefore, it blunts the adaptive responses that are at the basis of the cooperation between BRAFi/MEKi themselves and the inhibitors of pigmentation/mitochondrial functioning.

In conclusion, our work highlights the importance of the study of the non-protein coding genes that are regulated by BRAFV600E through the ERK pathway, since they can contribute to a deeper understanding of BRAFV600E biology and of the functioning of BRAF inhibitors. This in turn can lead to novel strategies for the improvement of the still suboptimal therapeutic options available for metastatic melanoma patients [9].

## MATERIALS AND METHODS

Cell culturing, as well as cellular assays (transient transfections, stable infections, growth curve, cell cycle analysis, clonogenicity assay, soft agar assay, limiting dilution assay, co-colture assay, migration assay, invasion assay, melanin content evaluation) and molecular assays (PCR, real-time PCR, western blot) were performed according to standard procedures, which are described in details in Supplementary Materials and Methods. *In vivo* experiments were performed in compliance of protocols approved by the Italian Ministry of Health. Details on the experimental procedures followed are reported in Supplementary Materials and Methods. The same file contains details on transmission electron microscope analysis, on the execution and the analysis of miRNA-seq and mRNA array, as well as on correlation analysis, seed enrichment analysis, miRNA target enrichment analysis and on the analysis of metastatic melanoma sample datasets.

## SUPPLEMENTARY MATERIALS FIGURES AND TABLES












