# Supplementary material for: Context-dependent miR-204 and miR-211 affect the biological properties of amelanotic and melanotic melanoma cells
Source: Oncotarget. 2017 Mar 6;8(15):25395–417. doi: 10.18632/oncotarget.15915 (PMC5421939; doi:10.18632/oncotarget.15915)
Supplement: Supplementary file 1 [file oncotarget-08-25395-s001.pdf]

Context-dependent miR-204 and miR-211 affect the biological properties of amelanotic and melanotic melanoma cells

SUPPLEMENTARY DATA

Cell culture

Cells were grown at 37°C in a humidified atmosphere with 5% CO_2. A375, 501 Mel, SK-Mel-5, SK-Mel-28, WM266-4, MeWo, SK-Mel-2 and SK-Mel-197 melanoma cells lines were cultured in DMEM supplemented with 10% foetal bovine serum, 1% glutamine (Sigma-Aldrich) and 1% penicillin/streptomycin (Euroclone). WM35 and WM278 were grown in 4/5 MCDB 153 Media (Sigma-Aldrich), 1/5 Leibovitz L-15 Media (Euroclone), 2% Fetal Bovine Serum (Euroclone), 5ug/uL Insulin (Sigma-Aldrich), 15 ug/uL Bovine Pituitary Extract (Millipore), 1.68mmol/L $CaCl_2$ (Sigma-Aldrich), 50ug/mL Epidermal Growth Factor (BD Biosciences). The identity of each cell line is confirmed by fingerprinting, as reported in [1].

Generation of vemurafenib-resistant clones and populations

Clones

A375 and SK-Mel-28 cells were plated at low density ($1.5x10^4$) on 10cm dishes and 24h later they were treated with 2uM vemurafenib. Once clones became visible, one from each plate was isolated, expanded and analyzed by molecular and cellular assays.

Populations

A375, 501 Mel and SK-Mel-28 cells were plated at low density ($5x10^4$) on 10cm dishes and 24h later they were treated with 2uM vemurafenib. Once the cells gained the ability to grow in the presence of vemurafenib (which happened about one month from the beginning of the treatment), they were expanded and analyzed by molecular and cellular assays.

Drugs

AZD1480 (S2162), S3I-201 (501919-59-1), trametinib (GSK1120212, S267304), vemurafenib (PLX-4032, 1029872-54-5) and WP1066 (857064-38-1) were purchased from Selleckchem. Dacarbazine (D2390), doxycycline hyclate (D9891), kifunensine (K1140) and oligomycin A (75351) were purchased from Sigma-Aldrich. PP1 (P-6420) and wortmannin (W-2990) were purchased from LC Laboratories. N-Phenylthiourea (P7629) was purchased from Aldrich. All drugs were diluted according to the manufacturer's instructions.

Oligos

PCR and qRT-PCR oligos were purchased from Primm srl; LNA inhibitors were purchased from Exiqon; siRNAs for mRNA knock-down or microRNA mimicking (mimics) were purchased from Shanghai GenePharma. For sequences, see Supplementary Table 2, 7, 12 and 13.

Plasmids

pTRE-TIGHT-BI-RY SCR/miR-204 sensor

One copy of a miR-204 complementary sequence and a scrambled sequence were cloned into the pTRE-TIGHT-BI-RY-0 plasmid (#31463, Addgene), adding the NheI restriction site as analytic control. In details, sense and antisense oligos (SCR sensor sense 5'-TCGACGCTA GCTTCCCTTTGTCATCCTATGCCTA-3' and antisense 5'-AGCTTAGGCATAGGATGACAAAGGGAAGCTAG CG-3'; miR-204 sensor sense 5'- TCGACGCTAGCT GGGCGTATAGACGTGTTACACA-3' and antisense 5'-AGCTTGTGTAACACGTCTATACGCCCAGCTAG CG-3') were annealed to make a double strand fragment with sticky ends (HindIII/SalI). The double strand was then phosphorylated using PNK enzyme (New England BioLabs), according to the manufacturer's instructions, and finally cloned into the pTRE-TIGHT-BI-RY-0 plasmid, previously digested with HindIII and SalI.

pCW-rtTA

This plasmid was obtained from the pCW-Cas9 plasmid (see below), by removing the Cas9 coding sequence. This was done using a digestion with XhoI and BamHI and the subsequent fill-in with Klenow enzyme (New England BioLabs).

PIG-BRAFV600E-Δ[3–10]

BRAFV600E Δ [3–10] coding sequence was amplified by PCR (Phusion Flash High-Fidelity PCR Master Mix, Thermo Scientific) from A375 cDNA, using the following primers: F 5'-CCCGGCTCTCGGTTATAAGA-3'and R 5'-AC AGGAAACGCACCATATCC-3'. It was then cloned into pGEM®-T Easy Vector (Promega) according to the manufacturer's instructions. Subsequently, it was amplified again by PCR using the following primers: F 5'-CATGTCGACGGCTCTCGGTTATAAGATGGC-3' and R 5'-GCAGTTAACTCAGTGGACAGGAAACG CACCATATC-3'. The PCR product was first digested

with SalI-HpaI enzymes and then cloned into XhoI-HpaI digested pMSCV-PIG plasmid (PIG, kind gift from Dr. Pandolfi, BIDMC-HMS).

PIG-AP1S2

AP1S2 coding sequence was amplified by PCR (Phusion Flash High-Fidelity PCR Master Mix, Thermo Scientific) from A375 cDNA, using the following primers: F 5'-CATGCGGCCGCCACCATGCAGTTTATGTTGC-3' and R 5'-ATGCTCGAGTTATGTCAGTCCAATTTCTTC-3'. The PCR product was then cloned into PIG-NotI plasmid (a modified version of PIG plasmid in which a NotI restriction site is added to the multicloning site) using NotI and XhoI as restriction enzymes.

As negative controls for PIG-BRAFV600E-Δ[3–10] and PIG-AP1S2 plasmids, the corresponding empty PIG plasmids were used.

pGIPZ-miR-204 (p-miR-204) and pGIPZ-miR-211 (p-miR-211)

miR-204 and miR-211 pri-miRNAs were amplified from A375 genomic DNA using the following primers: F 5'-CATCTCGAGGACAGGGTGATGGAAAGGAG–3' and R 5'–GCAACGCGTGCATTTGATGATGGTGCAAT–3' (miR-204); F 5'–CATCTCGAGTGATGCTGCAGAGTGGGTAG–3' and R 5'-GCAACGCGTATATCCTGGACTCCGCCTTT-3' (miR-211). Then, both pri-miRNAs were cloned instead of the control miRNA into the pGIPZ-miR-CT plasmid (kind gift from Dr. Hernando, NYU), using XhoI and MluI as restriction enzymes. See also Supplementary Figure 16.

pWPXLd-EDEM1

EDEM1 coding sequence was amplified by PCR (Phusion Flash High-Fidelity PCR Master Mix, Thermo Scientific) from p3XFLAG-CMV-14-EDEM1 (kind gift from Dr. Hebert University of Massachusetts), using the following primers: F 5'-CATACGCGTCCCCGCGCTTTAAAATAATG-3' and R 5'-TGACTAGTCACCCGGGATCACTACTTGT-3'. It was then cloned into pWPXLd lentiviral vector (kind gift from Dr. Cremisi, Scuola Normale Superiore), using MluI and SpeI as restriction enzymes. The pWPXLd vector deprived of GFP (using MluI and SmaI restriction sites) was used as negative control.

pCW-Cas9

This plasmid was purchased from Addgene (#50661).

pLX-miR-204-sgRNA

The sgRNA for miR-204 was generated by PCR (Phusion Flash High-Fidelity PCR Master Mix, Thermo Scientific) using the pLX-AVV1-sgRNA plasmid as template (#50662, Addgene) and following the protocol available at www.addgene.org/. The primers used were: F1 5'-AAACTCGAGTGTACAAAAAAGCAGGCTTTAAAG-3', R1 5'-CCTTCATATATTCTCAGGCACGGTGTTTCGTCCTTTCC-3', F2 5'-GTGCCTGAGAATATATGAAGGGTTTTAGAGCTAGAAATAGCAA-3', R2 5'-AAAGCTAGCTAATGCCAACTTTGTACAAGAAAGCTG-3'. The PCR product was then cloned instead of the control sgRNA into the pLX-AVV1-sgRNA plasmid (#50662, Addgene), using XhoI and NheI restriction sites.

Transfection of siRNAs, miRNA mimics and LNAs

2 to $3x10^5$ cells were seeded in 6well plates in order to reach 80%-90% confluency the day after. Seeded cells were transfected with 10ul Lipofectamine 2000 (Life Technologies) and 60 nM siRNA, miRNA mimic or LNA in Optimem (Life Technologies). 6h post-transfection, the medium was changed to complete medium and the cells processed according to the protocol of the different assays.

Stable infections

The stable infection of retroviral plasmids such as PIG-BRAFV600E-Δ [3–10] and PIG-AP1S2 was carried out as described in [2]. The stable infection of lentiviral plasmids such as p-miR-204, p-miR-211, pWPXLd-EDEM1, pCW-Cas9, pLX-AVV1-sgRNA, pLX-miR-204-sgRNA, pGIPZ-tGFP and pGIPZ-mCherry was carried out as described in [3].

Generation of A375 cells that stably express the SCR and the miR-204 sensor

The pTRE-TIGHT-BI-RY SCR sensor plasmid or the pTRE-TIGHT-BI-RY miR-204 sensor plasmid was co-transfected with the pCW-rtTA plasmid in A375 cells, using Lipofectamine 2000 (Thermo Scientific). The molar ratio between the SCR/miR-204 sensor and the pCW-rtTA plasmid was 5:1. Transfected cells were then selected using 2ug/ml puromycine. The puromycine-resistant population, which expresses both plasmids, was later enriched in mCherry and eYFP double positive cells, using fluorescence activated cell sorting (FACSjazz, BD) after 48h of induction with 2 ug/ml doxycycline.

Growth curve assay with different doses of drug

$3x10^3$ melanoma cells were seeded in 12well plates (3 wells per experimental condition per time point) and 24h later they were treated with different doses of the appropriate drug or with vehicle (DMSO) for about a week. Cells were then fixed with 4% PFA and stained with a crystal violet solution (0.1% crystal violet, 20% methanol, in water). After the excess crystal violet solution

was removed and the plates were washed with tap water and dried, cells were de-stained using a 10% acetic acid solution. Absorbance was then read at 590nm. Each sample was normalized on the vehicle-treated sample and the data were graphed as variation of cell percentage compared to the vehicle-treated sample.

Growth curve assay at different time points

$3x10^3$ cells were seeded in 12well plates (3 wells per experimental condition per time point) and 24h later they were treated with the appropriate dose of drug or with vehicle (DMSO). Each time point was then fixed with 4% PFA and stained using a crystal violet solution (0.1% crystal violet, 20% methanol, in water). After the excess crystal violet solution was removed and the plates were washed with tap water and dried, cells were de-stained using a 10% acetic acid solution. Absorbance was then read at 590nm. Each sample was normalized on the time 0 sample and the data were graphed as cell percentage compared to time 0.

Cell cycle analysis

$3x10^5$ cells were seeded in 100mm dishes and the day after were treated with either 2uM vemurafenib or vehicle (DMSO). After 48h, cells were harvested with trypsin, then 10^5 cells were fixed with 95% ethanol and stained with propidium iodide solution (200ug/ml P.I., 0.1% NaCitrate, 0.5 mg/ml RNAse A, 0.1% Nonidet NP40). For each sample, 10^4 events were analyzed by flowcytometry (C6 Accuri, BD).

Clonogenicity assay

Cells were seeded ($2x10^2$) in 60mm plates in triplicate and treated with vehicle (DMSO) or different doses of vemurafenib. After 8 days, cells were fixed and stained with a 0.1% crystal violet, 4% formaldehyde solution. The number of colonies of the vemurafenib-treated samples was normalized on the number of colonies obtained with the vehicle.

Soft agar assay

10^4 cells were resuspended in media plus 0.3% agarose, treated with either DMSO or 2uM vemurafenib and plated on an agarose base (DMEM plus 0.6% agarose, prepared the day before) in a well of a 6well plate. Each experimental condition was plated in triplicate. After 10-15 days, the colonies that were visible in five randomly chosen microscope fields were counted using a 10X objective. Data were normalized on the number of colonies counted in the vehicle-treated wells.

Limiting dilution assay

Cells were seeded in a 96well plate at 3 cells/well concentration (32 wells), 1 cell/well concentration (32 wells) and 0.3 cell/well concentration (32 wells). Two weeks later, the wells containing colonies were counted as “1”, while those bearing no colonies were counted as “0”. The cells grown in the “1” wells were tripsinized and mixed all together. They were then counted and seeded again following the same protocol (3, 1 and 0.3 cells/well). After two weeks, a new round of counting and seeding was performed.

Co-colture assay

A375 parental cells were stably infected with either pGIPZ-tGFP or pGIPZ-mCherry lentiviral vectors (kind gifts from Dr. Hernando, NYU), while A375 C2 vemurafenib-resistant clone was stably infected with pGIPZ-tGFP lentiviral vector. $4x10^5$ cells were seeded in 10mm plates and treated with 2uM vemurafenib or with the vehicle (DMSO). Red A375 cells were seeded in a 3:1 ratio together with green cells, either A375 or A375 C2. Fluorescence percentage of each cell mixture was evaluated every 3-4 days by flowcytometry (C6 Accuri, BD) and $4x10^5$ cells were seeded again. For each sample, 10^4 events were analyzed.

Migration assay

About $1.5x10^4$ cells were seeded on a plate using silicone inserts (IBIDI) and were incubated in complete medium containing 2uM vemurafenib or DMSO. 24h later (t0), when the seeded cells reached ~70-80% confluence, the inserts were removed and the quality of the covered surface was evaluated. Then, the cell-free gaps were monitored at different time points after insert removal, using 10x and 20x objective lens. Images were captured using Leica DM IL LED microscope. The measurement of cell-free gaps was taken with Image J software (http://rsb.info.nih.gov). The migratory rate was determined as percentage of gap closure compared to the t0 area.

Invasion assay

Invasiveness of melanoma cells was determined using polycarbonate filters (8um pore size, 6.5mm diameter), coated with 12.5ug matrigel (BD Biosciences)/filter and mounted in Boyden’s chambers. $1x10^5$ cells resuspended in 200uL of their own growth medium were seeded in the upper compartment, while fresh complete medium was added in the lower chamber as chemo attractant. Cells were incubated for 6 or 24h at 37°C, 5% CO2. After incubation, filters were removed and the non invading cells on the upper surface were wiped-off mechanically with a cotton swab,

while invasive cells adherent on the lower filter surface were fixed overnight in ice-cold methanol, and stained using a Diff-Quick kit (BD Biosciences). For each condition, 3 filters were used. Invasive cells were counted on pictures of randomly chosen fields.

Melanin content evaluation and quantification

$3x10^5$ melanotic melanoma cells were seeded in 60mm plates and 24h later they were treated with either DMSO or drug (vemurafenib or trametinib). After 72h, the cells were harvested and counted. Finally, pictures were taken on equal numbers of pelleted cells.

For melanin quantification, cell pellets were resuspended in 300ul NaOH (1M) and incubated for 3h at 85°C on a shaker plate. The mixture was then spinned down and the absorbance of the supernatant was measured at 470nm.

Transmission electron microscope analysis

To evaluate their morphological features, 501 Mel cells were seeded at $2x10^5$ cells/P30. The day after they were transfected with 60nM si-CT or 211-mimic. After 6h, they were trypsinized and divided in 2 wells of a 6well plate. The day after they were treated with either DMSO or 2uM vemurafenib for 72h. At the end of this period, cells were harvested and pelleted by centrifugation. Pellets were washed three times with phosphate buffered saline (PBS) solution and fixed in 2.5% glutaraldehyde solution in 0.1M cacodylate buffer, pH 7.2, for 2h at 4°C. Cells were then scraped off and post-fixed in 1% osmium tetroxide in 0.1M cacodylate buffer for 2h at room temperature. After rapid dehydration in a graded series of ethanol and propylene oxide, cells were embedded in an "Epon-Araldite" mixture. Ultrathin sections, obtained by a diamond knife on an Ultracut Reichert-Jung ultramicrotome, were placed on Formvar-carbon coated nickel grids, stained with uranyl acetate and lead citrate and observed with a Jeol 100 SX transmission electron microscope. The quantification of the number of melanosomes per cell was performed by counting the number of melanosomes per unit of cytoplasmic area.

Genomic DNA extraction

$3x10^6$ cells were resuspended in 500ul of Lysis Buffer (10mM Tris-HCl; 400mM NaCl; 2mM EDTA; pH 8) supplemented with 40ul of 10% SDS and 20ul of RNAse 1mg/ml. The mixture was then incubated at 37°C for 1h, spinned down and mixed with 10ul of proteinase K 18. mg/ml (Sigma). Subsequently, it was incubated at 50°C for 1h, spinned down and mixed with 200ul of saturated NaCl. The sample was then centrifuged at 12000rpm for 10 minutes. The supernatant was collected, mixed with 2 volumes of 100% ethanol and finally centrifuged for 5 minutes at 12000rpm. The obtained pellet was washed with 70% ethanol and resuspended with 80ul of TE buffer.

Fingerprinting

Cell lines fingerprinting was performed by using the AmpFiSTR Identifiler PCR Amplification kit (Applied Biosystems), according to the manufacturer's instructions. Data were analyzed as described in [1].

RNA extraction and quantification

For general purposes and for the mRNA array, RNA was extracted using QIAzol reagent (Qiagen), following the manufacturer's instructions. For microRNA sequencing, RNA was extracted using miRNEasy MINI Kit (Qiagen), following the manufacturer's instructions. RNA was subsequently quantified using Nanodrop Lite (Thermo Scientific).

DNAse treatment and retrotranscription

When analyzing mRNA expression, 1ug of RNA was treated with DNAse I, amplification grade (Invitrogen) following the manufacturer's instructions. 500ng of DNAse-treated RNA were then retrotranscribed with iScript cDNA Synthesis Kit (Bio-Rad) using a S1000 Thermal Cycler (Bio-Rad).

Genomic contamination of RNA was ruled out by performing a PCR reaction on the cDNA using PCR Master Mix (Thermo scientific) and the ATPA1 primers (Supplementary Table 12). These primers produce a genomic-derived amplicon of 300bp and a cDNA-derived amplicon of 180bp, allowing for genomic DNA contamination detection.

When analyzing miRNA expression, the DNAse treatment was skipped and 250ng of RNA were retrotranscribed using miScript II RT Kit (Qiagen).

Real-time PCR

Quantitative real-time PCR (qRT-PCR) was performed with SsoAdvanced Universal Supermix (Bio-Rad) on a CFX96 Real-Time System (Bio-Rad). A melting curve was performed after each PCR reaction to confirm the specificity of the primers. All reactions were performed in duplicate. Data were analyzed using CFX Manager Software (Biorad).

Protein extraction

Melanoma cells (10^6) were resuspended in 70ul of lysis buffer (Tris HCl 50mM, 1% TritonX100,

0.25% of NaDeoxicholate, PMSF 1mM, Orthovanadate 2mM, proteinase inhibitors cocktail). The mixture was incubated for 30 minutes on ice and then centrifuged at 14000rpm for 30 minutes at 4°C. The supernatant was then quantified using Bradford reagent and read at 590nm.

Western blot

The following antibodies were purchased from Cell Signaling and diluted according to the manufacturer's instructions: anti-MEK 1/2 (4694), anti-phospho-MEK 1/2 (9154), anti-STAT3α (8719), anti-phospho-STAT3 (9145). Anti-BRAFV600E (E19290) was purchased from Spring Bioscience and diluted according to the manufacturer's instructions. Anti-Tyrosinase (sc-20035) and anti-phospho-ERK (sc-7383) were purchased from Santa Cruz and diluted according to the manufacturer's instructions. Anti-α-Tubulin (T9026) was purchased from Sigma-Aldrich and diluted according to the manufacturer's instructions.

Xenograft in zebrafish embryos

p-miR-CT or p-miR-211 501 Mel cells were seeded at $4x10^5$/100mm plate (1 plate per experimental condition) and 24h later they were treated with the appropriate drug or with vehicle (DMSO) for 48h. Cells were then harvested and used to make a mix of $5x10^5$ cells in 2ul of matrigel (Cultrex Basement Membrane Extract, PathClear). Cell suspension was loaded into a borosilicate glass capillary and injected (250 cells/nl) into the perivitelline space of 48hpf zebrafish embryos of the Tg(myil7:DsRed) strain (kindly provided by Dr. Didier Stainier, University of California), using a microinjector (Tritech Research). Embryos were previously dechorionated manually or using Pronase (Boehringer Mannheim) and anesthetizes with 0.04 mg tricaine (Sigma-Aldrich). At least 30 embryos were injected per experimental condition and each experiment was repeated three times. Fluorescence imaging was carried out 2 days after the injection, using the Nikon Eclips E600 microscope. Acquisitions were performed using the CoolSnap-CF camera and NIS-Elements software version 2.0. Tumor areas were analyzed using ImageJ software (http://rsb.info.nih.gov).

Statistical analyses

Data were analyzed with unpaired t test (GraphPad Prism, GraphPad Software Inc.). Values of $p<0.05$ were considered statistically significant (*p<0.05, **p<0.01, ***p<0.001, ****p<0.0001). The mean±SEM of three independent experiments is reported.

RNA sequencingof small RNAs (miRNA-seq)

Sample preparation

10^6 A375 and A375 C2 cells were seeded in 10cm dishes. The following day, DMSO or 2uM vemurafenib were added for 48h. Total RNA was then extracted using miRNeasy mini kit (Qiagen). In order to account for biological variability, 2 independent replicates of this experiment were subjected to sequencing.

Library generation and sequencing

Small RNA libraries were prepared starting from 1 ug of total RNA with RIN = 10 (Bioanalyzer, Agilent). The TruSeq RNA Sample Preparation Kit (Illumina) was used, following the manufacturer's suggestions.

Small RNA libraries were loaded at the 6-plex level of multiplexing (approximately 20 million reads per sample) into a V3 flow cell and they were sequenced in single-read mode (50bp) on a HiSeq2000 (Illumina, San Diego, CA, USA).

Both the library preparation and the sequencing were performed by IGA Technology Services, Udine, Italy.

Pre-filtering, trimming and mapping

Sequencing data were analysed as previously described [4]. In more details, raw sequences were de-multiplexed using the Illumina pipeline CASAVA v1.8. FastQC v0.10.1 (http://www.bioinformatics.babraham.ac.uk/projects/fastqc/) was used for quality check, while Cutadapt v.1.2.1 (http://code.google.com/p/cutadapt/) was used for trimming adapter sequences off the raw reads. Reads with N calls were discarded using FASTX_Toolkit (0.0.13.1, http://hannonlab.cshl.edu/fastx_toolkit/) and only reads with all base calls showing a Phred score higher than 30 were retained for further analysis. These high quality reads, which resulted to have a length ranging between 17 and 35bp after trimming, were clustered for unique hits and mapped to pre-microRNA sequences (miRBase v.20) employing miRExpress (v2.1.3) [5]. We allowed a minimum of 95% of sequence identity between the read and the reference and a length tolerance range of 4bp for mapping. On average, 23 millions of reads were produced per sample and, after trimming, around 19 millions of reads were used for microRNAs identification. 1243 different microRNAs were identified on average in each sample. Detailed statistics are available in Supplementary Figure 5a.

Clustering and differential expression analysis

Count-based microRNA expression profiles were analysed using Bioconductor's package DESeq [6]. Read count matrices for each sample were normalized by library size factors to a common scale. Variance stabilizing transformed (VST) count data was exploited to study

the relationship between samples. For the hierarchical clustering, we used the Euclidean as distance metric and the complete as agglomeration method. For the differential expression analysis, we followed the DESeq approach using a negative binomial distribution model and local regression to estimate the relationship between the dispersion and the mean of each microRNA. In this way *p*-values were estimated and adjusted for multiple testing by the Benjamini and Hochberg procedure [7], controlling the false discovery rate (FDR).

We considered differentially expressed only those microRNAs with a fold change (FC) higher than 2 or lower than 0.5 and with an adjusted *p*-value0.05. In so doing, we found that 148 microRNAs are significantly modulated in A375 treated with vemurafenib compared to DMSO (FC(A375 vemurafenib vs A375 DMSO) >2 or <0.5, padj<0.05, Supplementary Table 3) and 102 microRNAs are significantly modulated between A375 and A375 C2 treated with vemurafenib (FC(A375 vemurafenib vs A375 C2 vemurafenib) >2 or <0.5, padj<0.05, Supplementary Table 4). For further analyses, we selected the 72 microRNAs that are common to both groups and show the same trend (concordant sign in the logFC). Then, by focusing on those that are not significantly altered in A375 C2 by vemurafenib treatment (0.5< FC(A375 C2 vemurafenib vs A375 C2 DMSO) < 2) and by applying a cutoff of 100 reads in at least one condition, we ended up with the 53 microRNAs that are listed in Supplementary Table 5 and shown in Figure 2c.

The small RNA sequencing data have been deposited in NCBI's Gene Expression Omnibus [8] and are accessible through GEO Super Series accession number GSE 94477.

mRNA array

Sample preparation

$1.5x10^6$ 501 Mel cells were seeded in 10cm dishes. The following day, they were transfected with si-CT, 204-mimic or 211-mimic (60nM) using lipofectamine 2000 (Thermo Scientific). Total RNA was subjected to HumanHT-12v4 array (Illumina). In order to account for biological variability, two independent replicates of this experiment were subjected to analysis.

Bioinformatics analysis of mRNA expression arrays

The arrays HumanHT-12v4 arrays were scanned on a BeadScan station, and analysed using R (v 2.15.2) and various Bioconductor packages. Raw data were normalized using the 'lumi' package (v 2.10) [9], by applying variance-stabilizing transformation [10] followed by robust spline normalization [11]. Probes with detected expression above background (detection p-value<0.01) in all samples were retained. In order to reduce the number of false positives, the unannotated probes were excluded after retrieving the nuID of each of them from the *lumiHumanAll* annotation database (v 1.18) [12], using the 'annotate' package (v 1.36). Linear modelling was fitted for each gene (n=22111) and moderated t-statistics were computed using 'limma' package (v 3.14.4) [13] to test for differential expressed genes (DEG). Resulting p-values were adjusted using the Benjamini & Hochberg method [7]. mRNAs that resulted differentially expressed (|log2FC|>1.5 and padj0.05) upon the transfection of both 204-mimic and 211-mimic (n=81, Supplementary Table 8,9) were searched for enrichment in their Gene Ontology annotation using GOstats (v 2.24).

The mRNA array data have been deposited in NCBI's Gene Expression Omnibus [8] and are accessible through GEO Super Series accession number GSE 94477.

Correlation analysis

The expression levels of *TRPM1*, *TRPM3* and *MITF* in cutaneous melanoma samples were retrieved from the microarray data published in ref [14] (GSE 3189), ref [15] (GSE 7553), ref [16] (GSE 12391) and ref [17] GSE 65904, as well as from www.cbioportal.org. In the case of multiple probes for the same transcript (Supplementary Table 6), the average was calculated. Log2 transformed values (GSE 3189, GSE 7553, GSE 65904 and www.cbioportal.org) and log10 (test/ref) ratios (GSE 12391) were then used to calculate the Pearson correlation coefficient (R), taking advantage of GraphPad prism software.

Seed enrichment analysis

This analysis was performed along the lines of what reported in [18]. First, the FASTA format of the 3' UTR sequences of the transcripts present in the array and passing quality filters were obtained from Ensembl release 80 using Bioconductor biomaRt package (v. 2.14.0) [19, 20]. 3'UTRs were filtered to remove redundancy due to different transcript variants and the longest UTRs were retained, yielding a final set of 13521 sequences (background set). Then, the presence of canonical seed matches for the miR-204/miR-211 seed (AAGGGA, AAAGGGA, and CAAAGGGA) was established for both the background set, as well as for the subset of 3'UTRs that, according to the results of the mRNA array, belong to mRNAs that are down-regulated or up-regulated upon miR-204 or miR-211 over-expression (see Supplementary Table 8 and 9, respectively). The canonical seed match mapping was performed using the PITA algorithm, which does not allow mismatches, loops and G:U wobbles (http://genie.weizmann.ac.il/pubs/mir07/mir07_exe.html). Finally, the frequency of seed matches present in the subset of RNAs that are down-regulated or up-regulated

by each of the 2 microRNAs was compared to the frequency of seed matches present in the background set, using one-sided Fisher's exact test in the R environment. The results of such analysis are reported in Supplementary Figure 23a.

miRNA target enrichment analysis (miTEA)

miTEA (miRNA target enrichment analysis [21]) is a computational approach that allows to infer miRNA activity from the results of mRNA arrays. Starting from the list of transcripts modulated upon the over-expression of a given microRNA and ranked on the basis of their p value and logFC, this method allows to predict which is the microRNA that, according to TargetScan target prediction algorithm, could most likely be responsible for such a modulation. TargetScan is chosen as target prediction algorithm because it was shown to be the most suitable for this application. The miTEA was applied to the data obtained by mRNA array and the results are reported in Supplementary Figure 23b, 23c.

Analysis of metastatic melanoma sample datasets

GSE 19234 (n=44) and GSE 65904 (n=214) melanoma datasets were downloaded from Gene Expression Omnibus GEO (www.ncbi.nlm.nih.gov/geo). The best matching probes for the genes of interest in the GSE 19234 dataset were retrieved using the JETSET SCORE (http://www.cbs.dtu.dk/biotools/jetset/) for the Affymetrix HG U133 PLUS V2 chip. For the TCGA-skin cutaneous melanoma dataset (n=472), z-scores for genes of interest were obtained from the cBioportal site (http://www.cbioportal.org/index.do). For the three datasets, clinical information was available to carry out survival analysis. Kaplan-Meier curves were generated for each dataset after dividing samples into two groups according to the median expression value of the selected genes/gene ratios/gene combinations.

REFERENCES

1. Marranci A, Tuccoli A, Vitiello M, Mercoledi E, Sarti S, Lubrano S, Evangelista M, Fogli A, Valdes C, Russo F, Monte MD, M AC, Pellegrini M, Capobianco E, Tsinoremas N, Poliseno L. Identification of BRAF 3'UTR Isoforms in Melanoma. J Invest Dermatol. 2015; 135:1694-1697.
2. Poliseno L, Salmena L, Riccardi L, Fornari A, Song MS, Hobbs RM, Sportoletti P, Varmeh S, Egia A, Fedele G, Rameh L, Loda M, Pandolfi PP. Identification of the miR-106b~25 microRNA cluster as a proto-oncogenic PTEN-targeting intron that cooperates with its host gene MCM7 in transformation. Sci Signal. 2010; 3:ra29.
3. Gaziel-Sovran A, Segura MF, Di Micco R, Collins MK, Hanniford D, Vega-Saenz de Miera E, Rakus JF, Dankert JF, Shang S, Kerbel RS, Bhardwaj N, Shao Y, Darvishian F, Zavadil J, Erlebacher A, Mahal LK, et al. miR-30b/30d regulation of GalNAc transferases enhances invasion and immunosuppression during metastasis. Cancer Cell. 2011; 20:104-118.
4. Barsanti C, Trivella MG, D'Aurizio R, El Baroudi M, Baumgart M, Groth M, Caruso R, Verde A, Botta L, Cozzi L, Pitto L. Differential regulation of microRNAs in end-stage failing hearts is associated with left ventricular assist device unloading. Biomed Res Int. 2015; 2015:592512.
5. Wang WC, Lin FM, Chang WC, Lin KY, Huang HD, Lin NS. miRExpress: analyzing high-throughput sequencing data for profiling microRNA expression. BMC Bioinformatics. 2009; 10:328.
6. Anders S, Huber W. Differential expression analysis for sequence count data. Genome Biol. 2010; 11:R106.
7. Benjamini YH, Y. Controlling the false discovery rate: a practical and powerful approach to multiple testing. J Roy Stat Soc Ser B Methodol. 1995; 57:289–300.
8. Edgar R, Domrachev M, Lash AE. Gene Expression Omnibus: NCBI gene expression and hybridization array data repository. Nucleic Acids Res. 2002; 30:207-210.
9. Du P, Kibbe WA, Lin SM. lumi: a pipeline for processing Illumina microarray. Bioinformatics. 2008; 24:1547-1548.
10. Lin SM, Du P, Huber W, Kibbe WA. Model-based variance-stabilizing transformation for Illumina microarray data. Nucleic acids research. 2008; 36:e11.
11. Workman Cea. A new non-linear normalization method for reducing variability in DNA microarray experiments. Genome Biology. 2002.
12. Du P, Kibbe WA, Lin SM. nuID: a universal naming scheme of oligonucleotides for illumina, affymetrix, and other microarrays. Biology direct. 2007; 2:16.
13. Smyth GK. Limma: linear models for microarray data. In: Bioinformatics and Computational Biology Solutions using R and Bioconductor. Springer, New York. 2005:397-420.
14. Talantov D, Mazumder A, Yu JX, Briggs T, Jiang Y, Backus J, Atkins D, Wang Y. Novel genes associated with malignant melanoma but not benign melanocytic lesions. Clin Cancer Res. 2005; 11:7234-7242.
15. Riker AI, Enkemann SA, Fodstad O, Liu S, Ren S, Morris C, Xi Y, Howell P, Metge B, Samant RS, Shevde LA, Li W, Eschrich S, Daud A, Ju J, Matta J. The gene expression profiles of primary and metastatic melanoma yields a transition point of tumor progression and metastasis. BMC Med Genomics. 2008; 1:13.
16. Scatolini M, Grand MM, Grosso E, Venesio T, Pisacane A, Balsamo A, Sirovich R, Risio M, Chiorino G. Altered molecular pathways in melanocytic lesions. Int J Cancer. 2010; 126:1869-1881.
17. Cirenajwis H, Ekedahl H, Lauss M, Harbst K, Carneiro A, Enoksson J, Rosengren F, Werner-Hartman L, Torngren T, Kvist A, Fredlund E, Bendahl PO, Jirstrom K, et al.

Molecular stratification of metastatic melanoma using gene expression profiling: Prediction of survival outcome and benefit from molecular targeted therapy. Oncotarget. 2015; 6:12297-12309. doi: 10.18632/oncotarget.3655.

18. Lal A, Navarro F, Maher CA, Maliszewski LE, Yan N, O'Day E, Chowdhury D, Dykxhoorn DM, Tsai P, Hofmann O, Becker KG, Gorospe M, Hide W, Lieberman J. miR-24 Inhibits cell proliferation by targeting E2F2, MYC, and other cell-cycle genes via binding to "seedless" 3'UTR microRNA recognition elements. Mol Cell. 2009; 35:610-625.

19. Durinck S, Moreau Y, Kasprzyk A, Davis S, De Moor B, Brazma A, Huber W. BioMart and Bioconductor: a powerful link between biological databases and microarray data analysis. Bioinformatics. 2005; 21:3439-3440.

20. Durinck S, Spellman PT, Birney E, Huber W. Mapping identifiers for the integration of genomic datasets with the R/Bioconductor package biomaRt. Nat Protoc. 2009; 4:1184-1191.

21. Steinfeld I, Navon R, Ach R, Yakhini Z. miRNA target enrichment analysis reveals directly active miRNAs in health and disease. Nucleic Acids Res. 2013; 41:e45.

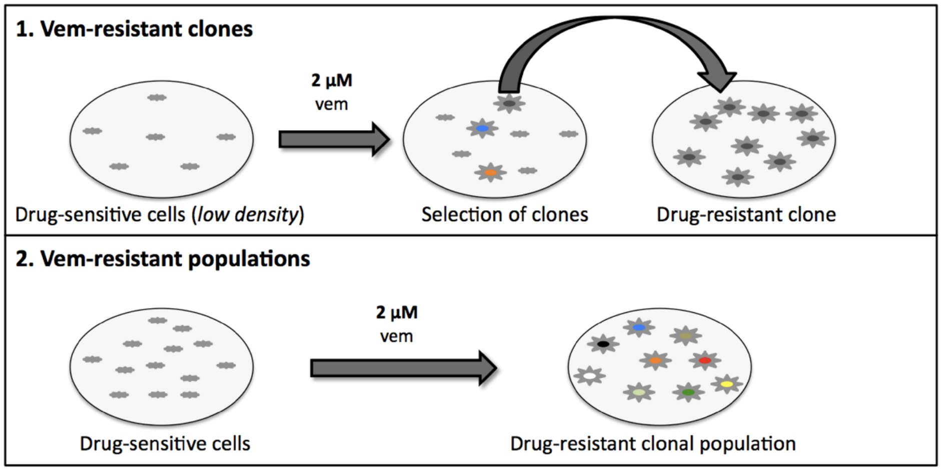

Supplementary Figure 1: Experimental design followed to generate vemurafenib-resistant clones (C) and populations (P) through chronic exposure to 2uM vemurafenib.

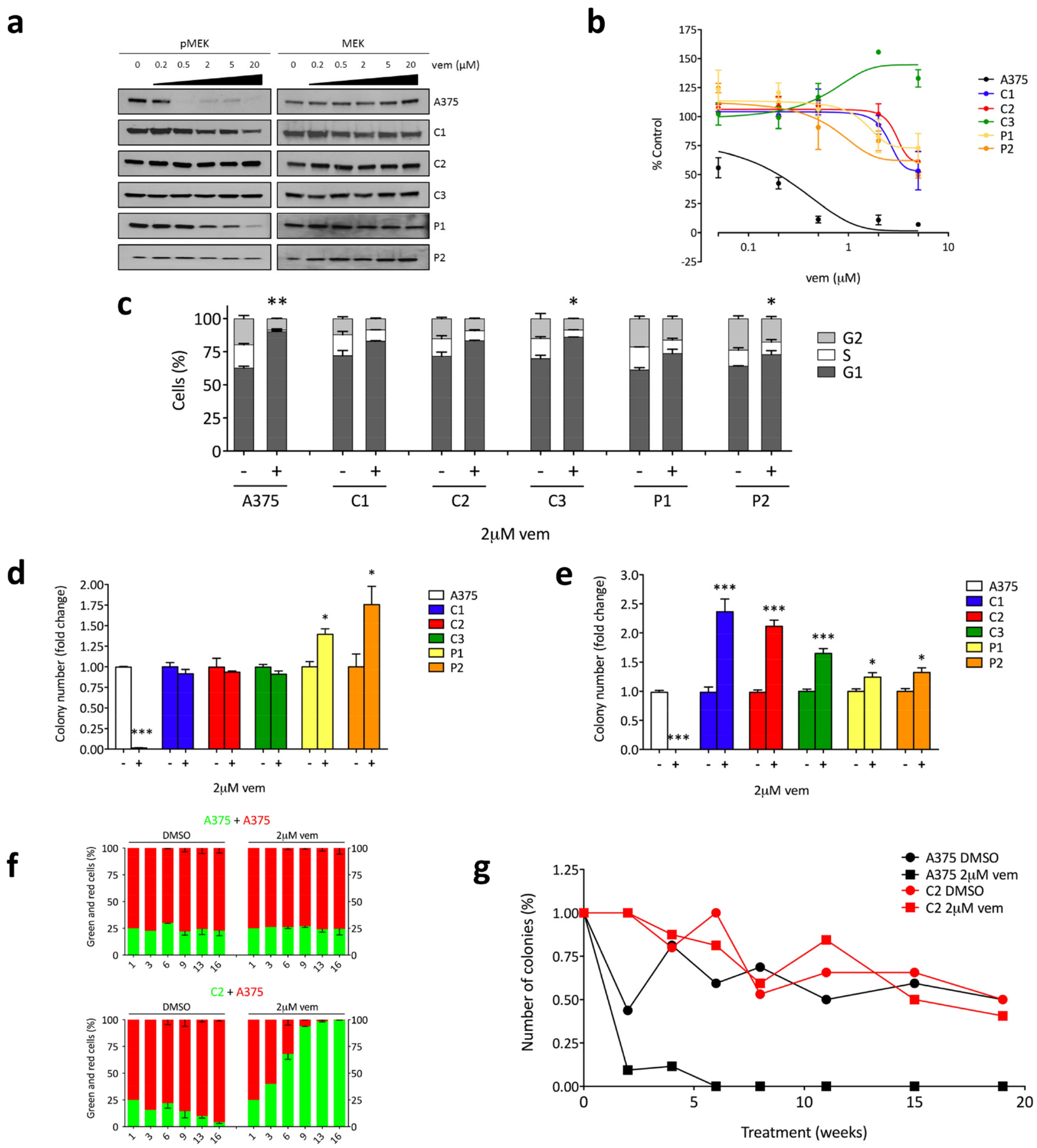

Supplementary Figure 2: Characterization of A375 vemurafenib-resistant clones (C) and populations (P). **(a-e)** C1, C2, C3 resistant clones and P1, P2 resistant populations show decreased sensitivity to vemurafenib, as measured by western blot of phosphoMEK (pMEK) **(a)**, growth curve **(b)**, cell cycle analysis **(c)**, soft agar assay **(d)** and clonogenicity assay **(e)**. **(f)** A375 cells stably expressing mCherry were seeded in a 3:1 ratio together with either A375 or A375 C2 cells stably expressing tGFP. Both cell mixtures were then exposed to either DMSO or 2uM vemurafenib. The percentage of each cell type in the mixture was measured by flowcytometry every 3-4 days. In vemurafenib, A375 C2 cells displace A375 cells within 2 weeks of co-culture. **(g)** Limiting dilution assay. Contrary to A375 cells, A375 C2 cells do not get exhausted upon prolonged vemurafenib exposure. The graphs represent the mean±SEM of 3 independent experiments. *p<0.05, **p<0.01, ***p<0.001.

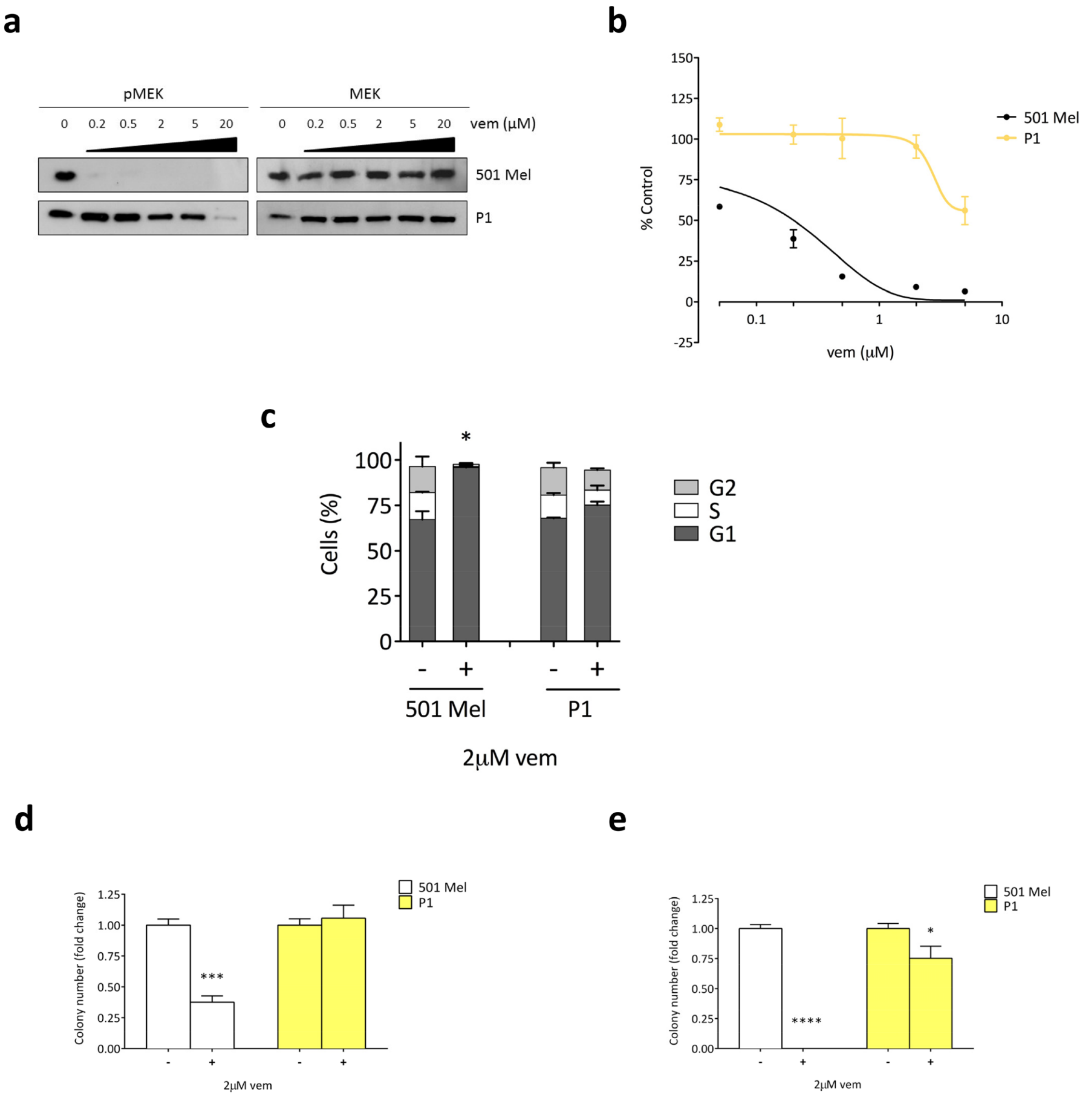

Supplementary Figure 3: Characterization of 501 Mel P1 vemurafenib-resistant population. 501 Mel P1 population shows decreased sensitivity to vemurafenib, as measured by western blot of phosphoMEK (pMEK) **(a)**, growth curve **(b)**, cell cycle analysis **(c)**, soft agar assay **(d)** and clonogenicity assay **(e)**. The graphs represent the mean±SEM of 3 independent experiments.*$p<0.05$, ***$p<0.001$, ****$p<0.0001$.

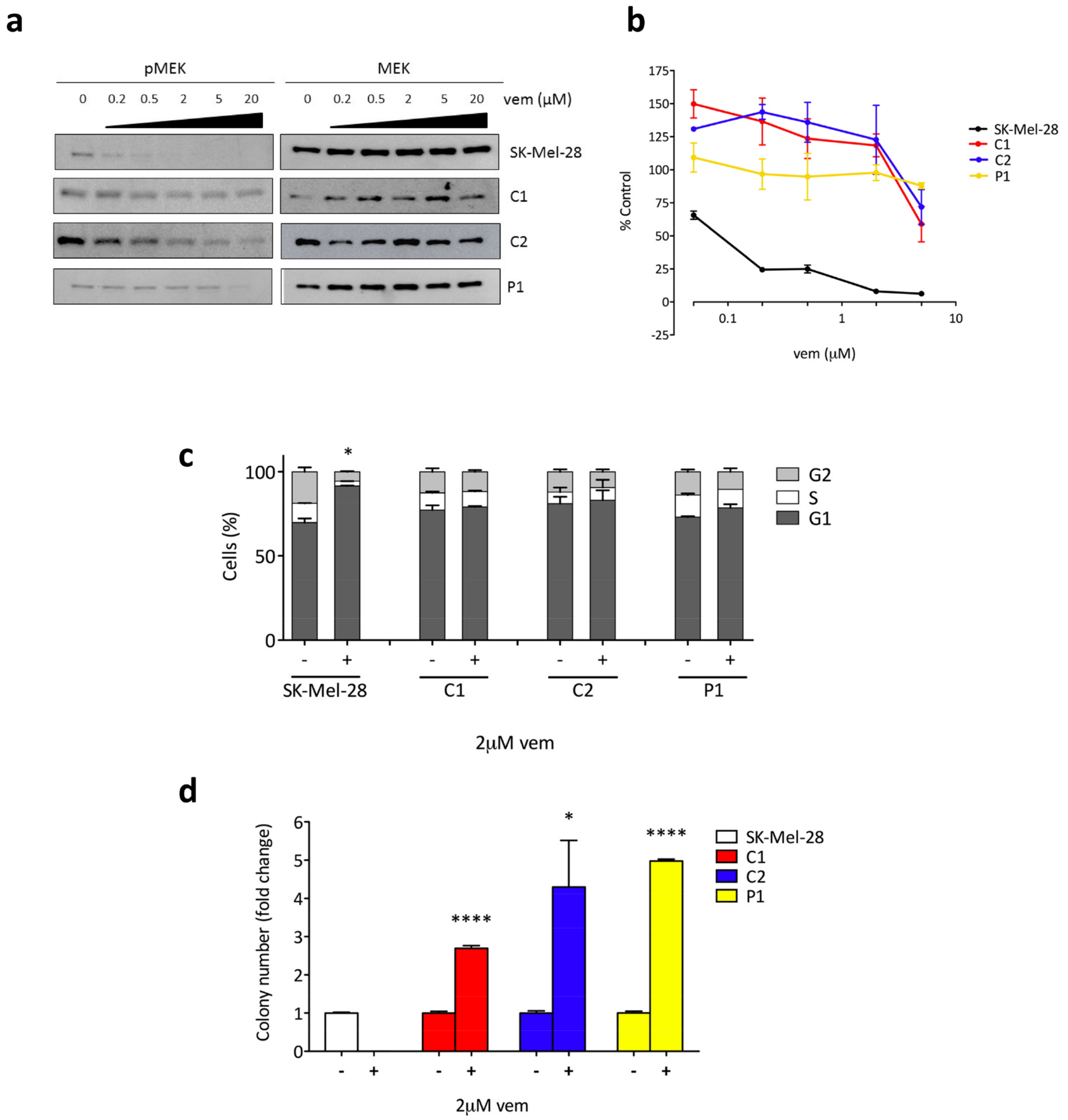

Supplementary Figure 4: Characterization of SK-Mel-28 vemurafenib-resistant clones (C) and populations (P). SK-Mel-28 C1, C2 resistant clones and P1 resistant population show decreased sensitivity to vemurafenib, as measured by western blot of phosphoMEK (pMEK) **(a)**, growth curve **(b)**, cell cycle analysis **(c)** and clonogenicity assay **(d)**. The graphs represent the mean±SEM of 3 independent experiments.*$p<0.05$, ****$p<0.0001$.

a

	Tot seq reads	17-35bp reads after trimming	Number of identified miRNAs
**A375DMSO-1**	24465146	21414554	1277
**A375DMSO-2**	19965992	17574664	1254
**A375vem-1**	27983085	17685467	1210
**A375vem-2**	25647213	17968491	1219
**C2DMSO-1**	15377127	13499806	1229
**C2DMSO-2**	30904489	26909909	1273
**C2vem-1**	23154853	20117872	1248
**C2vem-2**	18806417	16063523	1232
**AVERAGE**	23288040.25	18904285.75	1242.75

b

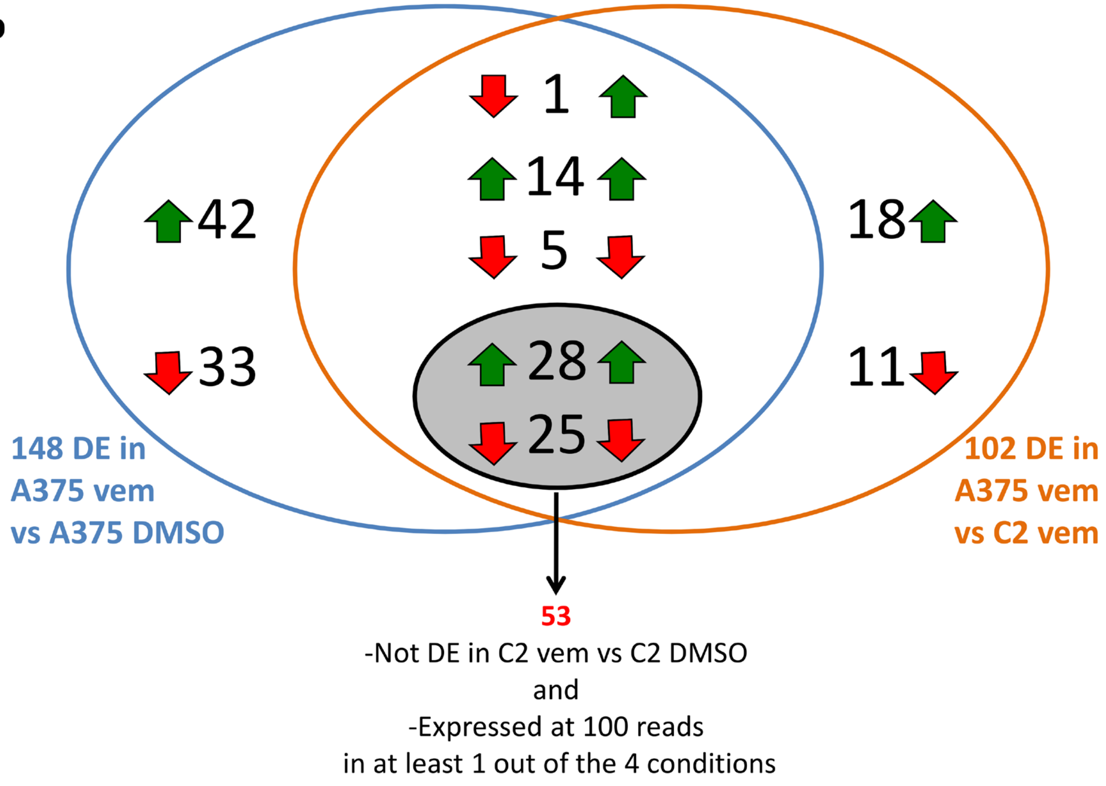

Supplementary Figure 5: Details of the analysis performed on small RNA sequencing data. (a) Number of reads that were obtained and of miRNAs that were identified in each sample. **(b)** Venn diagram that summarizes the results of differential analysis. It highlights the 53 miRNAs that were selected as differentially expressed (DE) and are listed in Figure 2c. The arrows show the direction of the modulation for A375 vemurafenib vs A375 DMSO (left side) and A375 vemurafenib vs C2 vemurafenib (right side). Further details on the experimental protocol and on the analytical steps are provided in the Supplementary Materials and Methods section. A complete list of the miRNAs belonging to each group is reported in Supplementary Table 3-5. Green arrows: higher expression. Red arrows: lower expression.

a

miR-204 isoforms	sequence	Percentages A375 DMSO	A375 vem	C2 DMSO	C2 vem
canonical	uucccuuugucauccuaugccu	29,8%	43.5%	27%	39.7%
3’extended	uucccuuugucauccuaugccug	70.2%	56.5%	73%	60.3%

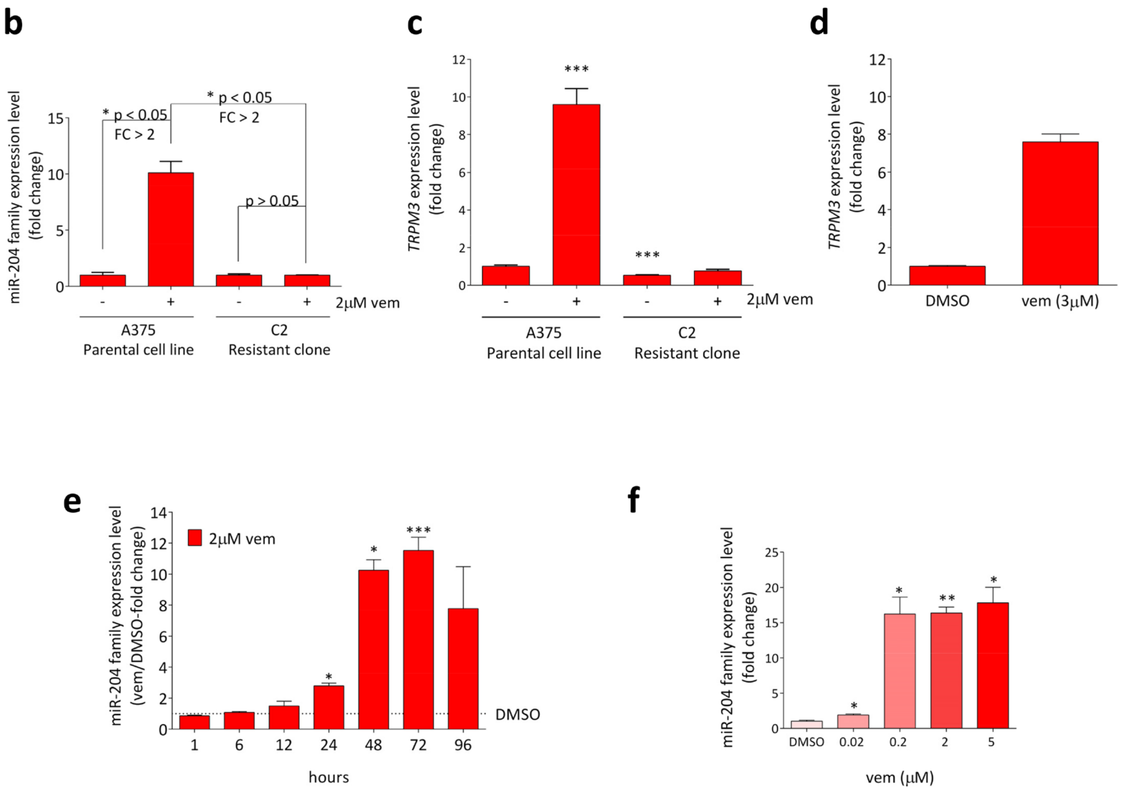

Supplementary Figure 6: Changes in *TRPM3*/miR-204 expression levels upon vemurafenib treatment of A375 cells. (a) Percentages of the canonical and the longer miR-204 isoform detected in each of the 4 experimental conditions analyzed by miRNA-seq. **(b-c)** miR-204 **(b)** and *TRPM3* **(c)** expression levels in A375 parental cell line and C2 vemurafenib-resistant clone after treatment with 2uM vemurafenib for 48h, as measured by real-time PCR. **(d)** *TRPM3* expression levels in A375 cells after treatment with 3uM vemurafenib or vehicle (DMSO) for 24h. The data are derived from PMID 24469106. **(e-f)** miR-204 expression levels in A375 parental cell line after 48h treatment with different doses of vemurafenib **(e)** and after treatment with 2uM vemurafenib for different time points **(f)**. The graphs represent the mean±SEM of 3 independent experiments. *p<0.05, **p<0.01, ***p<0.001.

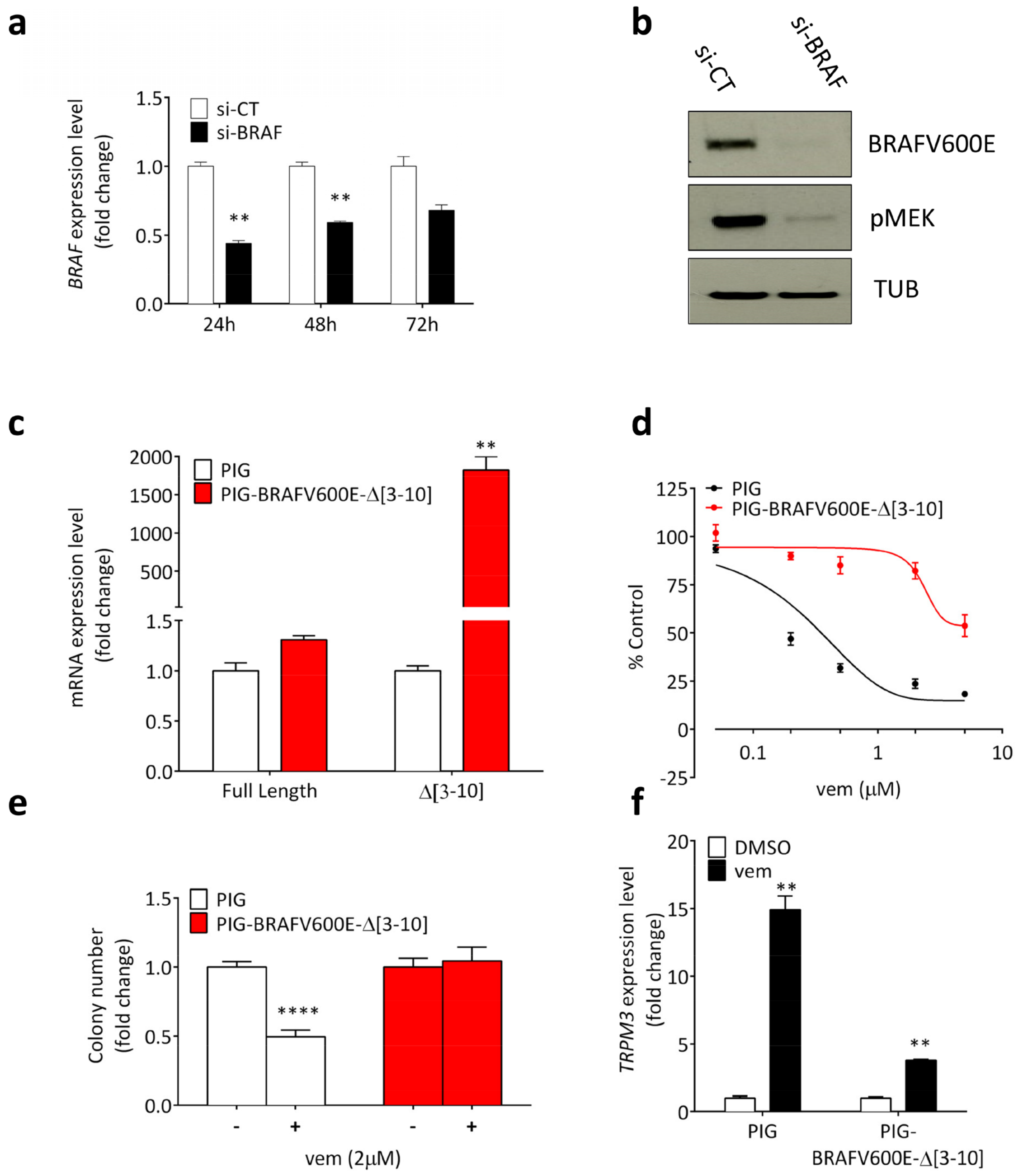

Supplementary Figure 7: Effects of BRAFV600E modulation on miR-204 levels in A375 cells. (a) *BRAF* expression levels at different time points after the transfection of si-BRAF (black) or a control siRNA (si-CT, white). **(b)** BRAF and pMEK protein levels 48h after the transfection of si-CT and si-BRAF. Immunoblotting for α-TUBULIN (TUB) was used as loading control. **(c-f)** Analysis of A375 cells expressing PIG vector or PIG-BRAFV600E-Δ [3–10]. **(c)** The expression levels of full length *BRAFV600E* and *BRAFV600E* Δ [3–10] splicing variant were analyzed by real-time PCR. **(d)** Growth curve: cells were treated with increasing concentrations of vemurafenib for a week to evaluate growth inhibition. **(e)** Clonogenicity assay: cells were treated with 2uM vemurafenib for 10 days and the number of formed colonies were then counted. **(f)** *TRPM3* levels upon vemurafenib treatment: cells were treated with vehicle (DMSO) or 2uM vemurafenib for 48h. *TRPM3* expression levels were then evaluated by real-time PCR. The graphs represent the mean±SEM of 3 independent experiments. **p<0.01, ****p<0.0001.

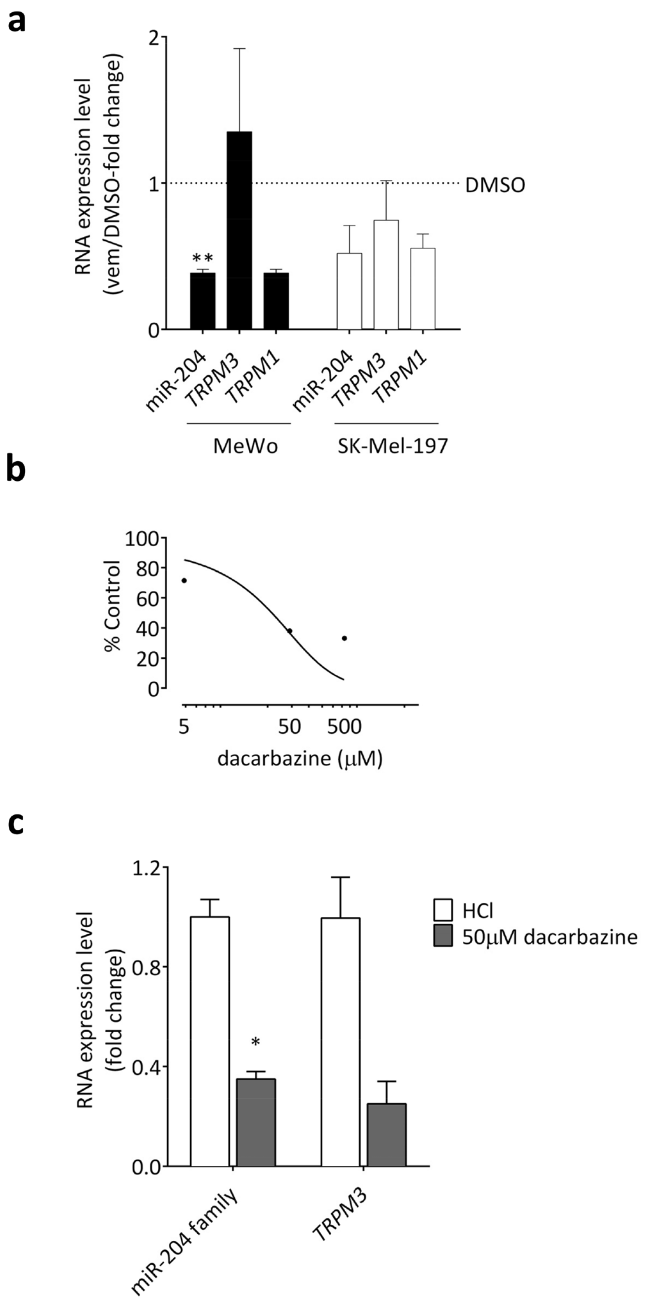

Supplementary Figure 8: miR-204 family expression is not induced in wt BRAF cell lines upon vemurafenib treatment and in A375 cells treated with dacarbazine. **(a)** miR-204 family, *TRPM1* and *TRPM3* expression levels in MeWo and SK-Mel-197 wt BRAF cells after 48h of 2uM vemurafenib treatment. **(b)** Growth curve of A375 cells treated with different doses of dacarbazine. **(c)** miR-204 expression levels in A375 after 48h of treatment with vehicle (HCl, white) or 50uM dacarbazine (grey). The graphs represent the mean±SEM of 3 independent experiments. *p<0.05, **p<0.01.

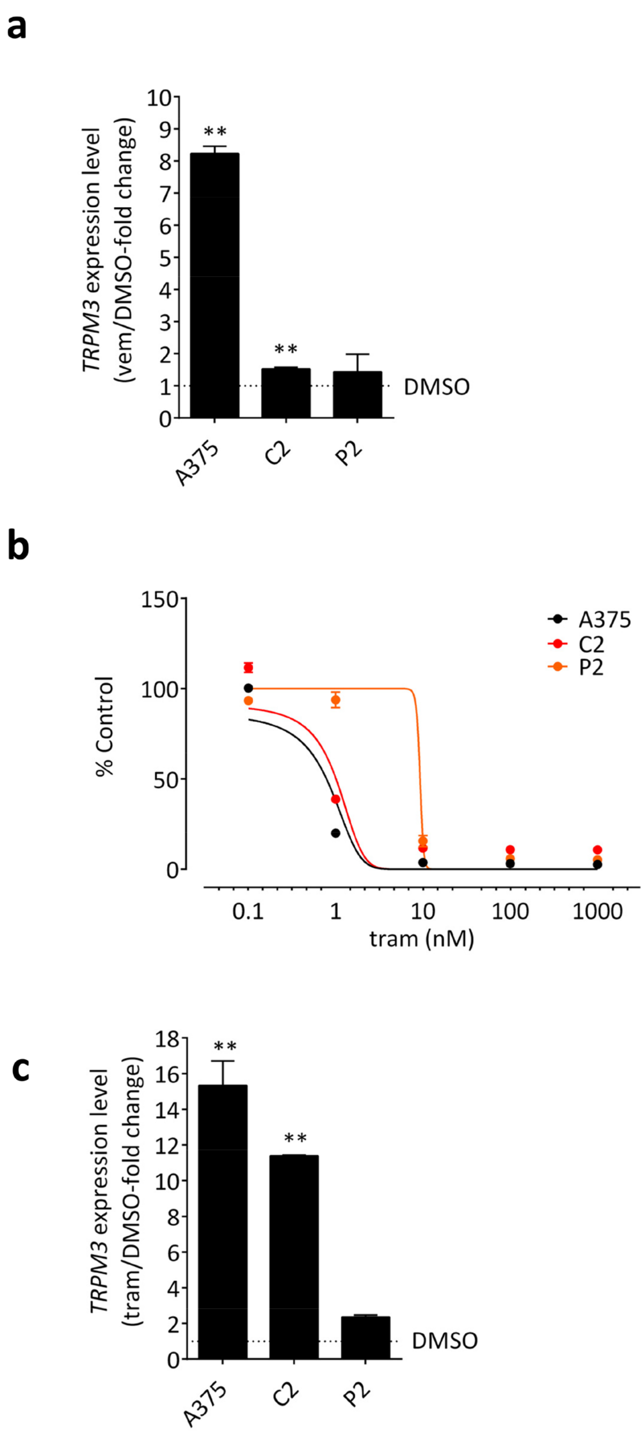

Supplementary Figure 9: Induction of *TRPM3* upon vemurafenib and/or trametinib treatment. **(a)** *TRPM3* expression levels in A375, A375 C2 and A375 P2 after 48h of vemurafenib treatment. **(b)** Growth curve of A375, A375 C2 and A375 P2 cells in presence of increasing doses of trametinib. The A375 parental cell line and the A375 C2 vemurafenib-resistant clone show sensitivity at low doses of trametinib. Conversely, the A375 P2 vemurafenib-resistant population is sensitive to trametinib only at higher doses (10 nM). **(c)** *TRPM3* expression level in A375, A375 C2 and A375 P2 after 48h of 10 nM trametinib treatment. The graphs represent the mean±SEM of 3 independent experiments. **p<0.01.

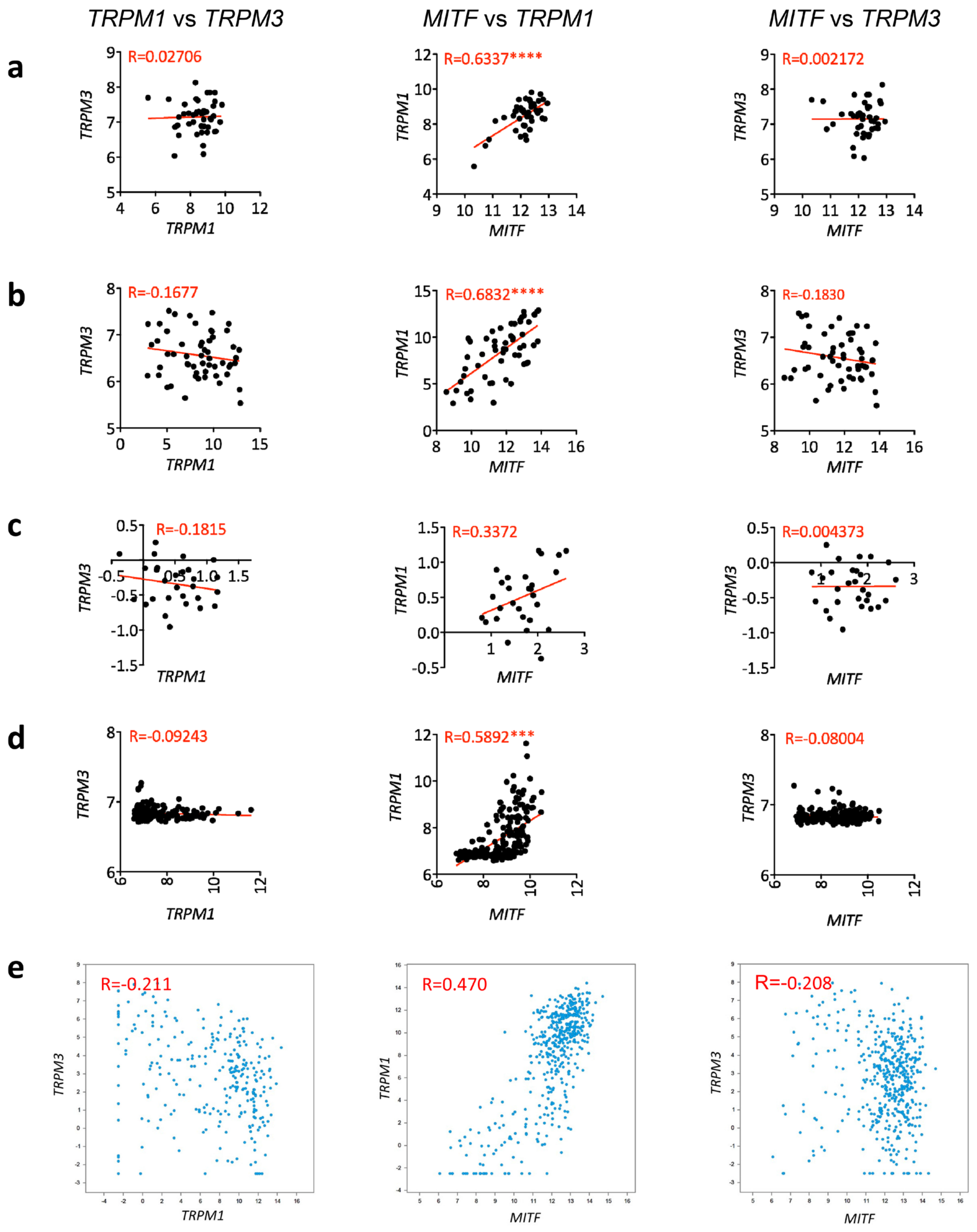

Supplementary Figure 10: Correlation of the expression levels of *TRPM1*, *TRPM3* and *MITF* in multiple published datasets. **(a)** Analysis of 45 cutaneous melanoma cases available at GSE 3189. **(b)** Analysis of 56 cutaneous melanoma cases available at GSE 7553. **(c)** Analysis of 28 cutaneous melanoma cases available at GSE 12391. **(d)** Analysis of 214 cutaneous melanoma cases available at GSE 65904. **(e)** Analysis of 471 cases of cutaneous melanoma available at www.cbioportal.org. The analyses reveal that there is no correlation in the expression levels of *TRPM1* and *TRPM3* (*left panels*). Furthermore, they show that, while *MITF* and *TRPM1* expression levels are positively correlated (*middle panels*), there is no correlation between the levels of expression of *MITF* and *TRPM3* (*right panels*). ***p< 0.001, ****p< 0.0001.

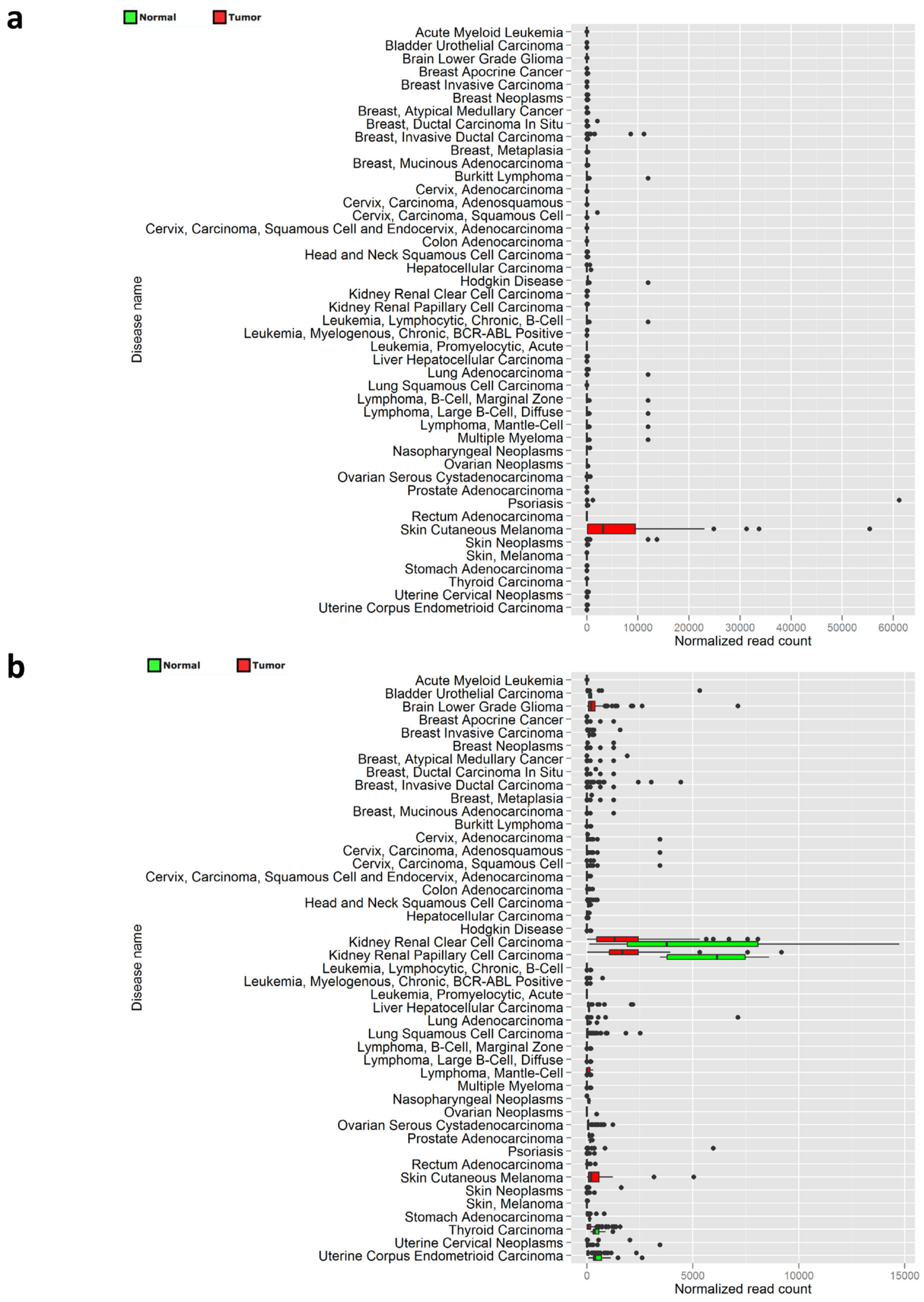

Supplementary Figure 11: Expression levels of miR-211 (a) and miR-204 (b) in various disease and tissue types. The data were obtained from miRGator v3.0 (http://mirgator.kobic.re.kr).

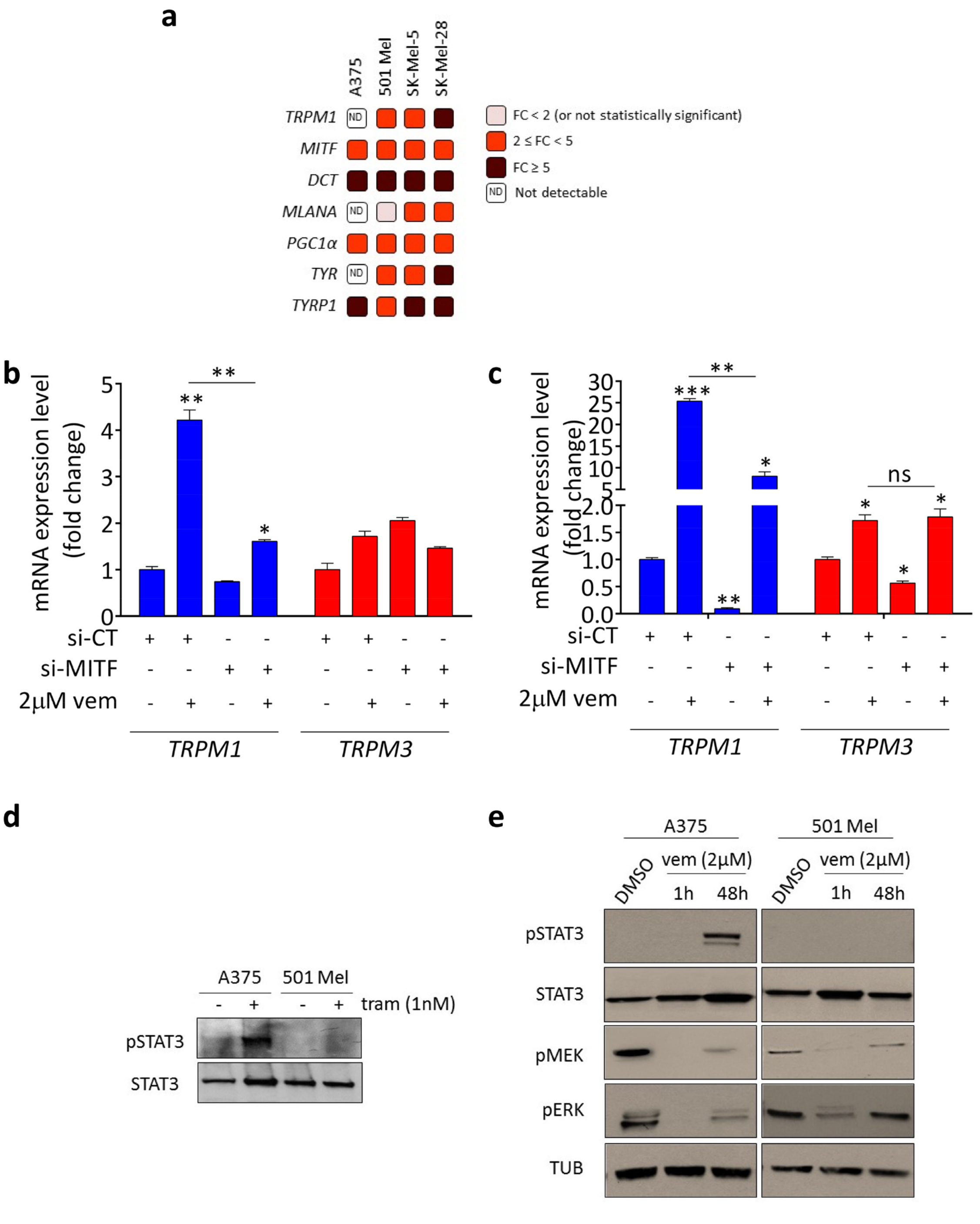

Supplementary Figure 12: *TRPM1*/miR-211 induction depends on MITF, while *TRPM3*/miR-204 induction is associated with STAT3 phosphorylation. **(a)** Expression levels of *MITF* and *MITF* target genes after 48h of vemurafenib treatment in A375, 501 Mel, SK-Mel-5 and SK-Mel-28. **(b-c)** *TRPM1* and *TRPM3* expression levels in SK-Mel-5 **(b)** and WM35 cells **(c)** after 72h from si-MITF transfection and after 48h of vemurafenib treatment. **(d)** Upon 48h of treatment with 1 nM trametinib, STAT3 phosphorylation is induced in A375, but not in 501 Mel cells. **(e)** After 1h of treatment with 2uM vemurafenib, MEK and ERK phosphorylation is inhibited in both A375 and 501 Mel cells. However, after 48h of treatment STAT3 phosphorylation is induced only in A375 cells. The graphs represent the mean±SEM of 3 independent experiments. *p<0.05, **p<0.01, ***p<0.001.

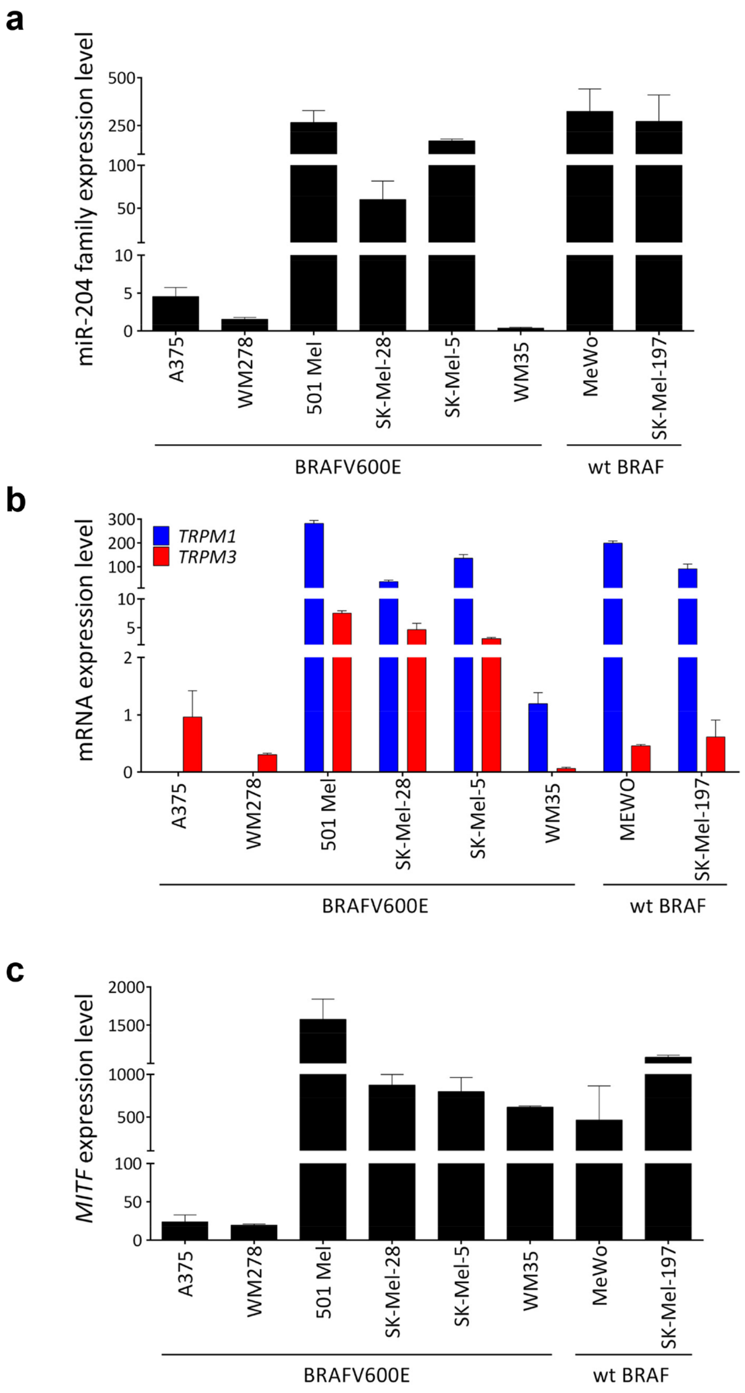

Supplementary Figure 13: Expression levels of miR-204 family (a), *TRPM1*, *TRPM3* (b) and *MITF* (c) in the indicated melanoma cell lines. The graphs represent the mean±SEM of 3 independent experiments.

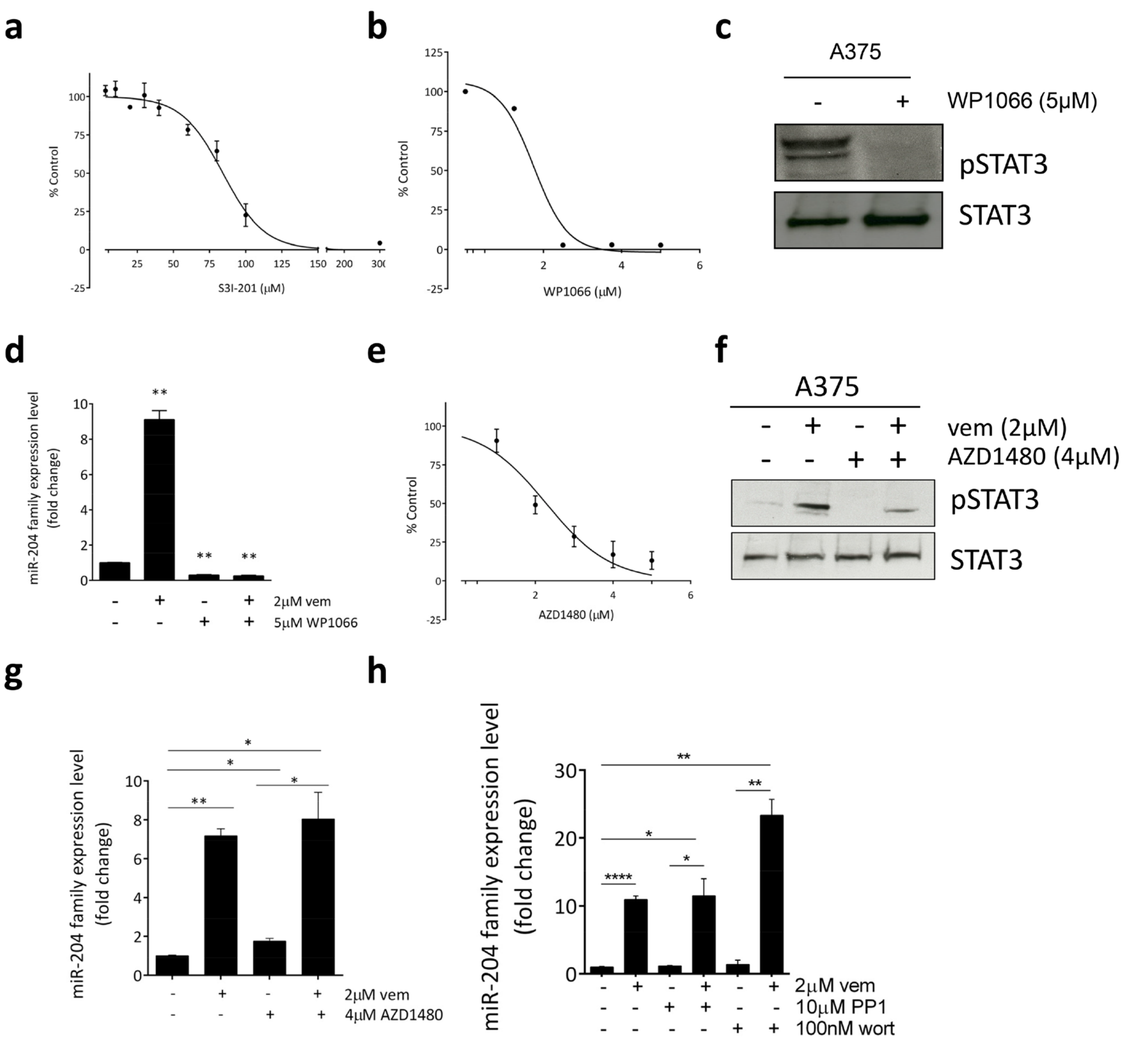

Supplementary Figure 14: JAK2, PI3K and SRC are not mediators of STAT3 activation and TRPM3/miR-204 induction upon vemurafenib treatment. **(a)** Growth curve of A375 cells with increasing doses of the selective STAT3 inhibitor S3I-201. **(b-d)** Characterization of the double JAK2/STAT3 inhibitor WP1066. **(b)** Growth curve of A375 cells with increasing doses of WP1066. **(c)** Western blot showing that 5uM WP1066 abolishes STAT3 phosphorylation. **(d)** The concomitant treatment with 5uM WP1066 abolishes miR-204 induction caused by vemurafenib. **(e-g)** Characterization of the selective JAK2 inhibitor AZD1480. **(e)** Growth curve of A375 cells with increasing doses of AZD1480. **(f)** Western blot showing that 4uM AZD1480 abolishes STAT3 phosphorylation *per se*, but cannot prevent the phosphorylation induced by 2uM vemurafenib. **(g)** The concomitant treatment with 4uM AZD1480 cannot abolish miR-204 induction caused by 2uM vemurafenib. **(h)** Characterization of the SRC inhibitor PP1 and the PI3K inhibitor wortmannin. The concomitant treatment with 10uM PP1 or 100 nM wortmannin cannot abolish miR-204 induction caused by vemurafenib. In order to perform real-time PCR or western blot, A375 cells were treated with the indicated drugs for 48h. The graphs represent the mean±SEM of 3 independent experiments. *p<0.05, **p<0.01, ****p<0.0001.

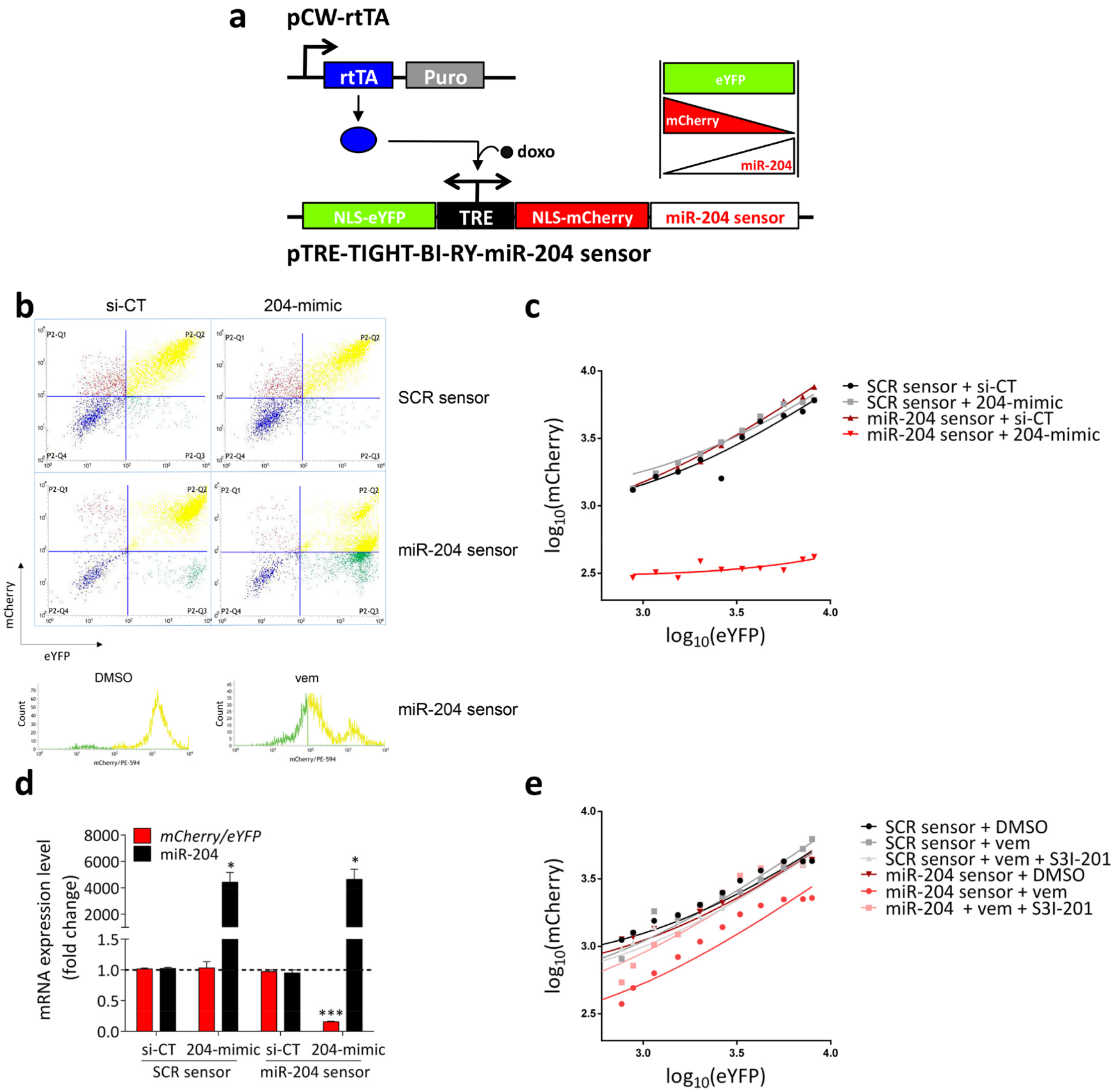

Supplementary Figure 15: A sensor for miR-204. **(a)** Schematic representation of the sensor for miR-204 and its functioning. The pTRE-TIGHT-BI-RY miR-204 sensor consists of a bidirectional and TET-inducible promoter (TRE) that controls the expression of eYFP and mCherry reporter proteins. One copy of the sequence complementary to mature miR-204 is cloned downstream of mCherry ORF. Once the sensor is co-transfected with the pCW-rtTA plasmid and the cells are treated with doxycycline (doxo, black circle), the reverse tetracycline-controlled transactivator (rtTA)-doxo complex is formed, which binds to the TRE promoter and activates the expression of both eYFP and mCherry mRNAs. Since mCherry mRNA is recognized and cleaved by miR-204, the final mCherry protein output results inversely proportional to miR-204 levels. Conversely, the miR-204 insensitive eYFP serves as normalizer. NLS: nuclear localization signal. **(b-c)** Flowcytometry analysis of cells expressing the pTRE-TIGHT-BI-RY scrambled sensor or the pTRE-TIGHT-BI-RY miR-204 sensor and transfected with si-CT or 204-mimic. **(b)** The increased intracellular levels of miR-204 cause a decrease in mCherry fluorescence in the cells that express the miR-204 sensor, but not in those that express the scrambled sensor. **(c)** Processing of flowcytometry data according to PMID 21857679. The right quadrants of each of the 4 panels reported in **(b)** were divided in 10 bins, each encompassing about 10 units of fluorescence intensity. Then, the mean eYFP level and the mean mCherry level were calculated in each bin. These values were log transformed and reported in the graph. In the cells that express the miR-204 sensor, a net drop of mCherry fluorescence intensities is observed when the intracellular levels of miR-204 are increased. **(d)** Real time PCR analysis of the same samples reported in **(b)**. In pTRE-TIGHT-BI-RY miR-204 sensor cells, the increase of endogenous miR-204 levels causes a decrease in the mCherry/eYFP mRNA ratio. **(e)** Processing of the flowcytometry data reported in Figure 4f, following the sequential steps described in **(c)**. In the cells that express the miR-204 sensor, vemurafenib treatment causes a drop of mCherry fluorescence intensities, due to the fact that intracellular miR-204 levels are increased. Such a drop is rescued by the concomitant treatment with the STAT3 inhibitor S3I-201. The graphs represent the mean±SEM of 3 independent experiments. *$p<0.05$, ***$p<0.001$.

a

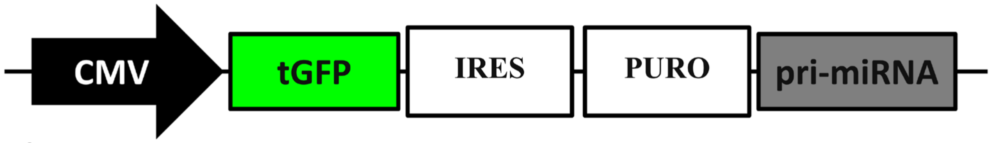

pri-miR-204

(XhoI)CTCGAGgacagggtgatggaaaggagggtgggggtggaggcaagcagaggacttcctgatcgcgtacccat**ggctac**
agtctttcttcatgtgactcgtggacttccctttgtcatcctatgcctgagaatatatgaaggaggctgggaaggcaaagggacgttc
aattgtcatcactggcatctttttttgatcattgcaccatcatcaaatgc***ACGCGT(MluI)***

pri-miR-211

(XhoI)CTCGAGtgatgctgcagagtgggtagaatggggatggcctactaggaaaagttgtagatttccgagaatcagatctcga
agaatacattggtcgatgactgaatgtttttttgttctcacatctctaatttttctggacttgtaagtttttacaacaccccatt**tcacctgg**
ccatgtgacttgtgggcttccctttgtcatccttcgcctagggctctgagcagggcagggacagcaaaggggtgctcagttgtcactt
cccacagcacggagcttcgaggactccagcctaagccaagaagaacctgttctactctgttgctcgaaaagaaggtgatttggccac
catattcctaaagattgaaaatatcaatccttgcttgggttggaaatctcaaaggcggagtccaggata***ACGCGT(MluI)***

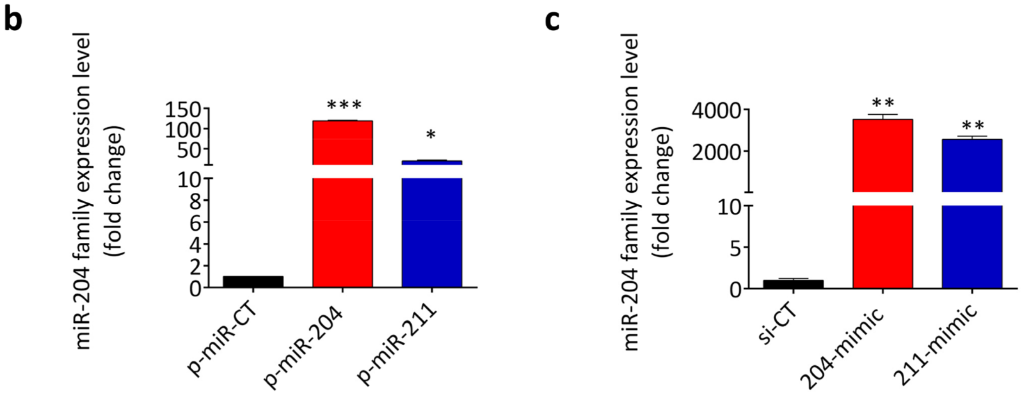

Supplementary Figure 16: Stable and transient over-expression of miR-204 and miR-211 in A375 cells. (a) (*upper*) Schematic representation of the pGIPZ-based lentiviral vector used to achieve the stable over-expression of miR-204 and miR-211. tGFP: turbo GFP. (*lower*) Sequence of the pri-miR-204 and pri-miR-211 that were cloned in the pGIPZ-miR-CT vector using the XhoI and MluI cloning sites. The pre-miRNA sequence is shown in bold and the mature miRNA sequence is underlined. **(b)** Fold increase in endogenous miR-204 and miR-211 levels upon the stable infection of pGIPZ-miR-204 (p-miR-204) and pGIPZ-miR-211 (p-miR-211) in A375 cells. **(c)** Fold increase in miR-204 and miR-211 levels upon the transient transfection of the corresponding miRNA mimics in A375 cells. The graphs represent the mean±SEM of 3 independent experiments. *p<0.05, **p<0.01, ***p<0.001.

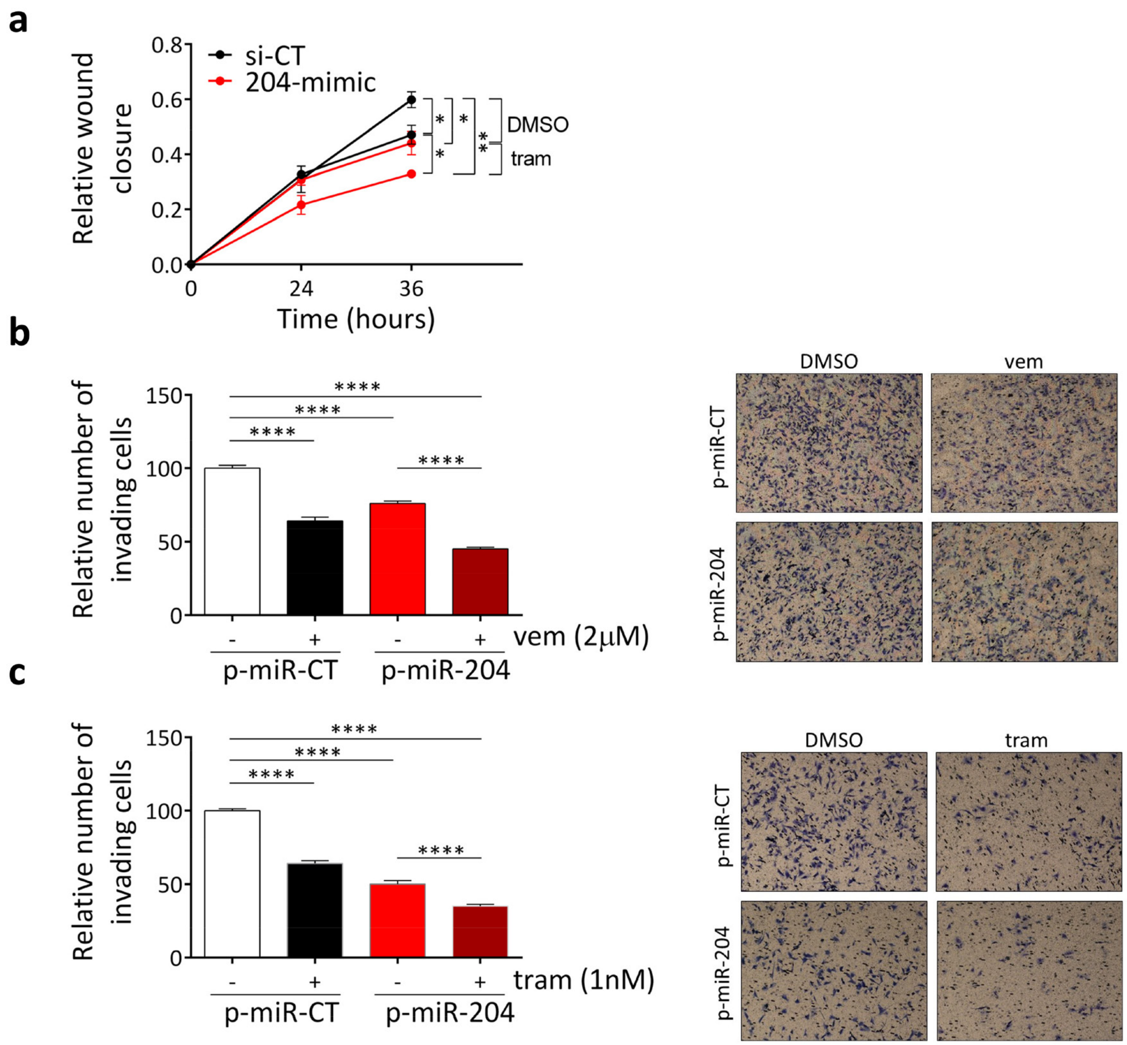

Supplementary Figure 17: miR-204 inhibits the migration and invasion of melanoma cells and cooperates with vemurafenib. (a) Wound closure of SK-Mel-2 cells transfected with a control siRNA (si-CT, black) or miR-204 mimic (204-mimic, red) and treated for the indicated time points with vehicle (DMSO) or 1 nM trametinib. Before being subjected to the assay, the cells were pretreated for additional 24h. **(b)** Matrigel invasion assay performed on WM278 cells that stably over-express a control miRNA (p-miR-CT, black) or miR-204 (p-miR-204, red) and that were treated with 2uM vemurafenib for 24h. **(c)** Matrigel invasion assay performed on SK-Mel-2 cells that stably over-express a control miRNA (p-miR-CT, black) or miR-204 (p-miR-204, red) and that were treated with 1 nM trametinib for 6h. The graphs represent the mean±SEM of 3 independent experiments. *$p<0.05$, **$p<0.01$, ****$p<0.0001$.

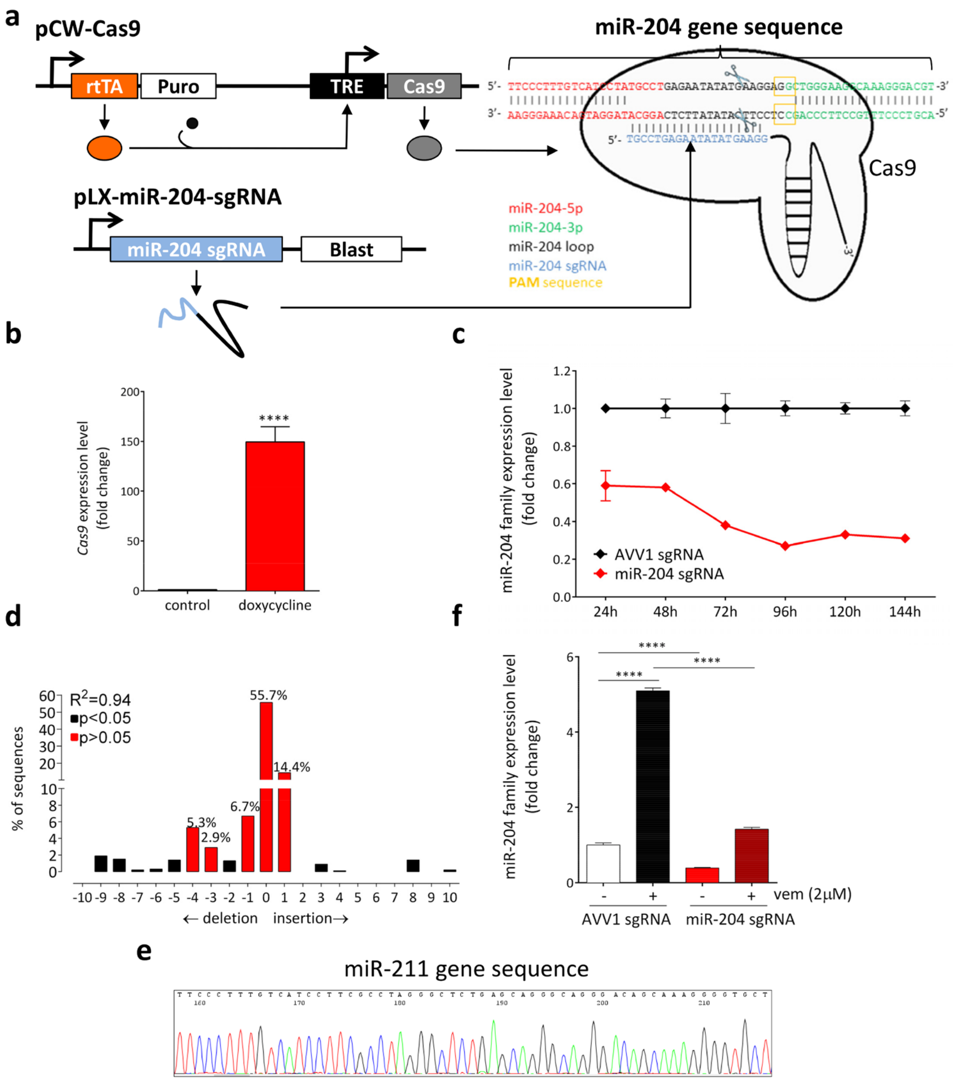

Supplementary Figure 18: CRISPR/Cas9-mediated down-regulation of endogenous miR-204 levels. (a) Cartoon summarizing the CRISPR/Cas9 system. The pCW-Cas9 lentiviral vector expresses the reverse tetracycline-controlled transactivator (rtTA, orange), as well as the Cas9 protein under the control of the TET-inducible promoter (TRE). The pLX-miR-204-sgRNA lentiviral vector expresses the miR-204 sgRNA, which is composed of a CRISPR RNA (crRNA (light blue): it is a 20nt long sequence which is perfectly complementary to the loop region of pre-miR-204) and a transactivating RNA (tracrRNA (black): it is an RNA sequence required by Cas9 in order to exert its catalytic activity). The two plasmids are sequentially infected and A375 cells that express both of them are selected by means of their resistance to puromycin and blasticidin. When doxycycline (doxo, black circle) is added to the double-infected cells, the expression of Cas9 is induced by the binding of the rtTA-doxo complex to the TRE promoter. In turn, Cas9 binds to the sgRNA and is guided to the loop sequence of pre-miR-204. Finally, Cas9 recognizes the PAM sequence and cuts the DNA few nt upstream. In the absence of a template, the cells repair the DNA damage by introducing mutations. Therefore, the final output of the process is the alteration of miR-204 gene sequence, which results in impaired expression, hence in decreased levels of endogenous mature miR-204. Green: miR-204-3p sequence; red: miR-204-5p sequence; black: pre-miR-204 loop sequence; light blue/black: miR-204 sgRNA sequence; yellow rectangle: PAM sequence. As negative control a non-targeting AVV1 sgRNA is used throughout the experiments. **(b)** *Cas9* expression levels after 48h of induction of double-infected A375 cells with doxycycline (doxo, red) compared to the non-induced cells (white). **(c)** EndogenousmiR-204 expression levels in A375 cells that stably express the control sgRNA (AVV1, black) or the miR-204 sgRNA (red) at different time points after Cas9 induction by doxycycline treatment. **(d)** Analysis of the alterations present along miR-204 gene sequence 72h after Cas9 induction in A375 cells that stably express the miR-204 sgRNA. The graph represents the result of the decomposition of the genome editing effects, as determined by http://tide.nki.nl/. **(e)** Electropherogram of miR-211 gene sequence in A375 cells that stably express miR-204 sgRNA 72h after Cas9 induction with doxycycline. The absence of mutations confirms that the miR-204 sgRNA mediates the selective recruitment of Cas9 on miR-204 gene, while leaving miR-211 gene unaltered. **(f)** EndogenousmiR-204 expression levels in A375 cells that stably express the control AVV1 sgRNA (white and black) or the miR-204 sgRNA (red and dark red) after 72h of Cas9 induction by doxycycline treatment and 48h of treatment with 2uM vemurafenib. The graphs represent the mean±SEM of 3 independent experiments. ****p<0.0001.

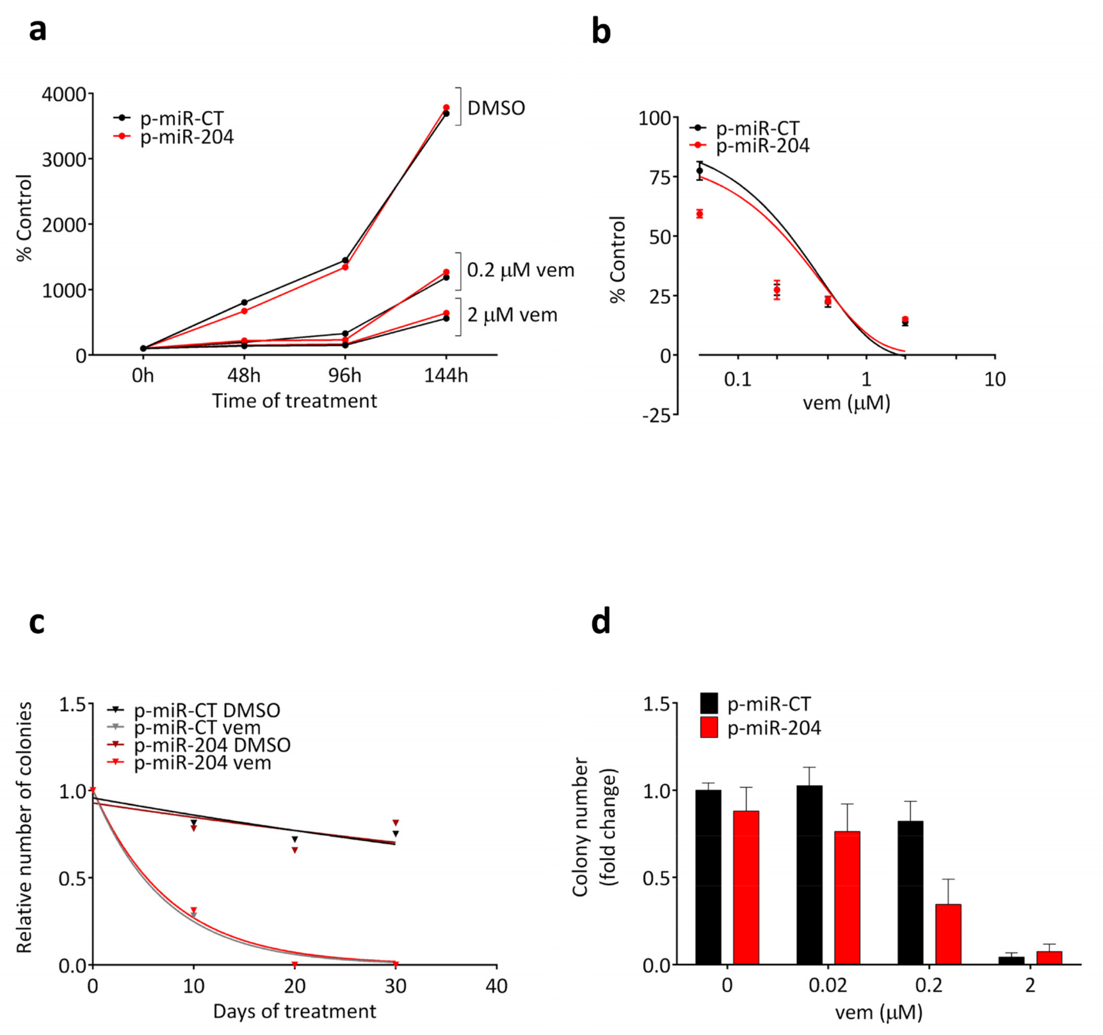

Supplementary Figure 19: Effects of miR-204 over-expression on the growth of A375 cells. (a) Growth curve of A375 cells that stably over-express a control miRNA (p-miR-CT, black) or miR-204 (p-miR-204, red) in presence of DMSO or 0.2 and 2uM vemurafenib. **(b)** Growth curve of A375 cells that overexpress a control miRNA (p-miR-CT, black) or miR-204 (p-miR-204, red) in presence of increasing concentrations of vemurafenib. **(c)** Limiting dilution assay performed on A375 cells that stably overexpress a control miRNA (p-miR-CT, black) or miR-204 (p-miR-204, red) in presence of DMSO or 2uM vemurafenib. **(d)** Clonogenicity assay performed on A375 cells that overexpress a control miRNA (p-miR-CT, black) or miR-204 (p-miR-204, red) in presence of DMSO or 0.02, 0.2 and 2uM vemurafenib. In all the reported assays the stable over-expression of miR-204 has no consequences on the growth of A375 cells. The graphs represent the mean±SEM of 3 independent experiments.

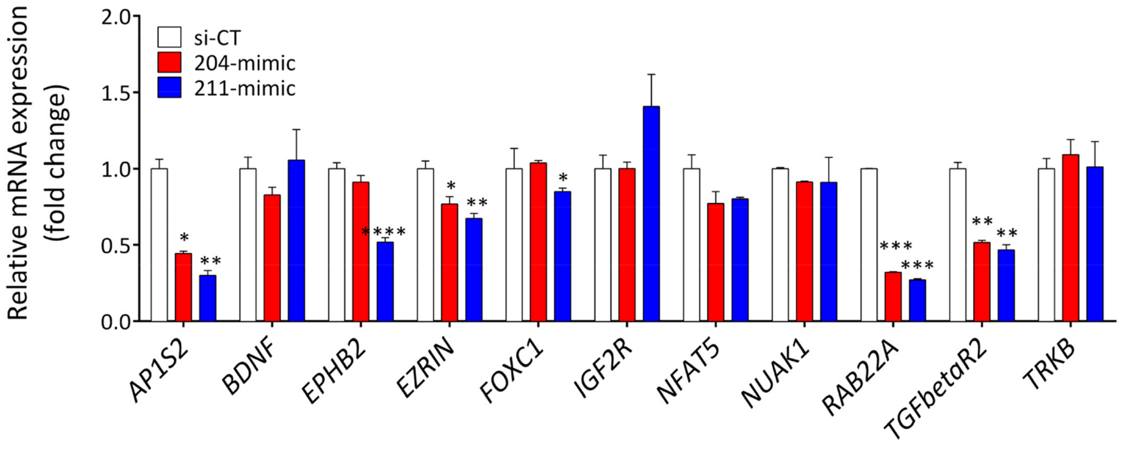

Supplementary Figure 20: Expression levels of pro-motility genes that are known miR-204 or miR-211 targets. mRNA levels are measured by real-time PCR 24h after the transient transfection of si-CT (white), 204-mimic (red) or 211-mimic (blue). The graphs represent the mean±SEM of 3 independent experiments. *p<0.05, **p<0.01, ***p<0.001, ****p<0.0001.

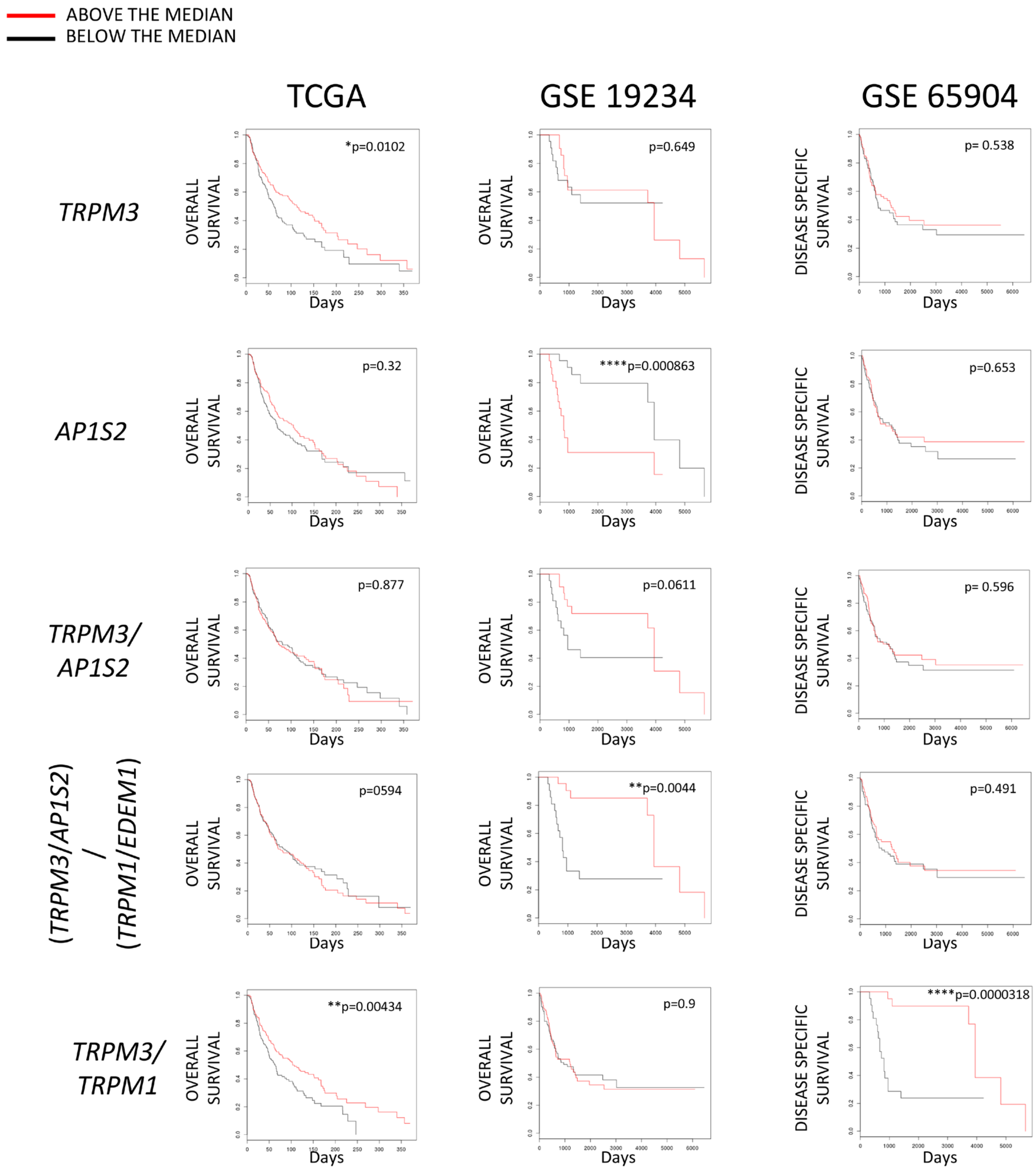

Supplementary Figure 21: Analysis of 3 publicly available datasets of metastatic melanoma samples. The 472 metastatic melanoma samples available at TCGA, the 44 metastatic melanoma samples available at GSE 19234 and the 214 metastatic melanoma samples available at GSE 65904 were divided at the median of expression levels of the listed mRNAs. Survival curves were then calculated for high (above the median, red) and low (below the median, black) expressors. *p<0.05, **p<0.01, ****p<0.0001.

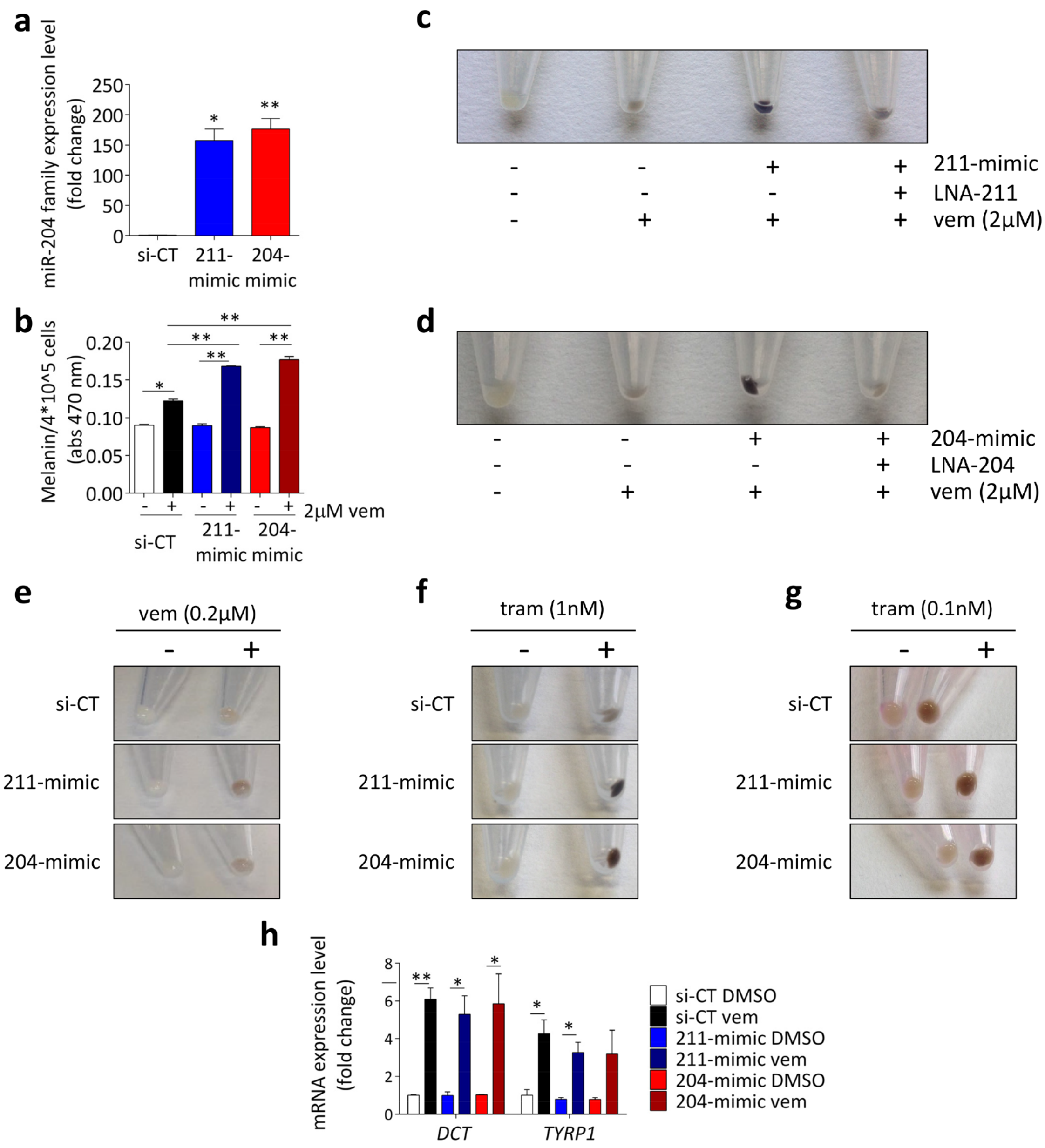

Supplementary Figure 22: miR-204 family over-expression induces an increase in pigmentation. **(a)** miR-204 family expression levels in 501 Mel transfected with si-CT, 211-mimic or 204-mimic. **(b)** Melanin quantification relative to Figure 7a. **(c-d)** The induction of pigmentation caused by 211-mimic **(c)** and 204-mimic **(d)** is impaired by the concomitant transfection of the appropriate LNA. Cells were treated with 2uM vemurafenib 24h after the transfection and pelleted 72h later. **(e-g)** Melanin content in SK-Mel-5 **(e, g)** and 501 Mel **(f)** after 96h from si-CT, 211-mimic or 204-mimic transfection and after 72h of vemurafenib **(e)** or trametinib **(f-g)** treatment. **(h)** Expression levels of the genes belonging to the melanin biosynthetic pathway (*DCT* and *TYRP1)* after 96h from the transfection si-CT, 211-mimic or 204-mimic and after 72h of treatment with 2uM vemurafenib. The graphs represent the mean±SEM of 3 independent experiments. *p<0.05, **p<0.01.

a

miR-204/211 seed	Seed match length	211-mimic modulated transcripts with seed match in 3'UTR	204-mimic modulated transcripts with seed match in 3'UTR	
AAGGGA or AAAGGGA or CAAAGGGA	hexamer (2-7) heptamer (2-8) octamer (2-9)	67 out of 72 ($p=3.5 \times 10^{-11}$)	39 out of 42 ($p=6.5 \times 10^{-7}$)	Down-regulated mRNAs
		54 out of 85 (p=0.19)	42 out of 85 (p=0.06)	Up-regulated mRNAs

b

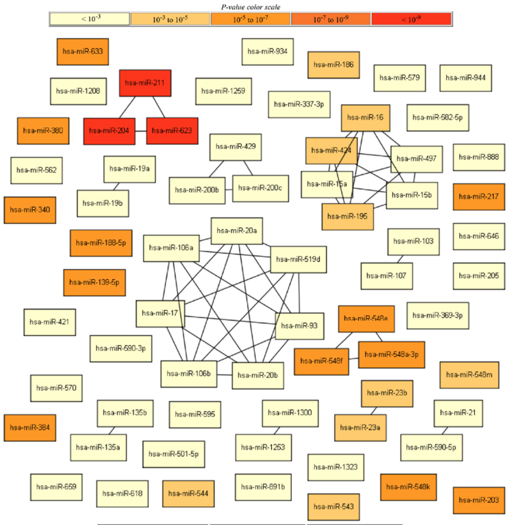

miRNA	p-value	FDR q-value
hsa-miR-204	3.14E-69	2.12E-66
hsa-miR-211	1.27E-68	4.31E-66
hsa-miR-623	1.16E-44	2.63E-42
hsa-miR-203	1.33E-9	2.25E-7
hsa-miR-548e	1.43E-9	1.94E-7
hsa-miR-188-5p	1.89E-9	2.13E-7
hsa-miR-548f	5.18E-9	5.01E-7

c

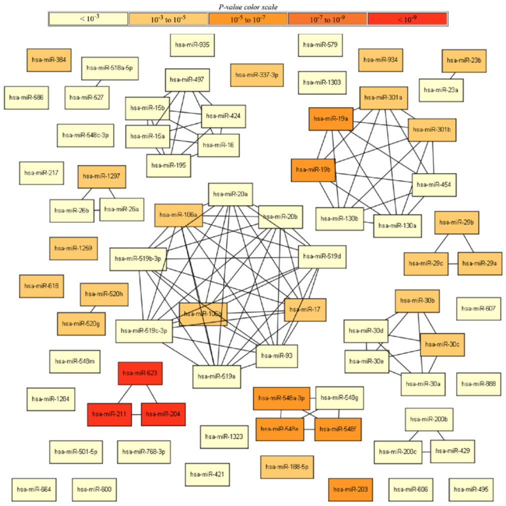

miRNA	p-value	FDR q-value
hsa-miR-204	5.42E-50	3.67E-47
hsa-miR-211	2.47E-49	8.36E-47
hsa-miR-623	5.46E-32	1.23E-29
hsa-miR-548a-3p	4.28E-9	7.25E-7
hsa-miR-203	6.98E-9	9.45E-7
hsa-miR-19a	2.93E-8	3.31E-6
hsa-miR-548f	3.73E-8	3.61E-6

Supplementary Figure 23: Methods to determine if the transcripts down-regulated upon the transfection of 211-mimic and 204-mimic are enriched in putative direct targets. (a) Seed enrichment analysis. We examined the frequency of 3'UTR sequences that contain a seed match that is perfectly complementary to the miR-204/211 seed (AAGGGA 2-7 hexamer, AAAGGGA 2-8 heptamer, and CAAAGGGA 2-9 octamer). We found that 67 out of the 72 211-mimic-downregulated transcripts and 39 out of the 42 204-mimic-downregulated transcripts contain such a seed match in their 3'UTR ($p = 3.5 \times 10^{-11}$ and $p=6.5 \times 10^{-7}$ relative to the background frequency of the seed in the full set of array transcripts, respectively). On the contrary, the frequency of seed matches in the transcripts upregulated upon 204-mimic and 211-mimic over-expression did not differ statistically from the background. **(b-c)** miRNA target enrichment analysis (miTEA). We inferred which is the miRNA that is the most likely candidate to explain the results obtained upon 211-mimic **(b)** and 204-mimic **(c)** transfection and we found that the top-scoring miRNAs are indeed miR-204 and miR-211. The high frequency of transcripts containing perfect seed matches for miR-211 and miR-204, as well as the fact that the signature of modulated transcripts is consistent with miR-211 and miR-204 activity are strongly indicative that the downregulated mRNAs that we obtained upon 211-mimic and 204-mimic transfection are enriched in direct miR-211/204 targets.

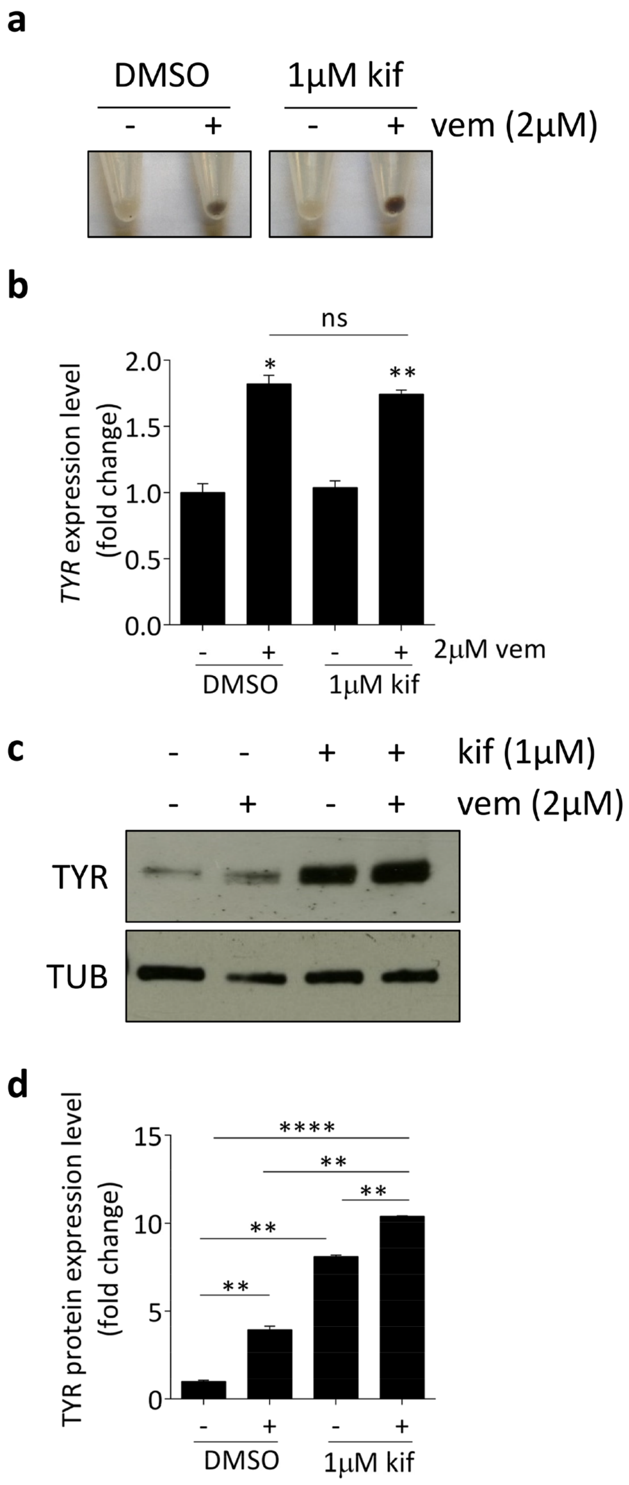

Supplementary Figure 24: Effects of kifunensine on melanin content and TYR levels in 501 Mel cells. **(a)** Pigmentation of 501 Mel cells after 72h of treatment with 2uM vemurafenib and 1uM kifunensine. **(b-d)** TYR RNA **(b)** and protein levels **(c-d)** detected in the same conditions shown in **(a)**. The graphs represent the mean±SEM of 3 independent experiments. *p<0.05, **p<0.01, ****p<0.0001.

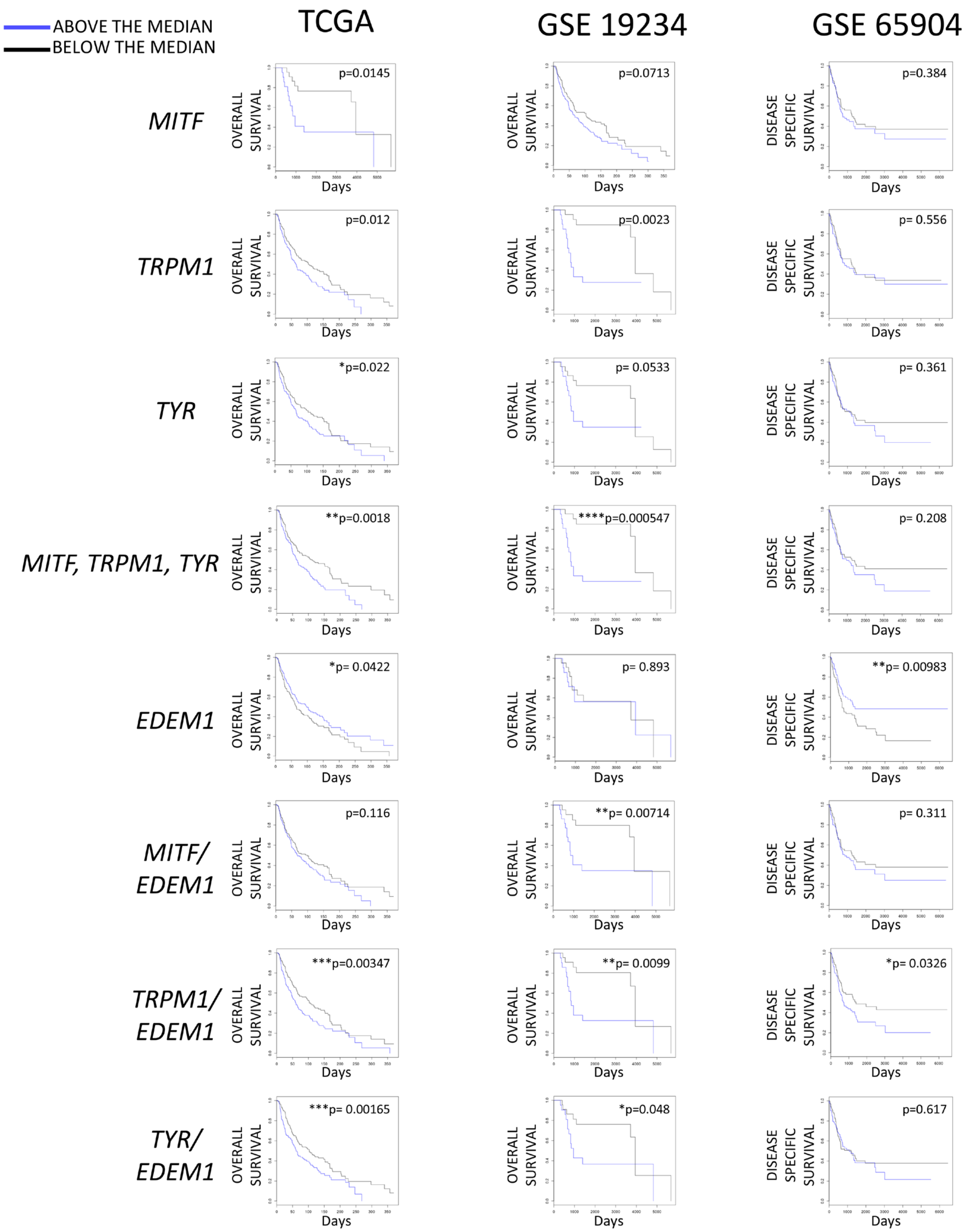

Supplementary Figure 25: Analysis of 3 publicly available datasets of metastatic melanoma samples. The 472 metastatic melanoma samples available at TCGA, the 44 metastatic melanoma samples available at GSE 19234 and the 214 metastatic melanoma samples available at GSE 65904 were divided at the median of expression levels of the listed mRNAs. Survival curves were then calculated for high (above the median, blue) and low (below the median, black) expressors. *p<0.05, **p<0.01, ***p<0.001, ****p<0.0001.

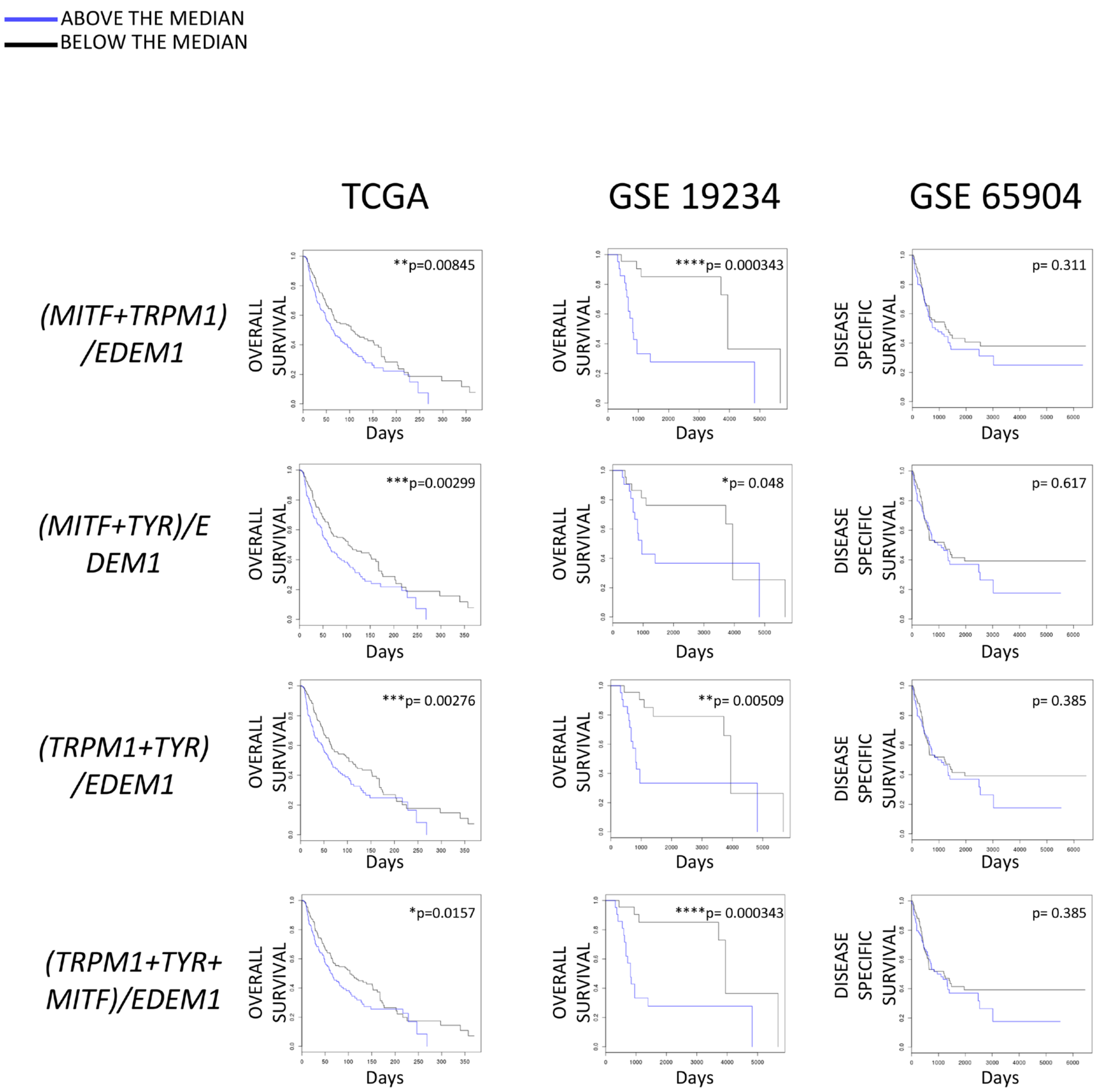

Supplementary Figure 26: Analysis of 3 publicly available datasets of metastatic melanoma samples. The 472 metastatic melanoma samples available at TCGA, the 44 metastatic melanoma samples available at GSE 19234 and the 214 metastatic melanoma samples available at GSE 65904 were divided at the median of expression levels of the listed mRNAs. Survival curves were then calculated for high (above the median, blue) and low (below the median, black) expressors. *p<0.05, **p<0.01, ***p<0.001, ****p<0.0001.

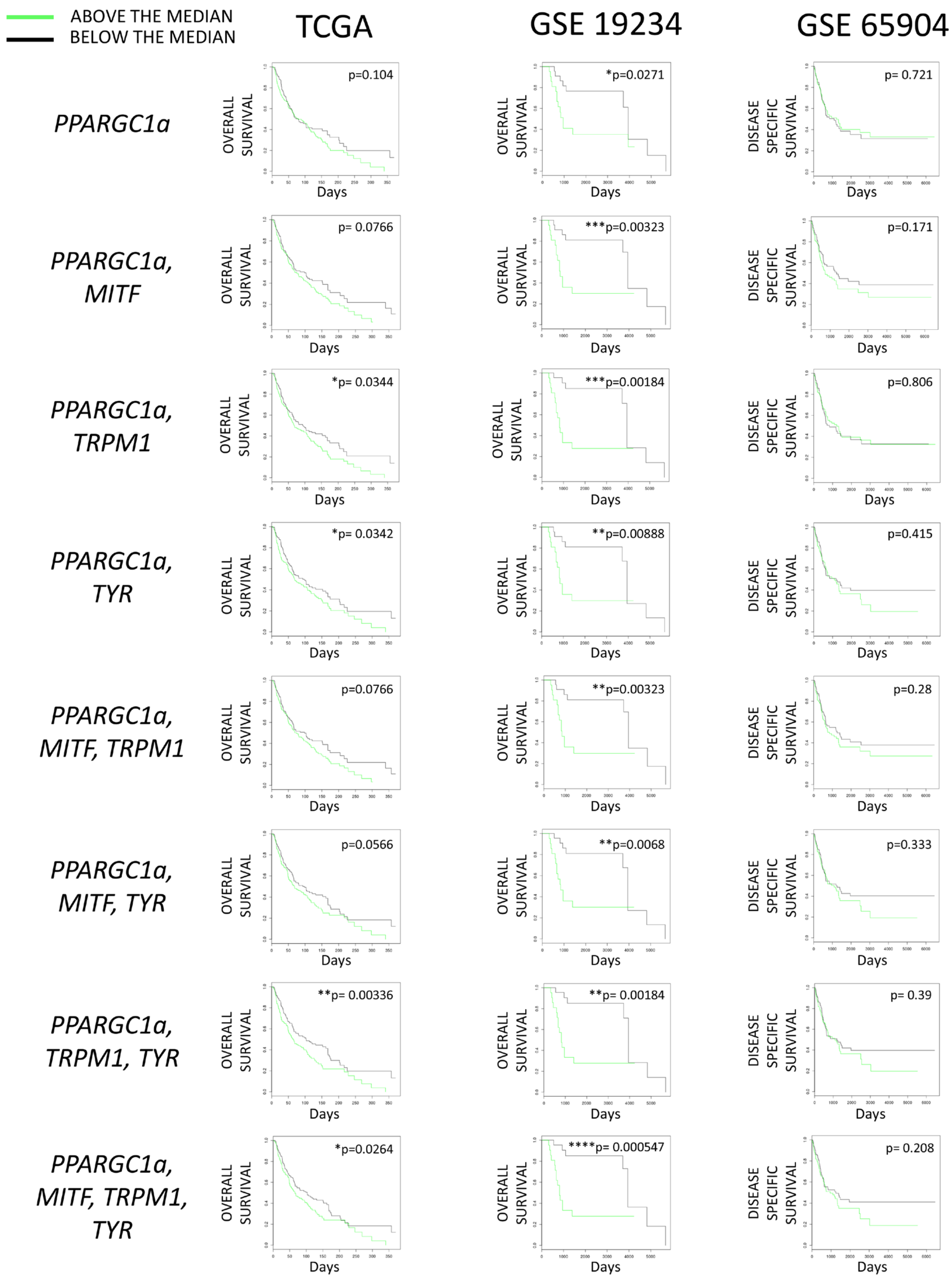

Supplementary Figure 27: Analysis of 3 publicly available datasets of metastatic melanoma samples. The 472 metastatic melanoma samples available at TCGA, the 44 metastatic melanoma samples available at GSE 19234 and the 214 metastatic melanoma samples available at GSE 65904 were divided at the median of expression levels of the listed mRNAs. Survival curves were then calculated for high (above the median, green) and low (below the median, black) expressors. *p<0.05, **p<0.01, ***p<0.001, ****p<0.0001.

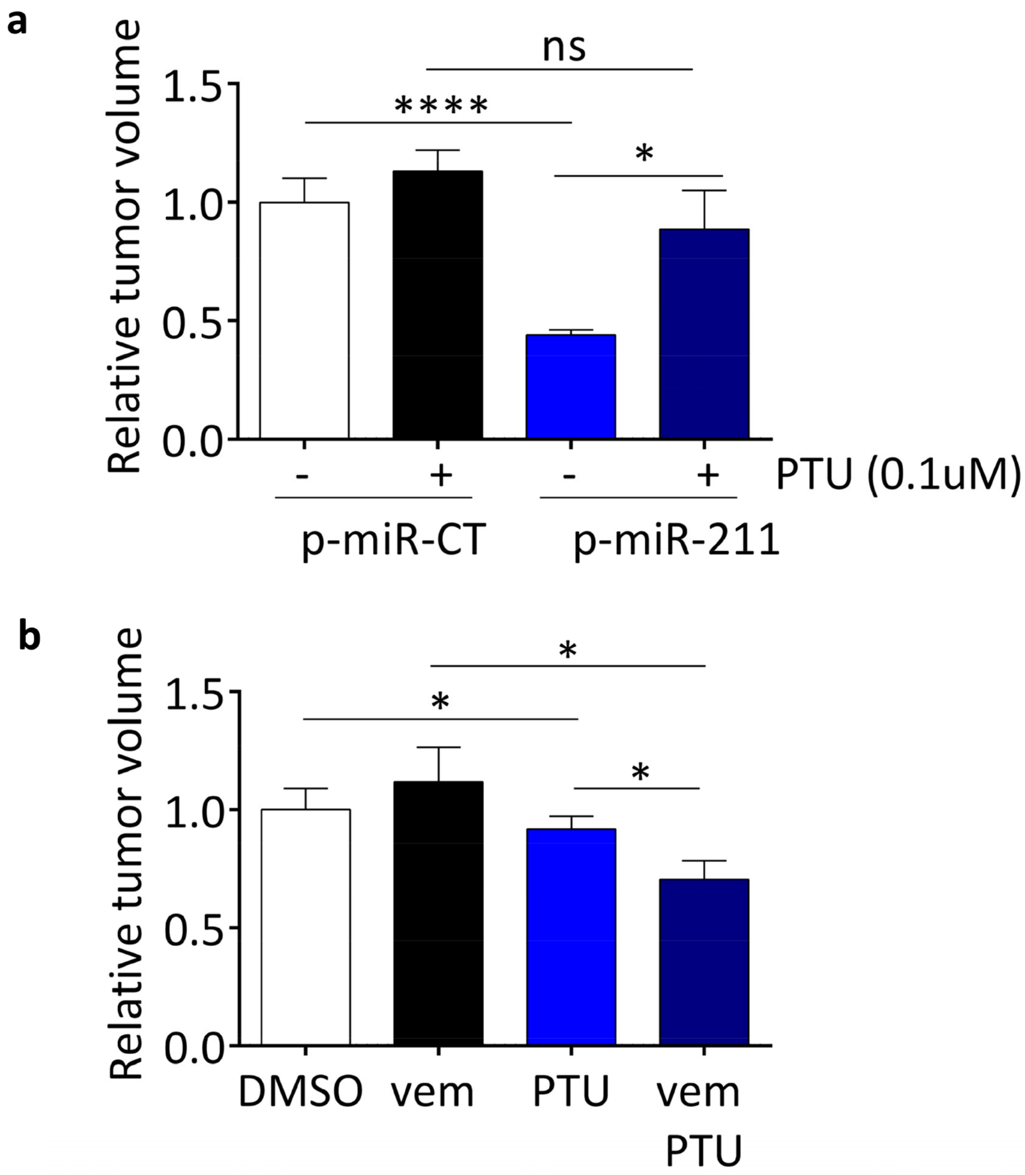

Supplementary Figure 28: The effects of miR-211 over-expression on 501 Mel cell proliferation are dependent on its pro-pigmentation ability. (a) 501 Mel cells that stably over-express a control miRNA (p-miR-CT, white and black), or miR-211 (p-miR-211, blue and dark blue) were treated with DMSO or 0.1uM PTU for 48h. They were then injected into the yolk sac of 48hpf stage zebrafish embryos. 48h later, the masses of the xenografted tumors were measured. **(b)** 501 Mel cells that stably over-express miR-211 were treated with DMSO, 0.2uM vemurafenib, 0.1uM PTU or 0.2uM vemurafenib plus 0.1uM PTU. They were then injected into the yolk sac of 48hpf stage zebrafish embryos. 48h later, the masses of the xenografted tumors were measured. The graphs represent the mean±SEM of 3 independent experiments. *p<0.05, ****<0.0001.

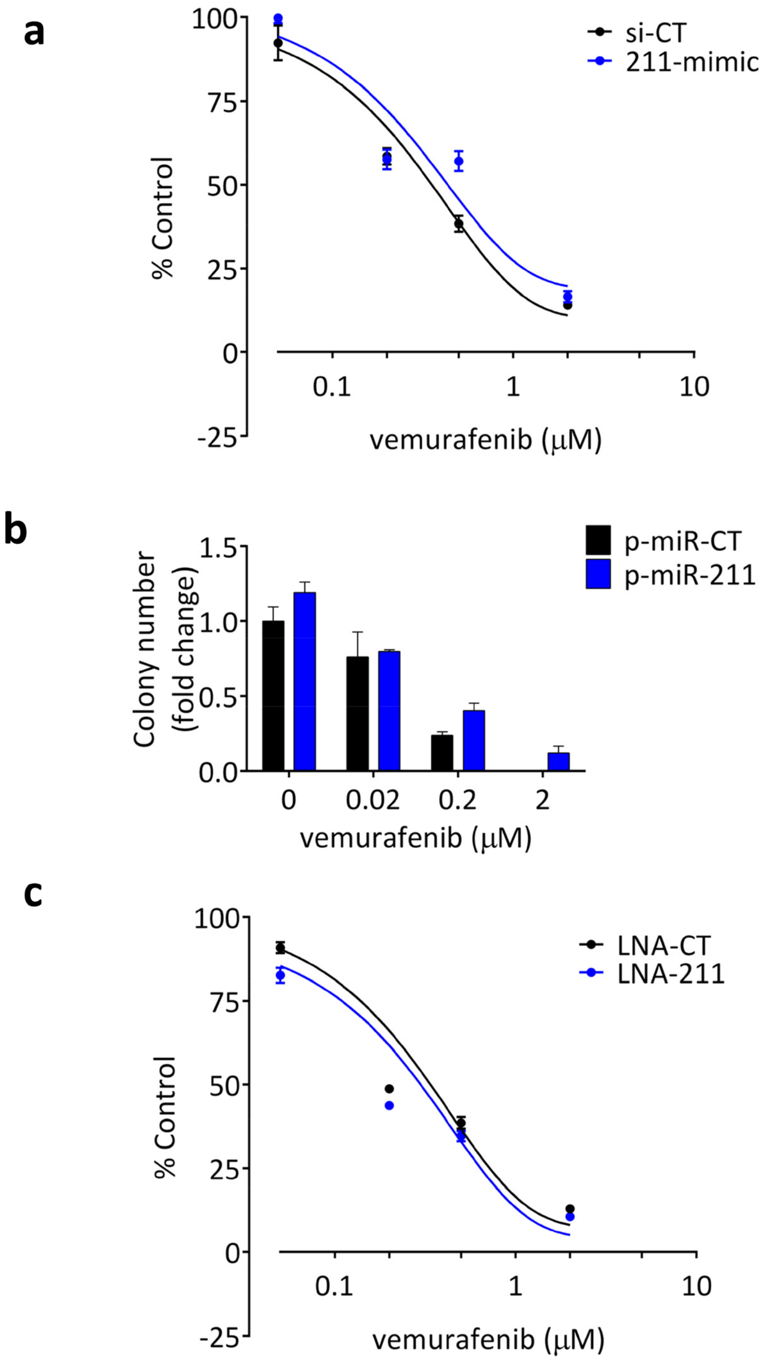

Supplementary Figure 29: Effects of 211-mimic and LNA-211 on the growth of 501 Mel cells. **(a)** Growth curve of 501 Mel cells transfected with si-CT (black) and 211-mimic (blue) in presence of increasing concentrations of vemurafenib. **(b)** Clonogenicity assay performed on 501 Mel cells transfected with si-CT (black) and 211-mimic (blue) in presence of DMSO or 0.02, 0.2 and 2uM vemurafenib. **(c)** Growth curve of 501 Mel cells transfected with LNA-CT (black) and LNA-211 (blue) in presence of increasing concentrations of vemurafenib. The graphs represent the mean±SEM of 3 independent experiments.

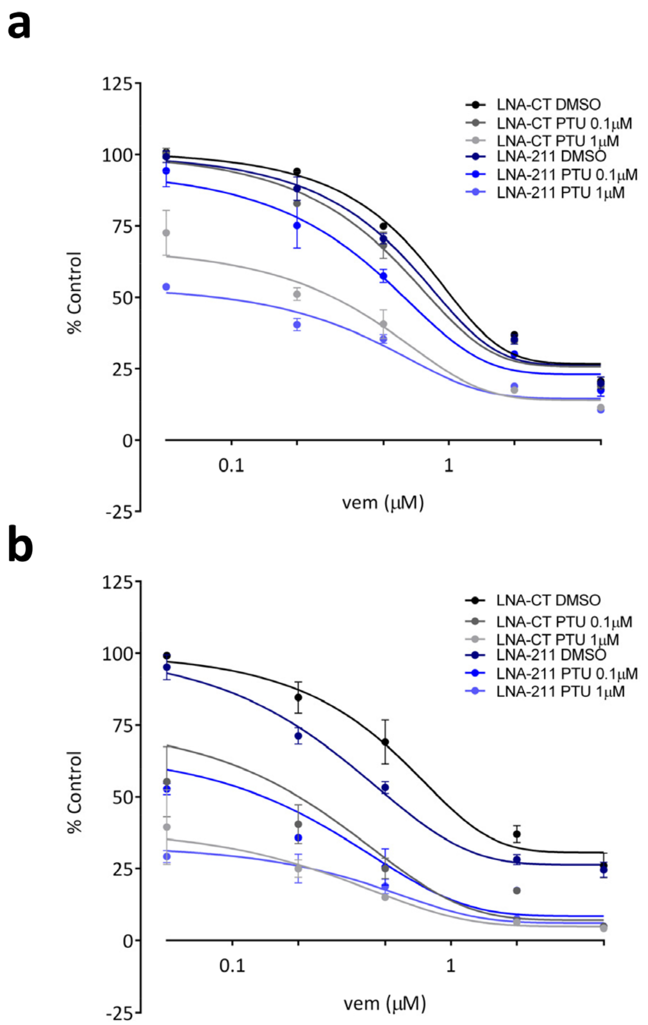

Supplementary Figure 30: LNA-211 cooperates with PTU in increasing the sensitivity of melanotic cells to vemurafenib. **(a)** Growth curve of 501 Mel cells transiently transfected with LNA-CT or LNA-211 and treated with increasing concentrations of vemurafenib in presence of DMSO vehicle, 0.1uM or 1uM PTU. **(b)** Growth curve of SK-Mel-5 transiently transfected with LNA-CT or LNA-211 and treated with increasing concentrations of vemurafenib in presence of DMSO vehicle, 0.1uM or 1uM PTU. The graphs represent the mean±SEM of 3 independent experiments.

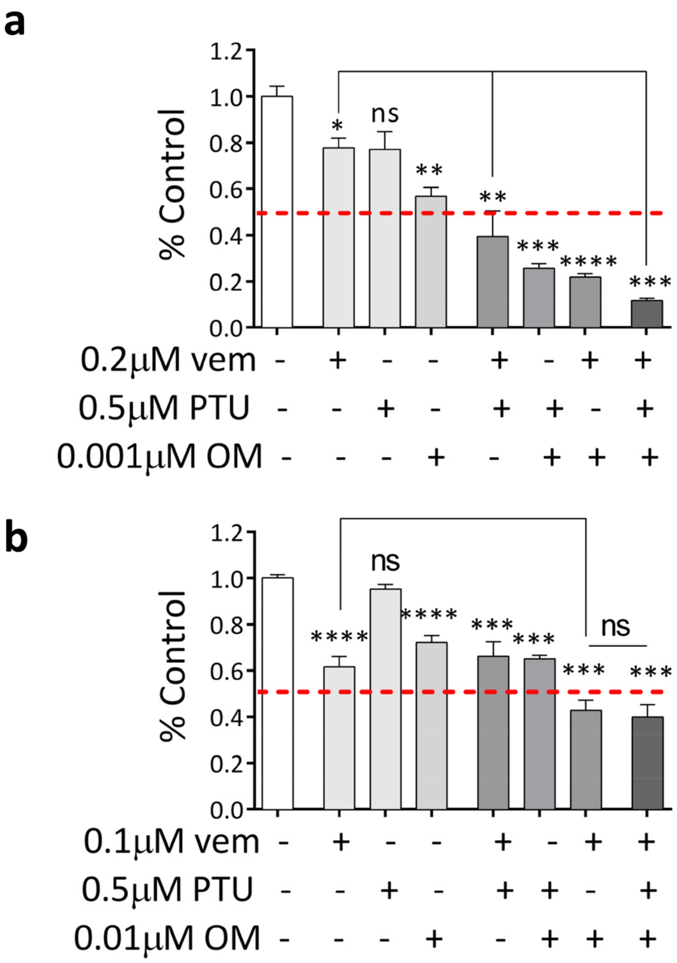

Supplementary Figure 31: Cooperation among vemurafenib, PTU and oligomycin. (a) Cell number upon the treatment of SK-Mel-5 cells with 0.2uM vemurafenib, 0.5uM PTU and 0.001uM OM or their combination for one week. **(b)** Cell number upon the treatment of A375 cells with 0.1uM vemurafenib, 0.5uM PTU and 0.01uM OM or their combination for one week. The graphs represent the mean±SEM of 3 independent experiments. *p<0.05, **p<0.01, ***p<0.001, ****<0.0001.

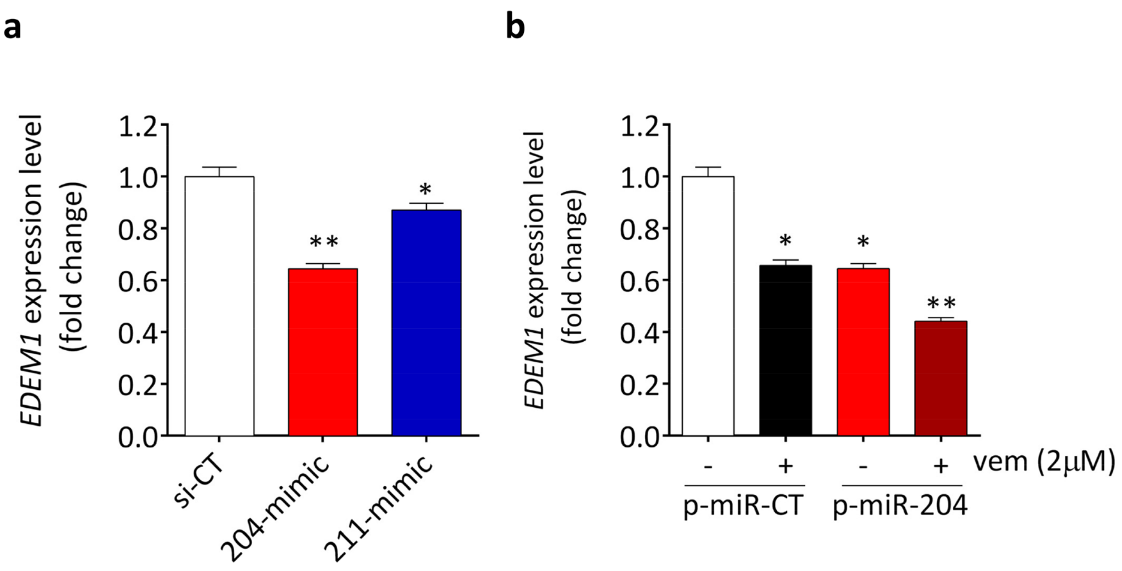

Supplementary Figure 32: EDEM1 targeting by miR-204 in A375 cells. (a) *EDEM1* levels 24h after the transient transfection of 204-mimic and 211-mimic. **(b)** *EDEM1* levels in A375 cells that stably over-express a control miRNA (p-miR-CT) or miR-204 (p-miR-204) and were exposed to vehicle (DMSO) or 2uM vemurafenib for 24h. miR-204 is able to down-regulate *EDEM1* and increases vemurafenib effect. The graphs represent the mean±SEM of 3 independent experiments. *p<0.05, **p<0.01.

Supplementary Table 1: Fingerprinting of vemurafenib-resistant clones and populations obtained from the parental lines A375, 501 Mel and SK-Mel-28

Parental cell lines	C or P	% homology
A375	C1	96
	C2	96
	C3	96
	P1	96
	P2	96
501 Mel	P1	84
SK-Mel-28	C1	82
	C2	82
	P1	82

In order to perform the fingerprinting analysis of vemurafenib-resistant clones (C) and populations (P), Short Tandem Repeats (STR) markers were detected on genomic DNA using the AmpFLSTR Identifiler PCR Amplification kit. The experimental steps that were followed are described in details in ref PMID 25685929. In all the cases, we obtained that the STR profile of the vemurafenib-resistant clone/population is a >80% match with the STR profile of the corresponding parental line, which, according to the recommendations of the International Cell Line Authentication Committee (ICLAC, http://iclac.org/resources/match-criteria-worksheet/), is consistent with cell identity. Therefore, we can conclude that the vemurafenib-resistant clones and populations that we have generated are not the product of cross-contamination.

Supplementary Table 2: List of the alterations associated with acquired resistance to BRAF inhibitors that have been searched for in the clones and populations listed in Figure 1a and of the primers used to detect them

Mutated gene	Residue	Exon	PCR forward primer	PCR reverse primer
BRAF	V600	15	TCATAATGCTTGCTCTGATAGGA	GGCCAAAAATTTAATCAGTGGA
	L505	12	CATGGAACAAACAAGGTTGG	AGTTGCTACCACTGGGAACC
NRAS	G12	2	CTGGTTTCCAACAGGTTCTTGC	CTACCACTGGGCCTCACCT
	Q61	3	CATACTGGATACAGCTGGAC	TGACTTGCTATTATTGATGG
	A146	4	TGCCCAGGCTAATCTCAAAC	TCACTTGAACCCAAGAGACAGA
KRAS	G12	2	GCCGCCGCAGAACAGCAGTC	GACCCTGACATACTCCCAAG
	G13	2	GCCGCCGCAGAACAGCAGTC	GACCCTGACATACTCCCAAG
	K117	4	GACAAAAGTTGTGGACAGGT	TAGCATAATTGAGAGAAAAACTG
MEK1	Q56	2	GCCTCCCACTTTGATTATCTG	CACACCAGCCTGGGCAACAAG
	K57	2	GCCTCCCACTTTGATTATCTG	CACACCAGCCTGGGCAACAAG
	V60	2	GCCTCCCACTTTGATTATCTG	CACACCAGCCTGGGCAACAAG
	C121	3	CCTGTTTCTCCTCCCTCTACC	ACACCCACCAGGAATACTGC
	P124	3	CCTGTTTCTCCTCCCTCTACC	ACACCCACCAGGAATACTGC
	G128	3	CCTGTTTCTCCTCCCTCTACC	ACACCCACCAGGAATACTGC
	E203	6	TAACGGACTCCTTCCTGTGG	TCCTCCCTCACTTCTTGTCC
MEK2	V35	2	AACCCCTCAAGTGCAGGAAT	CTGTTTCCAGGGGGACCTTC
	L46	2	AACCCCTCAAGTGCAGGAAT	CTGTTTCCAGGGGGACCTTC
	F57	2	AACCCCTCAAGTGCAGGAAT	CTGTTTCCAGGGGGACCTTC
	Q60	2	AACCCCTCAAGTGCAGGAAT	CTGTTTCCAGGGGGACCTTC
	C125	3	CTTGCAGCTGATCCACCTTG	GGTCTTCCTTCTCCCCAACA
	N126	3	CTTGCAGCTGATCCACCTTG	GGTCTTCCTTCTCCCCAACA
RAC1	P29	2	ATGTGATGGCTCCTGACTCTA	CCACCCCCAGCCCTATTTT

Amplified gene	PCR forward primer	PCR reverse primer
BRAF	ACCTCAGCAGTTACAAGCCT	CACTGGGAACCAGGAGCTAA
	GATATTGCACGACAGACTGCA	AGCATCCTTATGTTCCTGGACA

Overexpressed gene	qRT-PCR forward primer	qRT-PCR reverse primer
Total BRAF	CAGCACCTACACCTCAGCAG	GATACAAGCTGGAGCCCTCA
COT	CAAGTGAAGAGCCAGCAGTTT	GCAAGCAAATCCTCCACAGTTC
DCT	CCTTTCTTCCCTCCAGTGAC	AGCCAACAGCACAAAAAGAC
EGFR	CAGTTGGGCACTTTTGAAGAT	TGAGGGCAATGAGGACATAAC
FOXD3	CCAATTTCCTTTCCCCTGAG	CCATCCCCACGGTACTAAGA
IGF1R	GAACTACCAAATGGCGAGAT	AAGCTTCAGTGGAACAAACT
MCL-1	GGGCAGGATTGTGACTCTCATT	GATGCAGCTTTCTTGGTTTATGG
PAX3	GCTGCGTCTCCAAGATCC	TTTCCCAGCTGAACATGC
PDGFR	TTCCATGCCGAGTAACAGAC	CGTTGGTGATCATAGGGGAC
PTEN	GTTTACCGGCAGCATCAAAT	CCCCCACTTTAGTGCACAGT

Supplementary Table 3: miRNAs differentially expressed in A375 vemurafenib vs A375 DMSO

See Supplementary File 1

Supplementary Table 4: miRNAs differentially expressed in A375 vemurafenib vs C2 vemurafenib

See Supplementary File 2

Supplementary Table 5: miRNAs not differentially expressed in C2 vemurafenib vs C2 DMSO and expressed at 100 reads in at least 1 out of the 4 conditions

See Supplementary File 3

Supplementary Table 6: List of the microarray probes for *TRPM1*, *TRPM3* and *MITF* present in the datasets available at GEO under the indicated accession numbers

GEO accession number	*TRPM1* *probes*	*TRPM3* *probes*	*MITF* *probes*
GSE 3189	206479_at<br>214410_at	211422_at<br>216452_at<br>220463_at	207233_s_at
GSE 7553	237069_s_at<br>237070_at<br>240386_at<br>206479_at<br>214410_at	211422_at<br>216452_at<br>220463_at	207233_s_at<br>226066_at<br>1554874_at
GSE 12391	#3272	#13726<br>#25034<br>#29710<br>#37634<br>#41172	#5644<br>#10661<br>#29754<br>#42187
GSE 65904	ILMN_1791653	ILMN_2302983<br>ILMN_1740622<br>ILMN_1815938	ILMN_2304186<br>ILMN_1657854<br>ILMN_1761324

Supplementary Table 7: List of validated miR-211 and miR-204 targets that can explain their anti-motility function in melanoma and in other cancers

Gene	Validated targets of miR-211 and miR-204		PubMed ID	Real-time PCR primers (Fw, Rv)
AP1S2	X		24039954	CCTTGAGTGGCGAGATCTGA<br>CCTGATCCTCAATAGCACAGC
BDNF		X	23285024	GGCTTGACATCATTGGCTGAC<br>CATTGGGCCGAACTTTCTGGT
EPHB2		X	23204229	TTGGGCTCTCACGCTTTCTA<br>AGGTGAACTTCCGGTACTGG
EZRIN		X	25055875<br>21416062	TGTGGTACTTTGGCCTCCAC<br>GTTTCTGGGTGATGTCCTGG
FOXC1		X	21400511	CCGGACAAGAAGATCACCCT<br>ATGTTGTAGGAGTCCGGGTC
IGF2R	X		21109473	CTGCCGCTATGAAATTGAGTGG<br>CGCCGCTCAGAGAACAAGTT
NFAT5	X		21109473	GGGTCAAACGACGAGATTGTG<br>GTCCGTGGTAAGCTGAGAAAG
NUAK1	X	X	25412236<br>23934065	TTGCTGACTTTGGGCTTTCC<br>GTAAAGCAACACACCCAGGG
RAB22A	X	X	27237979<br>25294901<br>26134825	GAACGATTTCGTGCCTTAGC<br>TCGAAGCTCTTTCACCCAAT
TGFbetaR2	X		21109473	AACGGTGCAGTCAAGTTTCC<br>GGCTTCTCACAGATGGAGGT
TRKB		X	24321270	GGGACACCACGAACAGAAGTA<br>ACCACAGCATAGACCGAGAGA

Supplementary Table 8: mRNAs differentially expressed upon 204-mimic transfection

See Supplementary File 4

Supplementary Table 9: mRNAs differentially expressed upon 211-mimic transfection

See Supplementary File 5

Supplementary Table 10: List of predicted and validated miR-204/miR-211 targets among the mRNAs belonging to the GO "protein transport, regulation of nuclease activity, ER-nucleus signaling pathway, response to endoplasmic reticulum stress and regulation of translation"

NM	Target	hsa-miR-204				hsa-miR-211			
		5'UTR	ORF	3'UTR	Validated (methods and PubMed ID)	5'UTR	ORF	3'UTR	Validated (methods and PubMed ID)
NM_003916	*AP1S2*	-	-	x	R,P 21282569	-	-	x	R,P 24039954
NM_014445	*SERP1*	-	-	x	R,P 21282569	-	-	x	-
NM_016131	*RAB10*	-	-	x	-	-	-	x	-
NM_014674	*EDEM1*	-	-	x	R,P 21282569	-	-	x	-
NM_020673	*RAB22A*	-	-	x	R,P,W 21282569 25294901 25429829 26134825	-	-	x	R,P,W 24039954
NM_001695	*ATP6V1C1*	-	-	x	-	-		x	-
NM_015088	*TNRC6B*	-	x	x	-	-	x	x	-
NM_004094	*EIF2S1*	-	-	-	-	-	-	-	-

The reported predictions (x) were obtained from miRWalk2.0 (PMID 26226356) considering:
-a 6mer minimum seed length and/or a 0.05 p value as input parameters;
-a prediction by at least 6 out of 12 algorithms for the 3'UTR;
-a prediction by at least 4 out of 7 algorithms for the ORF;
-a prediction by at least 3 out of 6 algorithms for the 5'UTR.
R: reporter assay; P: qRT-PCR; W: Western Blot.

Supplementary Table 11: NYU patient characteristics

NYU Patient Characteristics			
	**Amelanotic (n=26)**	**Melanotic (n=51)**	**Total (n=77)**
**Sex**			
Male	18	33	51
Female	8	18	26
**Age at Stage IV Diagnosis**			
Mean (SD)	55.7 (14.1)	57.5 (13.8)	56.9 (13.9)
Median	54.7	57.4	57.1
**Treatment**			
Single agent targeted therapy (i.e. vemurafenib)	18	38	56
Dual agent targeted therapy (i.e. dabrafenib + trametinib)	8	11	19
Targeted + Immunotherapy (i.e. dabrafenib + ipilimumab)	0	2	2
**Metastasis Location**			
Skin/Subcutaneous	4	7	11
Soft Tissue	8	13	21
Nodal	10	22	32
Visceral	4	7	11
Brain	0	2	2

Supplementary Table 12: List of PCR and real-time PCR primers

Gene	Forward primer	Reverse primer
ATP6V1C1	TCCTGGGGAGAAAACCTGTC	AAGACATCCAACGTGCCAAC
ATPA1	CTCAGATGTGTCCAAGCAAG	GTCAGTGCCCAAGTCAATG
Total *BRAF*	CAGCACCTACACCTCAGCAG	GATACAAGCTGGAGCCCTCA
[Δ3-10] *BRAF*	CAATATATCTGGAGAAAACACTTGG	TGAGGTGTAGGTGCTGTCAC
Cas9	GTACCCCACCATCTACCACC	GGATGAACAGCTTGTCCACG
DCT	CCTTTCTTCCCTCCAGTGAC	AGCCAACAGCACAAAAAGAC
EDEM1	CAATGAAGGAGAAGGAGAC	CAATGTGTCCCTCTGTTGTG
EIF2S1	TGACTACGACAACCCTGGAG	ACACACCCCTCTTTTCCTCA
EGFR	CAGTTGGGCACTTTTGAAGAT	TGAGGGCAATGAGGACATAAC
GAPDH	CGCTCTCTGCTCCTCCTGTT	CCATGGTGTCTGAGCGATGT
eYFP	GGTCTTGTAGTTGCCGTCGT	CACATGAAGCAGCACGACTT
mCherry	CAGAACGGCTGCATCATCTA	GGGTACAGCATCTCGGTGTT
miR-204 family	GGGTACAGCATCTCGGTGTT	TGAATCGAGCACCAGTTACGC
MITF	TGACCGCATTAAAGAACTAGG	GTGCTCCAGTTTCTTCTGTCG
MLANA	CTCTTACACCACGGCTGAA	AGACTCCCAGGATCACT
PBGD	TCCAAGCGGAGCCATGTCTG	AGAATCTTGTCCCCTGTGGTGGA
PDGFRbeta	TTCCATGCCGAGTAACAGAC	CGTTGGTGATCATAGGGGAC
PGC1α	GTCACCACCCAAATCCTTAT	CGGTGTCTGTAGTGGCTTGA
RAB10	AGAAGACGTACGACCTGCTT	CTGGCCTGCTGTATCCCATA
SDHA	CCACTCGCTATTGCACACC	CACTCCCCATTCTCCATCA
SERP1	TGTCTGTGGTTCTGCAATTT	TGACTCATGAAGAAACCTTGGA
SNO-110	TGATGTCTCCATGTCTCTGAGCAA	TGAATCGAGCACCAGTTACGC
SNO-44	CCTGGATGATGATAAGC	TGAATCGAGCACCAGTTACGC
SNO-55	AGCCAACCTTGGAGAGCTGAGC	TGAATCGAGCACCAGTTACGC
TNRC6B	AAGCTGTCTCCCTCTGGTTC	TGTGCAGACCTCTCGATTGT
TRPM1	TGCGAAGGCTGCTGGAAA	CAAGACGATGGACACCACGTTAGG
TRPM3	GGAGCAGAGGTGAAACTTCG	CCCATCACAGACAACCACTG
TYR	GATGAGTACATGGGAGGTCAGC	GTACTCCTCCAATCGGCTACAG
TYRP1	GGACCAGCTTTTCTCACAT	GAATCAAAGTTGCTTCTGGA

Supplementary Table 13: Sequence of the siRNAs, miRNA mimics and LNAs used

	Gene Name	Sequence	
		sense (5'-3')	antisense (5'-3')
**siRNAs**	*AP1S2 #1*	GUGGCGAGAUCUGAAGAUUUU	AAUCUUCAGAUCUCGCCACUU
	AP1S2 #2	CAGUGUCUGUGAACUAGAUUU	AUCUAGUUCACAGACACUGUU
	BRAF	AGAAUUGGAUCUGGAUCAUUUUU	AAAUGAUCCAGAUCCAAUUCUUU
	EDEM1 #1	CGGGGACCCUUCAAAUCUGUU	CAGAUUUGAAGGGUCCCCGUU
	EDEM1 #2	CUGGACUCCUUCUAUGAAUUU	AUUCAUAGAAGGAGUCCAGUU
	MITF	GUGCCCAGGCAUGAACACACAUU	UGUGUGUUCAUGCCUGGGCACUU
	si-CT	UUCUCCGAACGUGUCACGUUU	ACGUGACACGUUCGGAGAAUU
**mimics**	miR-204-5p	UUCCCUUUGUCAUCCUAUGCCUUU	AGGCAUAGGAUGACAAAGGUUAUU
	miR-211-5p	UUCCCUUUGUCAUCCUUCGCCUUU	AGGCGAAGGAUGACAAAGGUUAUU
**LNAs**	LNA-204	AGGCATAGGATGACAAAGGGAA	
	LNA-211	AGGCGAAGGATGACAAAGGGAA	
	LNA-CT	GTGTAACACGTCTATACGCCCA	
